# Interfacial Hydrogen-Bond Dynamics in Transition Metal Compounds

**DOI:** 10.1021/acs.chemmater.6c01283

**Published:** 2026-07-25

**Authors:** David Kumar Yesudoss, Hao-En Lai, Naresh C. Osti, Bright Ngozichukwu, Joshua Ferguson, Eugene Mamontov, Perla B. Balbuena, Abdoulaye Djire

**Affiliations:** † Artie McFerrin Department of Chemical Engineering, 14736 Texas A&M University, College Station, Texas 77843, United States; ‡ Neutron Scattering Division, 6146 Oak Ridge National Laboratory, Oak Ridge, Tennessee 37831, United States; § Department of Chemistry, Texas A&M University, College Station, Texas 77843, United States; ∥ Department of Materials Science and Engineering, Texas A&M University, College Station, Texas 77843, United States

## Abstract

Understanding how water behaves when confined within atomic layers of active transition-metal carbides, nitrides, and carbonitrides is essential for uncovering the fundamental principles needed to engineer solid–liquid interfaces at the atomic scale. Yet, how lattice element chemistry and surface termination groups collectively regulate the structure and mobility of such interlayer water remains poorly understood. Here, we present a composition-controlled investigation of interlayer water dynamics in layered transition-metal nitride, carbide, and carbonitride systems using a systematic integration of quasi-elastic neutron scattering (QENS), ab initio molecular dynamics (AIMD) simulations, and density functional theory (DFT) calculations. QENS measurements show that nitride-rich systems host mobile, translationally diffusing water with thermally activated self-diffusion coefficients on the order of 10^–10^ m^2^ s^–1^, whereas mixed C/N lattices confine water to localized, nontranslational motion that is insensitive to temperature. AIMD and DFT reveal that lattice C/N chemistry and surface functional group composition reshape the first hydration layer by modulating the surface electronic structure and termination-dependent hydrogen-bond networks, leading to pronounced differences in water ordering and thermal resilience. On the other hand, fully carbide systems exhibit intermediate behavior, highlighting that water mobility is not primarily controlled by the hydration level alone but by the coupling between lattice composition and surface chemistry. Overall, this study establishes how surface chemistry and lattice composition jointly control interfacial hydrogen bond dynamics, offering a mechanistic framework for designing transition-metal layered materials with tailored interfacial transport properties.

## Graphical abstract

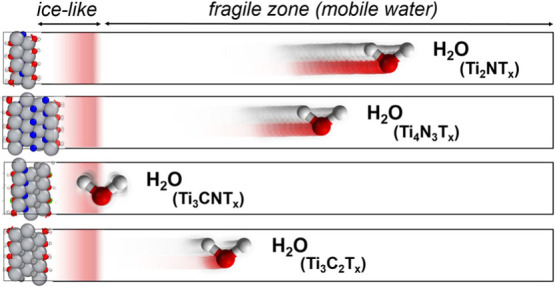

## Introduction

Understanding the chemistry of water confined within nanoscale transition-metal compounds is central to a broad range of electrochemical technologies, from charge storage to catalytic reactions.^1–5^ The structure and dynamics of confined water differ significantly from bulk water. These confined molecules, stabilized by hydrogen bonding and van der Waals forces, play a critical role in ion transport and solid–liquid interfacial chemistry in layered and porous materials. Despite its importance, a molecular-level understanding of how interlayer water is organized, how strongly it is bound, how it exchanges, and how it transitions between localized and long-range motion at the surfaces of active transition-metal layers is still lacking.

Prior studies on layered oxides, clays, and functionalized carbon frameworks have demonstrated that confinement can produce water populations with distinct dynamical regimes, including strongly bound interfacial layers and more mobile, bulk-like components.^6–14^ These findings established that interfacial chemistry, surface polarity, functional groups, and lattice charge distribution often dominate over geometric confinement alone in determining water structure and mobility. However, extending this understanding to emerging electrochemically active transition-metal carbides and nitrides remains challenging because these materials present both tunable lattice chemistry and complex surface termination environments that can reorganize the hydrogen-bond network.

Resolving water motion in such hydrogen-rich, nanoconfined environments requires techniques that directly probe hydrogen dynamics over relevant length and time scales. In this regard, QENS has emerged as a powerful tool for investigating the structure and dynamics of hydrogen-bearing species,^15–18^ including water, in confined and interfacial environments due to the exceptionally large incoherent neutron scattering cross-section of hydrogen (σ_s_(H) = 82.02 b), while contributions from heavier elements such as carbon, oxygen, fluorine, aluminum, and titanium are comparatively weak.^19^ Also, QENS enables quantitative extraction of diffusion coefficients, residence times, and activation energies, as well as distinguishing localized from long-range translational dynamics. More importantly, QENS can access ensemble-averaged motions inside layered structures under conditions where conventional spectroscopies provide limited dynamical information. Nevertheless, QENS alone cannot fully resolve the atomistic origins of the observed dynamical regimes, such as water orientation relative to surface terminations, hydrogen-bond lifetimes, or specific adsorption motifs that immobilize or mobilize interlayer water. However, when complemented with ab initio molecular dynamics (AIMD), a direct route to these mechanistic descriptors can be obtained by explicitly modeling interfacial hydrogen-bond networks and water-surface interactions. Furthermore, QENS quantifies macroscopic transport metrics, while AIMD provides the microscopic interaction picture needed to rationalize why certain compositions favor mobile water and others stabilize structured, temperature-robust networks. When combined with AIMD, QENS provides complementary experimental and atomistic descriptions of interfacial water dynamics in transition-metal carbides, nitrides, and carbonitrides.

The critical role of confined or structural water in modulating electrochemical behavior has been demonstrated in some layered transition metal systems but not in emerging nitride MXenes (MNenes). For example, in tungsten oxide hydrates (WO_3_.*n*H_2_O), the presence of structural water has been shown to fundamentally alter charge storage mechanisms, shifting the response from diffusion-limited behavior in anhydrous WO_3_ to pseudocapacitive behavior in hydrated WO_3_.2H_2_O, while simultaneously suppressing electrochemical deformation.^20^ However, a largely unresolved question is how the identity of the lattice light element (C and/or N) in layered transition-metal frameworks modulates interlayer water chemistry. Because the M–N bonding network can differ electronically and chemically from M–C, alterations in surface charge distribution, surface termination group preference, and water adsorption strength may be observed, thereby reshaping the interfacial hydrogen-bond network and the balance between bound and mobile water. In this regard, layered transition-metal carbides, nitrides, and carbonitrides with tunable compositions can provide an ideal platform to interrogate this effect in a controlled manner. Among layered transition metal compounds, MXenes, a family of two-dimensional carbides, nitrides, and carbonitrides derived from MAX phases, have attracted considerable attention due to their high electrical conductivity, hydrophilicity, and structural tunability. MXenes are generally described by the formula M_*n*+1_X_*n*_T_*x*_, where M is an early transition metal, X is carbon and/or nitrogen, and T_*x*_ represents surface terminations such as –O, –OH, and –F introduced during synthesis.^1,21–26^ These surface terminations render MXenes highly hydrophilic, enabling spontaneous intercalation of water molecules between adjacent layers under ambient conditions. Therefore, MXenes can be ideal model candidates for investigating the interplay between lattice light elements (C and/or N), surface chemistry, and interlayer water structure.

Here, we present a composition-controlled study of interlayer water structure and dynamics across layered transition-metal systems such as Ti_2_NT_*x*_, Ti_4_N_3_T_*x*_, Ti_3_CNT_*x*_, and Ti_3_C_2_T_*x*_ that vary in lattice C/N chemistry and surface termination environments. Using QENS to quantify the translational and localized motions of interlayer water and derive activation energies, we report on the strength of water-surface coupling. Experimentally, we observe significantly higher water content and long-range translational diffusion in the nitride-rich Ti_2_NT_*x*_ and Ti_4_N_3_T_*x*_ systems, whereas coexistence of C and N in the lattice (Ti_3_CNT_*x*_) contains only a small population of interlayer water that exhibits fully localized dynamics with negligible temperature dependence. We then employ AIMD simulations to resolve atomistic descriptors of interlayer water, including interfacial ordering, hydrogen bond lifetimes, and preferred adsorption motifs near specific surface terminations. AIMD simulations reveal pronounced differences in how lattice C vs N environments regulate the first interfacial water layer, leading to distinct hydrogen-bond network stability and temperature responses. Overall, the systematic integration of computation and experiment reveals how subtle changes in lattice chemistry and surface functionality govern whether interlayer water exhibits long-range diffusion or becomes locked into localized temperature-resilient networks.

## Experimental Section

### Ti_3_CNT_*x*_ Synthesis

Ti_3_CNT_*x*_ MXene was prepared via an in situ HF etching process. First, 0.8 g of LiF was dissolved in 10 mL of 9 M H_2_SO_4_ solution and stirred for 15 min. Afterward, 0.5 g of Ti_3_AlCN powder (Nanochemazone, Canada) was slowly added to the solution, which was left to stir magnetically for 24 h at room temperature. Following this, the mixture was washed with deionized (DI) water by repeated cycles of centrifugation (5 min at 3500 rpm) and rinsing. This washing process was repeated four to five times, until the mixture reached a neutral pH. At neutral pH, the supernatant containing a dark colloidal suspension of Ti_3_CNT_*x*_ MXene was left untouched for 1 h to allow any unetched MAX powders to settle. The supernatant was collected and vacuum-filtered to obtain final Ti_3_CNT_*x*_ powders.

### Ti_4_N_3_T_*x*_ Synthesis

The Ti_4_AlN_3_ MAX phase was synthesized by mixing Ti (Sigma-Aldrich, 99.7%, 100 mesh), Al (Sigma-Aldrich, 99%, 30 μm), and TiN (Sigma-Aldrich, 99%, 3 μm) powders in a molar ratio of 1:1.2:2.05. The powders were ground together in an agate mortar for 10 min. The resulting mixture was then sintered in a tube furnace (CM Furnace Inc. 1730-20 HT) at 1400 °C for 30 h, with a ramp rate of 10 °C per minute, under a continuous flow of argon (Ar) gas (Airgas, Ultra High Purity). After sintering, the Ti_4_AlN_3_ pellet was ground in an agate mortar in preparation for the etching process. Next, the Ti_4_AlN_3_ MAX was molten salt treated to selectively etch the Al from Ti_4_AlN_3_ via oxygen-assisted molten salt fluoride (MSF) etching. The MSF mixture, composed of KF (Alfa Aesar, 99%), LiF (Alfa Aesar, 325 mesh, 98.5%), and NaF (Alfa Aesar, 99%) in a 59:29:12 mass ratio, was mixed with Ti_4_AlN_3_ powder in a 1:1 mass ratio. This mixture was ground in an agate mortar and transferred to a crucible boat, which was then placed in a quartz tube furnace (ATS Series 3210). The furnace was heated to 550 °C at a rate of 10 °C/min and held for 5 h under an argon flow. After 5 h, the argon flow was halted, and the outlet tubing of the furnace was exposed to atmospheric air for 1 h. Following this, the furnace was resealed, and etching continued at 550 °C for another 2 h. The furnace was then turned off and allowed to cool to room temperature. The resulting material was collected and ground to achieve uniform particle size.

Approximately 0.5 g of the etched Ti_4_AlN_3_ sample was acid-washed by stirring it in 20 mL of 4 M formic acid (Sigma-Aldrich, 95%) for 1 h at 500 rpm. The suspension was filtered through a 0.20 μm polycarbonate membrane (Whatman Nucleopore) and rinsed continuously with deionized water (18.2 MΩ·cm, Milli-Q) until the pH reached 6. The washed Ti_4_N_3_T_*x*_ sample was dried overnight in a vacuum oven at 50 °C, transferred to a vial, and then stored in a glovebox. To obtain delaminated Ti_4_N_3_T_*x*_ MXene, 10 mL of DI water was mixed with the Ti_4_N_3_T_*x*_ sample and stirred for 4 h. The suspension was sonicated for 30 min and diluted with water to a total volume of 50 mL. Centrifugation was performed at 3500 rpm for 30 min, followed by decanting of the supernatant. This process was repeated until the pH reached 6. Afterward, the suspension was allowed to settle for 1 h, and the remaining supernatant was filtered through a 0.20 μm polycarbonate membrane to obtain Ti_4_N_3_T_*x*_ powders.

### Ti_2_NT_*x*_ Synthesis

The Ti_2_AlN MAX phase was synthesized by grinding powders of Ti (Sigma-Aldrich, 99.7%, 100 mesh) and AlN (Sigma-Aldrich, 99%, 3 μm) in a 2:1 molar ratio in an agate mortar for 10 min. The mix was then sintered in a tube furnace (CM Furnace Inc. 1730-20 HT) at 1600 °C for 1 h at a ramp rate of 10 °C min^–1^ under constant Ar flow (Airgas, Ultra High Purity). The resulting Ti_2_AlN pellet was ground in an agate mortar in preparation for etching. Ti_2_NT_*x*_ MXene was synthesized by removing the aluminum (Al) layer from Ti_2_AlN MAX phase using oxygen-assisted molten salt etching. The process began by thoroughly mixing 0.25 g of the Ti_2_AlN MAX phase with 0.25 g of a molten salt mixture (59 wt % KF, 29 wt % LiF, and 12 wt % NaF) using a pestle and mortar. The mixture was transferred to an alumina crucible and heated in a tube furnace to 550 °C at a rate of 10 °C/min under a flow of 360 SCCM argon (Ar). After reaching 550 °C, the temperature was maintained for 5 h. Halfway through the process, the argon flow was stopped, and the furnace inlet and outlet were sealed. The furnace was then cooled to 450 °C, held at this temperature for 1 h, and subsequently allowed to cool to room temperature. The etched material was extracted for further acid treatment. To remove the etched aluminum salts formed during the molten salt treatment, the etched Ti_2_AlN material was dispersed in 20 mL of 4 M formic acid and stirred at 600 rpm for 1 h. The suspension was vacuum-filtered through a 0.4 μm polycarbonate membrane (Whatman Nucleopore), and any remaining residual salts and formic acid were removed by washing the filter membrane with 1 L of deionized (DI) water. After filtration, the membrane was dried overnight in a vacuum oven at 50 °C. The dried material, referred to as multilayered Ti_2_NT_*x*_, was scraped from the membrane and set aside for delamination. To produce delaminated Ti_2_NT_*x*_ MXene, the multilayered Ti_2_NT_*x*_ was dispersed in 10 mL of DI water solution and stirred at 600 rpm for 4 h. The suspension was then centrifuge-washed at 3500 rpm with DI water until a neutral pH was reached. After this, the dispersion was sonicated for 1 h and allowed to rest for another hour to let any unetched MAX phase settle. The supernatant was carefully pipetted out and vacuum-filtered to collect delaminated Ti_2_NT_*x*_ powders.

### Quasi-Elastic Neutron Scattering Measurement

The dynamics of water in nitride MXenes and their corresponding MAX phases was investigated using QENS measurements, performed with the time-of-flight backscattering silicon spectrometer (BASIS) at the Spallation Neutron Source, Oak Ridge National Laboratory. The center wavelength of the incident neutron band of ca. 0.5 Å width was ∼6.4 Å, and Si (111) analyzer crystals were positioned for Bragg reflection of 88° (∼6.267 Å). The instrument configuration provides an analyzable dynamic energy transfer range of −100 to +100 μeV and an overall (averaged over all scattering angles) energy resolution of 3.5 μeV. The measurable momentum transfer (*Q*) ranged from 0.3 to 1.9 Å^–1^. For QENS measurements, approximately 1 g of each sample was loaded between two flat metal plates with an insert providing the sample thickness of ∼0.25 mm, sealed with indium metal, and placed at 90° relative to the incident neutron wave vector ( $\vec{K_{i}}$ ). The scattering vector $Q=\vec{K_{i}}-\vec{K_{f}}$ , where $\vec{K_{f}}$ is the wave vector of the scattered neutrons, was determined by the detector. The instrument resolutions were obtained for each sample individually at 35 K, and the energy-resolved “elastic” intensity scans were conducted over the temperature range of 35 to 300 K. Data reduction and transformation into 1D spectra were carried out using MantidPlot software.^19^ Subsequent data fitting and analysis were performed with the DAVE software package developed at the NIST Center for Neutron Research.^20^

The measured QENS intensity, *I*(*Q*,*E*), was modeled by the following expression

1 $$I\left(Q,E\right)=\left[X_{1}\left(Q\right)\delta \left(E\right)+\left(1-X_{1}\left(Q\right)\right)S\left(Q,E\right)+B\left(Q,E\right)\right]⊗R\left(Q,E\right)$$

where *X*_1_(*Q*) represents the contribution of elastic scattering, δ­(*E*) is the Dirac delta function corresponding to zero energy transfer (elastic component), *S*(*Q*,*E*) is the quasi-elastic model function, and *B*(*Q*,*E*) and *R*(*Q*,*E*) denote the background and instrumental resolution function, respectively. The quasi-elastic model function *S*(*Q*,*E*), measured in the QENS experiment, was assumed to be a Lorentzian. Taken together with the elastic signal and a linear background, it was convoluted with the instrumental resolution and fitted to the measured data. The half-width at half-maximum (HWHM), Γ­(*Q*), extracted from the Lorentzian fits, was analyzed as a function of *Q* using the jump-diffusion model

2 $$\Gamma \left(Q\right)=\frac{DQ^{2}}{1+DQ^{2}\tau_{o}}$$

where *D* is the diffusion coefficient and τ_*o*_ is the residence time between successive jumps. In the absence of long-range translational self-diffusivity, an average of the *Q*-independent HWHM of the Lorentzian function provides a localized jump rate of the water molecules as

3 $$\Gamma \left(Q\right)=\frac{0.658}{\left\langle \tau \right\rangle }$$

where the time expressed in nanoseconds corresponds to the width expressed in μeV.

## Results and Discussion

### MXene Synthesis

To study the structural and dynamical behavior of interlayer water within layers of 2D transition metal compounds, Ti_2_NT_*x*_, Ti_3_C_2_T_*x*_, Ti_4_N_3_T_*x*_, and Ti_3_CNT_*x*_, were synthesized from their parent Ti_*n*+1_Al­(N/C)_*n*_ ceramic phases of Ti_2_AlN, Ti_3_AlC_2_, Ti_4_AlN_3_, and Ti_3_AlCN, respectively. The Ti_2_NT_*x*_ and Ti_4_N_3_T_*x*_ MNenes were synthesized using an oxygen-assisted molten salt fluoride etching method, whereas Ti_3_CNT_*x*_ and Ti_3_C_2_T_*x*_ MXenes were synthesized using a mild etching method involving LiF and 9 M H_2_SO_4_. Ti_3_N_2_T_*x*_ was not included in this study because the corresponding Ti_3_AlN_2_ MAX precursor is not experimentally achievable, compared to Ti_2_AlN, Ti_4_AlN_3_, Ti_3_AlCN, and Ti_3_AlC_2_. Thus, the selected systems represent experimentally accessible Ti-based nitride, carbonitride, and carbide MXenes suitable for direct QENS comparison. The successful etching and phase purity of all MXenes were confirmed through X-ray diffraction (XRD) analysis. Figure 1a–d shows the XRD patterns of the synthesized MXenes. A little to no presence of the MAX phase (002) peak in the etched material confirms the high phase purity of the synthesized MXenes. Upon etching, a shift in the (002) peak was observed for Ti_2_NT_*x*_ and Ti_4_N_3_T_*x*_ MNenes, which indicates an increase in *d* spacing from 0.68 to 0.91 nm in Ti_2_NT_*x*_ and 1.16 to 1.43 nm in Ti_4_N_3_T_*x*_ *,* confirming successful etching and exfoliation (Figure 1a,b). The XRD patterns of the MNenes are consistent with previous reports, demonstrating successful MAX phase etching, effective removal of fluoride salts after acid washing, and subsequent delamination.^27–29^ For Ti_3_CNT_*x*_ MXene (Figure 1c), the shift of the (002) peak from ∼9.6° (corresponding to the Ti_3_AlCN MAX phase) to approximately 7.34° indicates an increase in the interlayer *d* spacing from 0.91 to 1.20 nm and structural expansion after successful Al layer etching. Similarly, Ti_3_C_2_Tx MXene showed *d* space expansion from 0.9 nm in the Ti_3_AlC_2_ MAX phase to 1.39 nm in the delaminated MXene, indicating successful etching of the “A” layer to form exfoliated 2D sheets.

**Figure 1:**
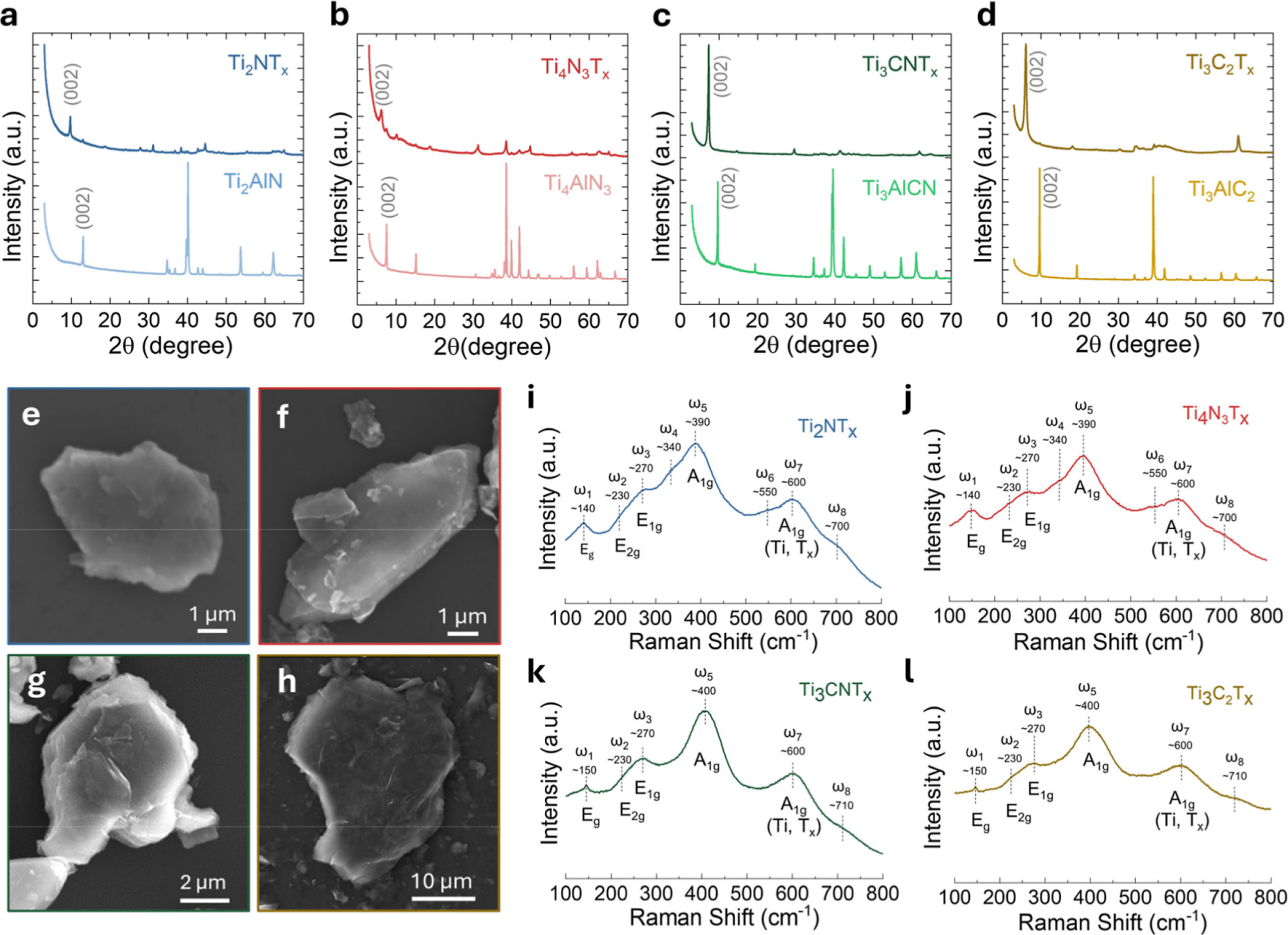
X-ray diffraction (XRD) patterns of delaminated MXenes and their corresponding MAX phases. (a) Ti_2_NT_*x*_ and Ti_2_AlN, (b) Ti_4_N_3_T_*x*_ and Ti_4_AlN_3_, (c) Ti_3_CNT_*x*_ and Ti_3_AlCN, (d) Ti_3_C_2_T_*x*_ and Ti_3_AlC_2_. SEM imaging of (e) Ti_2_NT_*x*_, (f) Ti_4_N_3_T_*x*_, (g) Ti_3_CNT_*x*_, (h) Ti_3_C_2_T_*x*_ MXene. The lateral size of the individual MXene flakes is roughly 5 μm. Raman spectra of delaminated (i) Ti_2_NT_*x*_, (j) Ti_4_N_3_T_*x*_, (k) Ti_3_CNT_*x*_, and (l) Ti_3_C_2_T_*x*_ MXenes. Raman spectroscopy was gathered using a 532 nm laser at 50% power at a 1 s exposure time for Ti_2_NT_*x*_ and Ti_4_N_3_T_*x*_ MXenes and a 633 nm laser for Ti_3_CNT_*x*_ and Ti_3_C_2_T_*x*_ MXenes.

The morphological changes after the etching were further understood by scanning electron microscopy (SEM) imaging of the delaminated samples (Figure 1e–h). After delamination in water, the MXenes exhibit a characteristic two-dimensional, flake-like morphology with lateral sizes on the order of several micrometers (∼5 μm). The flakes appear as thin, stacked sheets rather than bulk particles, indicating successful removal of the parent MAX phase. It should be noted that SEM primarily provides lateral and surface morphological information and does not allow unambiguous determination of the exact number of layers. Therefore, the observed contrast and edge features in Figure 1e–h are indicative of few-layer or thin multilayer MXene stacks, rather than strictly single-layer sheets. Alongside the visible 2D layered morphology, EDS further corroborates the successful synthesis of MXenes, which confirms the removal of A-site elements (Figures S1–S4).

The structural property of the synthesized materials was further scrutinized by investigating the vibrational modes of delaminated MXenes using Raman spectroscopy (Figure 1i–l). All MXenes clearly exhibit four broad Raman-active modes centered at ∼150 (ω1), 270 (ω3), 400 (ω5), and 600 (ω7) cm^–1^. This peak broadening stems from the structural distortion and local symmetry breaking relative to the parent *P*6_3_/*mmc* structure, induced by the electron density associated with surface termination groups.^30^ The E_1g_ (ω3) and A_1g_ (ω5) modes arise from the parallel and perpendicular vibrations of Ti atomic planes, and the broad nature of these modes can be attributed to the increased interlayer spacing and structural disorder introduced during the etching and delamination processes. Moreover, DFT Raman simulations indicate that these vibrational modes broaden and shift depending on surface termination coverage, which is consistent with the experimentally observed line widths.^31^ In particular, the dominant (ω5) mode observed across all samples arises from a coupled vibration of Ti atomic planes and surface terminations, with both in-plane and out-of-plane components contributing to its intensity. The vibrational mode at 600 cm^–1^ (ω7) can be assigned to an A_1g_ vibrational mode associated with surface terminations, which is in agreement with the group theory that places an A_1g_ (Ti, T_*x*_) at the high-frequency region at this specific location for MXenes.^32–37^ Its resemblance to the A_1g_ mode of rutile TiO_2_ reflects a similar local Ti–O coordination rather than the presence of bulk oxide phases. The presence of such Ti–O groups is attributed to the passivating surface termination layer on the MXenes. The low-frequency E_1g_ vibrational mode at 140 cm^–1^ (ω1) may arise from the in-plane shear vibrations involving Ti and N atoms.^38^ However, the position of this vibrational mode coincides with the E_g_ vibrational mode of anatase TiO_2_ (150 cm^–1^), which originates from symmetric stretching of O–Ti–O,^39^ as well as with vibrations associated with −O– termination groups in Ti-based MXenes that share a similar bonding environment. DFT calculations further indicate that low-frequency Raman modes in Ti-based MXenes are strongly influenced by surface terminations, with Ti–N lattice vibrations hybridizing with Ti–O/Ti–OH motions.^31^ Given that this vibrational mode is consistently observed in carbide and carbonitride MXenes, it is therefore reasonable to assign this mode primarily to Ti–O bonding. Additionally, two weak shoulders observed at ∼340 (ω4) and 550 (ω6) cm^–1^, especially in the MNenes (Figure 1i,j), may originate from the residual unetched MAX phases due to the complex nature of molten-salt fluoride etching and/or from mixed-termination-induced local vibrational modes. Although the Raman spectra of Ti_2_NT_*x*_, Ti_4_N_3_T_*x*_, Ti_3_CNT_*x*_, and Ti_3_C_2_T_*x*_ appear broadly similar, this resemblance is expected because all samples share Ti-based MXene slabs and mixed surface terminations. In the 100–800 cm^–1^ window, the Raman response is primarily governed by Ti-sublattice vibrations strongly coupled to T_*x*_ groups. In addition, termination heterogeneity, stacking disorder, and local symmetry breaking introduced during molten-salt etching and delamination substantially broaden these modes, which can mask subtle composition-dependent differences. Overall, the experimentally observed Raman features, including peak positions, broadening, and relative intensities, are in excellent agreement with DFT Raman simulations of mixed-termination MXenes^31^ and previous experimental reports,^29,37^ confirming that surface chemistry and local symmetry breaking dominate the vibrational response of delaminated MXenes.

### MXene Surface Characterization

Prior to experimentally investigating water dynamics in the MXenes, the surface chemistry and elemental composition of the delaminated samples were first analyzed by X-ray photoelectron spectroscopy (XPS) to determine the electronic properties and surface compositions (Figures 2 and S5–S8). The corresponding fitting results are summarized in Table S1. The Ti 2p spectra for all MXenes were deconvoluted into Ti 2p_3/2_ and Ti 2p_1/2_ spin–orbit doublets, revealing the coexistence of multiple titanium oxidation states that are characteristic of surface-terminated MXenes (Figure 2a–d).^40–44^ In carbide and carbonitride MXenes, the dominant Ti 2p components correspond to Ti^4+^ species, with Ti 2p_3/2_ binding energies centered at ∼458.7–459.0 eV and Ti 2p_1/2_ peaks at ∼464.5–464.8 eV, whereas nitride MXenes exhibit a higher relative contribution from Ti^3+^ states. This does not reflect intrinsic bulk stability differences between carbides and nitrides but rather differences in surface electronic structure and termination chemistry following aqueous etching and delamination. In particular, the presence of lattice nitrogen in MNenes stabilizes partially reduced Ti environments at the surface, leading to a higher Ti^3+^ fraction despite exposure to oxidative conditions. Moreover, the Ti^4+^ features are attributed to surface Ti–O bonding environments, arising from oxygen- and hydroxyl-containing terminations. Here, oxygen-containing terminations refer to discrete –O/–OH groups bound to exposed Ti sites, whereas TiO_2_-like motifs refer to more extended Ti–O_*x*_-rich surface environments formed by partial oxidation or reconstruction of the outermost Ti atoms. In MNenes, these Ti–O_*x*_-rich environments may act as a thin passivating surface motif that electronically stabilizes the underlying Ti–N framework, rather than a bulk crystalline TiO_2_ phase. In contrast, in carbide and carbonitride MXenes, oxygen species are more commonly present as mixed terminations rather than as a well-defined passivating oxide-like surface environment.^28,40,41^ Ti^3+^ species are observed at lower binding energies (∼456.7–457.9 eV), reflecting partially reduced Ti sites associated with the Ti–N or Ti–C lattice framework of the MXenes. A minor contribution from Ti^2+^ species (∼455.2–455.9 eV) is also detected in Ti_4_N_3_T_*x*_ and Ti_3_CNT_*x*_, which is commonly associated with Ti atoms bonded to surface termination groups such as –O, –OH, and –F.

**Figure 2:**
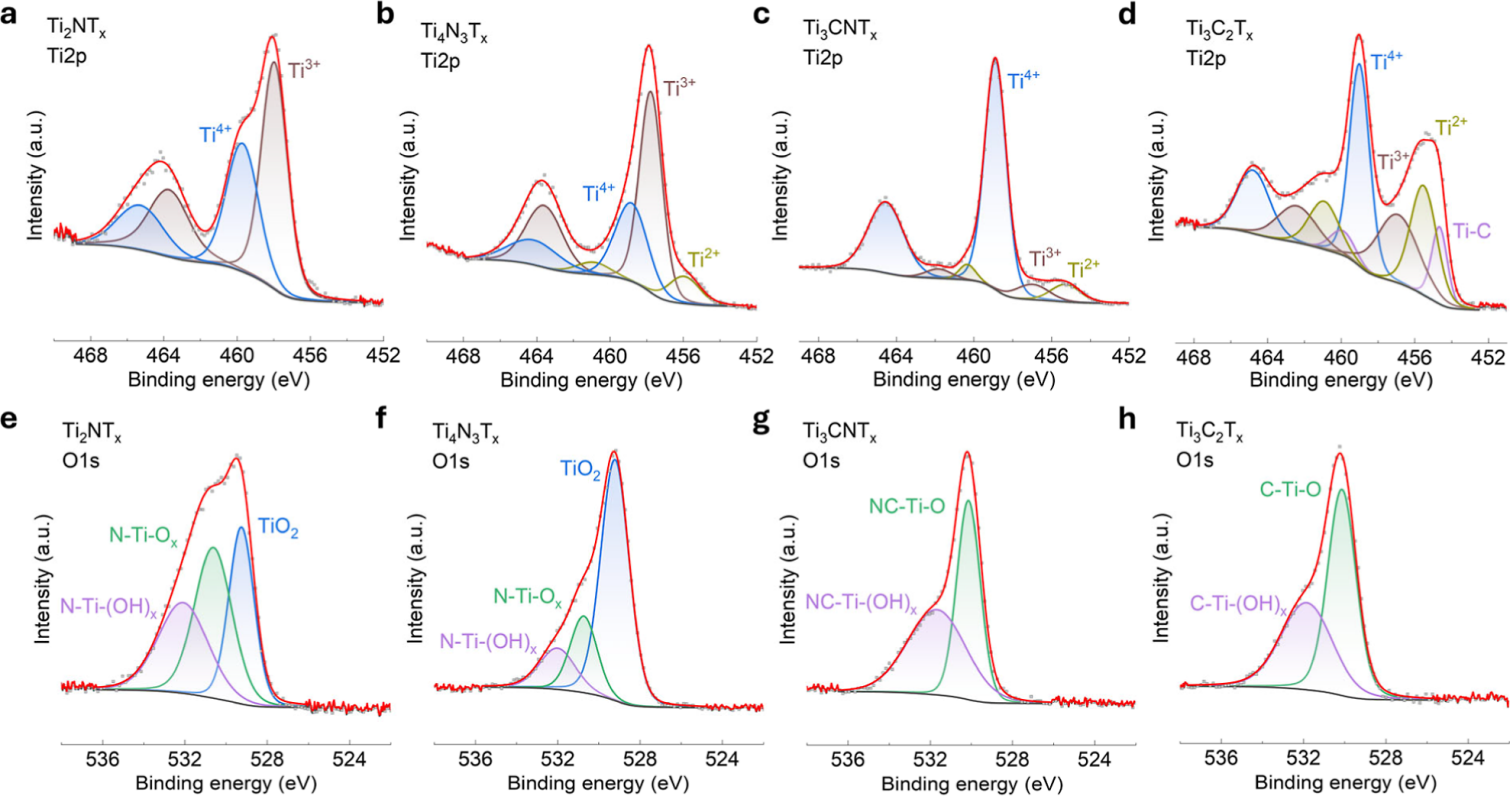
X-ray photoelectron spectroscopy (XPS) analysis of delaminated MXenes. (a–d) High-resolution Ti 2p spectra of Ti_2_NT_*x*_, Ti_4_N_3_T_*x*_, Ti_3_CNT_*x*_, and Ti_3_C_2_T_*x*_, respectively. (e–h) Corresponding high-resolution O 1s spectra. All spectra were acquired using a monochromatic Al Kα source (*h*ν = 1486.6 eV) under ultrahigh-vacuum conditions. Binding energies were calibrated using the adventitious C 1s peak at 284.8 eV. Background subtraction was performed using a Shirley background, and peak fitting was carried out using mixed Gaussian–Lorentzian (GL) line shapes.

The O 1s spectra further confirm the presence of multiple oxygen-containing chemical environments (Figure 2e–h). Peaks centered at ∼529.2 eV are assigned to TiO_2_-like oxygen species, while features at ∼530.1–530.7 eV correspond to surface Ti–O_*x*_ bonds. The TiO_2_-like oxygen species are only observed in MNenes, which is consistent with a passivating surface behavior, defined as the formation of an oxygen-rich interfacial layer that electronically stabilizes the underlying Ti–N lattice and suppresses further surface oxidation.^45,46^ This is further supported by the exclusive presence of TiO_2_-like O 1s components only in MNenes. Higher-binding-energy components at ∼531.7–532.1 eV are attributed to hydroxylated surface species (N/C–Ti-(OH)_*x*_), indicating that mixed –O/–OH terminations constitute the surface functional groups across all MXenes. The N 1s spectra reveal a primary component at ∼395.4–395.9 eV, which is assigned to N–Ti bonding within the nitride or carbonitride lattice (Figures S5–S7). The additional peak observed at the lower binding energy (394.6 eV) indicates the TiN_*x*_O_*y*_ phase formation, consistent with the –O termination groups.^45–47^ Additional N-related features at higher binding energies (∼397.6–399.7 eV) are attributed to surface-modified nitrogen species, including N–O_*x*_ environments and adsorbed nitrogen-containing species. For carbon-containing MXenes (Ti_3_CNT_*x*_ and Ti_3_C_2_T_*x*_), the C 1s spectra display characteristic contributions from C–Ti bonds at ∼ 281.1–281.4 eV, confirming the carbide or carbonitride framework (Figures S7 and S8).^28,43,44^ Additional components corresponding to C–Ti–O, C–C, C–O, and C=O species are observed at higher binding energies (284.8–288.7 eV), which are attributed to surface oxidation and adventitious carbon species commonly present on MXene surfaces. The presence of fluorine-containing termination groups is confirmed by the F 1s spectra (Figures S5–S8), which exhibit peaks at ∼683.4–684.7 eV associated with Ti–F_*x*_ or TiO_2‑*x*_F_*x*_ species. Minor signals corresponding to Al-containing fluoride species (Al­(OF)_*x*_ and AlF_3_) are also detected in select samples, indicating trace residual species from the fluoride etching process. Overall, these results demonstrate that the delaminated MXenes possess mixed surface terminations dominated by oxygen- and hydroxyl-containing groups, with smaller contributions from fluorine terminations. The coexistence of multiple Ti-oxidation states and diverse surface functional groups indicates the chemically rich nature of MXene surfaces.

### Probing Water Dynamics in MXenes by QENS Analysis

Now that we confirmed the successful synthesis, chemical, physical, and surface properties of the Ti_2_NT_*x*_, Ti_3_C_2_T_*x*_, Ti_4_N_3_T_*x*_, and Ti_3_CNT_*x*_ MXenes, we turned to QENS to investigate the interfacial water dynamics. QENS offers a macroscopic experimental “fingerprint” of the hydrogen dynamics averaged over the sample volume. It is important to note that QENS cannot spatially isolate the specific interlayer-surface water dynamics with the same angstrom-level resolution as the theoretical simulations; rather, the scattering signal is dominated by the more mobile water populations situated closer to the middle of the interlayer galleries. We began by collecting the energy-resolved “elastic” neutron scattering intensity of Ti_2_NT_*x*_, Ti_4_N_3_T_*x*_, Ti_3_CNT_*x*_, and Ti_3_C_2_T_*x*_ MXene samples to probe the temperature-dependent water dynamics (Figure 3a). The experimental details are provided in the Supporting Information. These scattering profiles, which serve as a diagnostic tool, reveal the temperature window in which the water molecular dynamics in the samples fall within the detection limit of the neutron spectrometer.^18^ Additionally, the scattering intensities at the lowest temperature (35 K in our case) correspond to the amounts of hydrogen-containing species, primarily adsorbed or intercalated water molecules, present in the samples. The mass-normalized elastic intensities (Figure 3a) show no abrupt drop with increasing temperature, indicating the absence of a bulk-like freezing–melting transition. This steady variation suggests that the water molecules are strongly confined within the MXene layers.^48^ However, changes in slope at higher temperatures are more pronounced in Ti_2_NT_*x*_ and Ti_4_N_3_T_*x*_ samples compared to Ti_3_CNT_*x*_ and Ti_3_C_2_T_*x*_ MXenes, suggesting greater water mobility in MNenes. Furthermore, the Ti_2_NT_*x*_ and Ti_4_N_3_T_*x*_ MXenes exhibit higher intensities at the baseline temperature, indicating that these samples contain more interlayer water than Ti_3_CNT_*x*_ and Ti_3_C_2_T_*x*_ MXenes. This is consistent with earlier reports that carbonitride MXenes exhibit nonmeasurable hydration levels due to the predominance of fluorine terminations and a more hydrophobic surface chemistry.^49^

**Figure 3:**
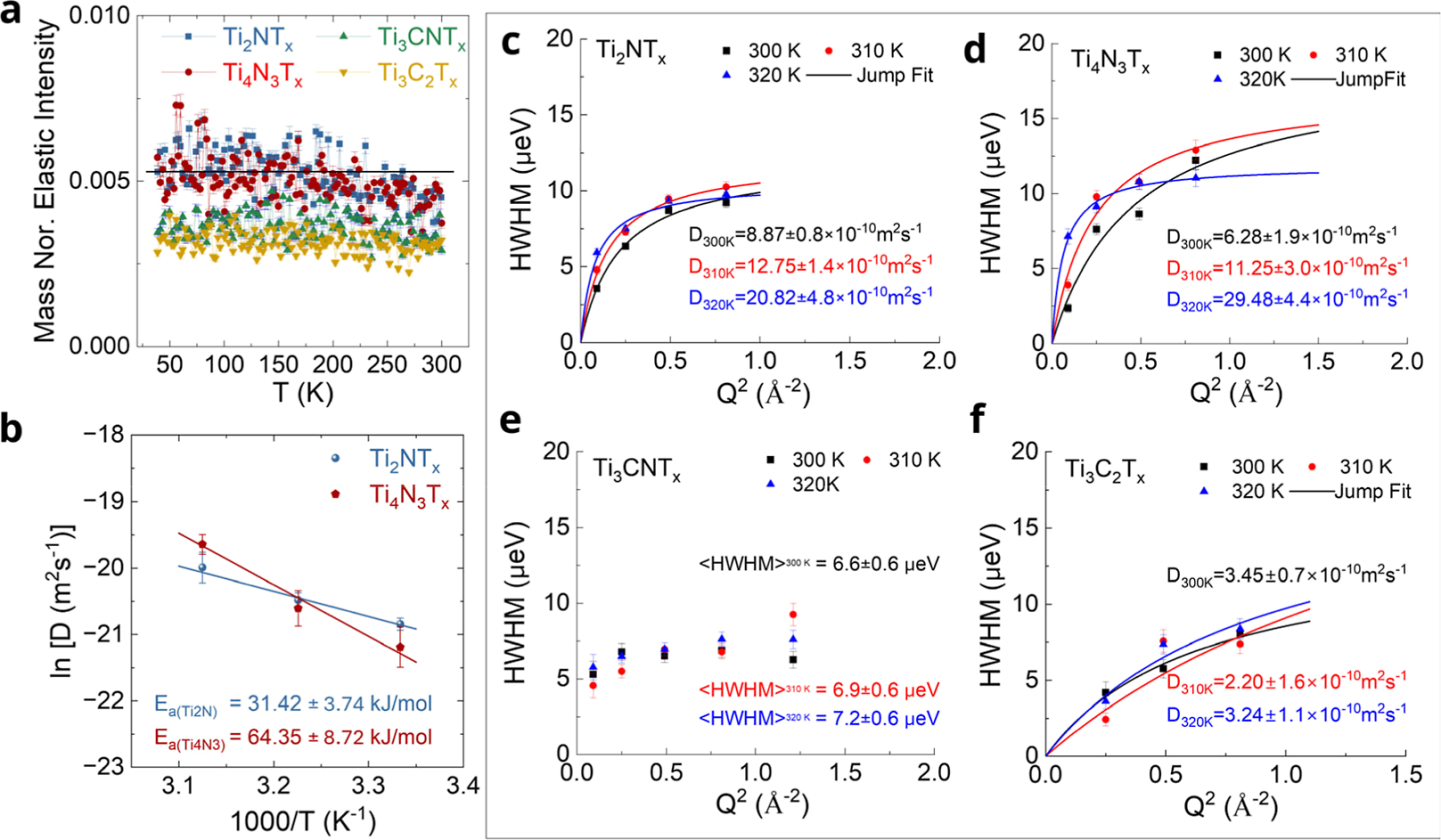
(a) Mass-normalized elastically scattered neutron intensities as a function of temperature at a representative *Q* = 0.7 Å^–1^. (b) Temperature dependence of the diffusion coefficients of Ti_2_NT_*x*_ compared to Ti_4_N_3_T_*x*_. The solid lines are the Arrhenius fit. *Q*-dependence of the HWHM of the QENS signals as a function of temperature. (c) Ti_2_NT_*x*_, (d) Ti_4_N_3_T_*x*_, (e) Ti_3_CNT_*x*_, and (f) Ti_3_C_2_T_*x*_ MXenes.

To directly quantify water mobility, QENS spectra were measured at 300, 310, and 320 K. The data were fitted using a single Lorentzian function to capture the quasi-elastic broadening at all measured temperatures, suggesting the presence of only one population of mobile water molecules in all samples across all temperatures. The model functions used for data analysis are summarized in eq 1 (Supporting Information), and the representative fits at *Q* = 0.7 Å^–1^ are shown in Figure S9. The half-width at half-maximum (HWHM) values of the QENS signals, extracted from the fits (Figure 3c–f), show different Q-dependence among the MXenes. For Ti_2_NT_*x*_ (Figure 3c) and Ti_4_N_3_T_*x*_ (Figure 3d), the HWHM approaches zero at *Q* = 0 and increases as *Q* increases, flattening at higher *Q* values. This behavior indicates characteristic long-range jump diffusion of water molecules. Moreover, this translational mobility of the water molecules directly correlates with the higher water content in these samples; the latter is evident from the elastic intensity profiles (Figure 3a). The derived self-diffusion coefficients of the water molecules in both MNene samples are comparable in magnitude (∼10^–10^ m^2^/s), with slightly higher values in Ti_2_NT_*x*_ compared to Ti_4_N_3_T_*x*_ at 300 K. Although the elastic intensity scan indicates a comparable amount of water in both samples, the higher mobility in Ti_2_NT_*x*_ suggests a less restricted interfacial environment for interlayer water compared to Ti_4_N_3_T_*x*_.

These self-diffusion values are slightly lower but within the same range as those reported for Ti_3_C_2_T_*x*_ MXene synthesized using 48% HF etching.^48^ The slightly lower self-diffusivity in the current samples, compared to the 48% HF-etched Ti_3_C_2_T_*x*_, may result from a synergistic effect of the synthesis conditions, which influence surface functional groups and the confinement geometry associated with interstack spacing. Nevertheless, this moderate reduction in diffusivity, compared to bulk water, indicates that the water molecules in the MNenes (Ti_2_NT_*x*_ and Ti_4_N_3_T_*x*_) occupy relatively loosely packed interstack gaps, as seen in pristine Ti_3_C_2_T_*x*_ MXenes.^48^ This also explains why the diffusivities in the measured samples do not simply scale with the interlayer *d*-spacing, as one could intuitively expect for the water molecules residing within the layers.

Upon increasing the temperature, both Ti_2_NT_*x*_ and Ti_4_N_3_T_*x*_ MXenes exhibit strong temperature dependence in diffusivity, following Arrhenius behavior as shown in Figure 3b, further supporting thermally activated translational diffusion. This pronounced temperature dependence again correlates with the relatively high water content, as confirmed by the elastic scans in Figure 3a. The extracted activation energy for water diffusion in Ti_4_N_3_T_*x*_ is approximately twice that of Ti_2_NT_*x*_, indicating stronger interactions between the water molecules and the Ti_4_N_3_T_*x*_ surface. This restricted mobility may stem from a higher density of hydrophilic surface terminations in Ti_4_N_3_T_*x*_.

In contrast, the HWHM of Ti_3_CNT_*x*_ MXenes (Figure 3e) is nearly *Q*-independent, indicating localized, nontranslational dynamics of the water molecules. Given the lower water content in Ti_3_CNT_*x*_, as shown in the elastic scan (Figure 3a), these water molecules are likely more tightly bound to the MXene surface, inhibiting long-range mobility. The average HWHM (≈6.6 μeV) remains within the experimental uncertainty across the studied temperature range (300–320 K), and the associated localized jump rate (relaxation time ∼100 ps) appears insensitive to temperature, consistent with the reported Ti_3_C_2_T_*x*_ MXene at low hydration levels.^50^ On the other hand, despite the similar mass-normalized elastic intensity indicating roughly the same amount of water in Ti_3_CNT_*x*_ and Ti_3_C_2_T_*x*_ (Figure 3a), the HWHM of Ti_3_C_2_T_*x*_ shows a strong *Q*-dependence, indicating that surface terminations, not water content alone, govern the water mobility (Figure 3f). Nevertheless, the diffusion coefficient is of the same order of magnitude but smaller compared to the MNenes at 300 K, suggesting that the water is more confined in Ti_3_C_2_T_*x*_ and, therefore, does not show a strong temperature dependence of diffusivity. The diffusivities are the same within the limits of the uncertainty of the measurements. Both carbide and nitride MAX phases show no detectable water mobility within the sensitivity limit of the instrument (the data can be fitted with a delta function alone, and no Lorentzian component could be accommodated, Figure S10), confirming that the observed dynamics arise uniquely from interstacks of MXene. Overall, for the delaminated MXenes, QENS reveals a clear material-dependent dynamic water mobility response. Water confined within Ti_2_NT_*x*_ and Ti_4_N_3_T_*x*_ exhibits long-range translational self-diffusion, whereas in Ti_3_CNT_*x*_ the water dynamics are predominantly localized. This distinct behavior of the interlayer water can be attributed to the differences in surface functional groups and the morphology of the individual MXene samples.^51^

### Interfacial Water Structure and Hydrogen Bonding Network

To establish an understanding of the water–MXene interface, the intrinsic structural and thermodynamic properties of four MXenes, Ti_2_NT_*x*_, Ti_3_C_2_T_*x*_, Ti_4_N_3_T_*x*_, and Ti_3_CNT_*x*_, were first determined using DFT. The formation energy and stability of functional groups on different MXene models were investigated by constructing 2 × 2 supercells of monolayer MXenes including various ratios of –F, –O, and –OH. The optimized bare and functional MXene top and side views of Ti_2_NT_*x*_, Ti_3_C_2_T_*x*_, Ti_4_N_3_T_*x*_, and Ti_3_CNT_*x*_ are shown in Figure S11, which serve as the starting point for all subsequent AIMD simulations. The formation energy (Δ*G*_f_) was computed as a function of surface termination composition (Figure S12), which reveals a consistent stability trend across all systems, where increasing –F and –O coverage monotonically lowers the formation energy, while –OH-rich surfaces are less favorable. The thermodynamic hierarchy (–F > –O > –OH) arises from the stronger surface Ti–F and Ti–O bonding relative to Ti–OH, whereas the ternary stability maps further confirm that fluoride- and oxide-dominated surfaces are energetically preferred (Figures 4a–c and S12). Based on our XPS analysis (Figure 2) and previous works, which showed mixed surface compositions for Ti_4_N_3_T_*x*_ ^40^ and Ti_2_NT_*x*_,^37,41^ as well as a higher fluorine (–F) coverage for Ti_3_CNT_*x*_,^28^ we modeled MXene surfaces with mixed functional groups that approximate our experimental findings and systematically varied the compositions to capture the effect of fluorine-rich and oxygen/hydroxyl-rich surface environments. Two distinct mixed-termination models were constructed for Ti_2_NT_*x*_ (F_0_O_25_OH_75_, F_25_O_25_OH_50_), Ti_3_C_2_T_*x*_ (F_0_O_25_OH_75_, F_25_O_25_OH_50_), Ti_3_CNT_*x*_ (F_25_O_25_OH_50_, F_50_O_25_OH_25_), and Ti_4_N_3_T_*x*_ (F_0_O_25_OH_75_, F_25_O_25_OH_50_), as illustrated in Figure S13.

**Figure 4:**
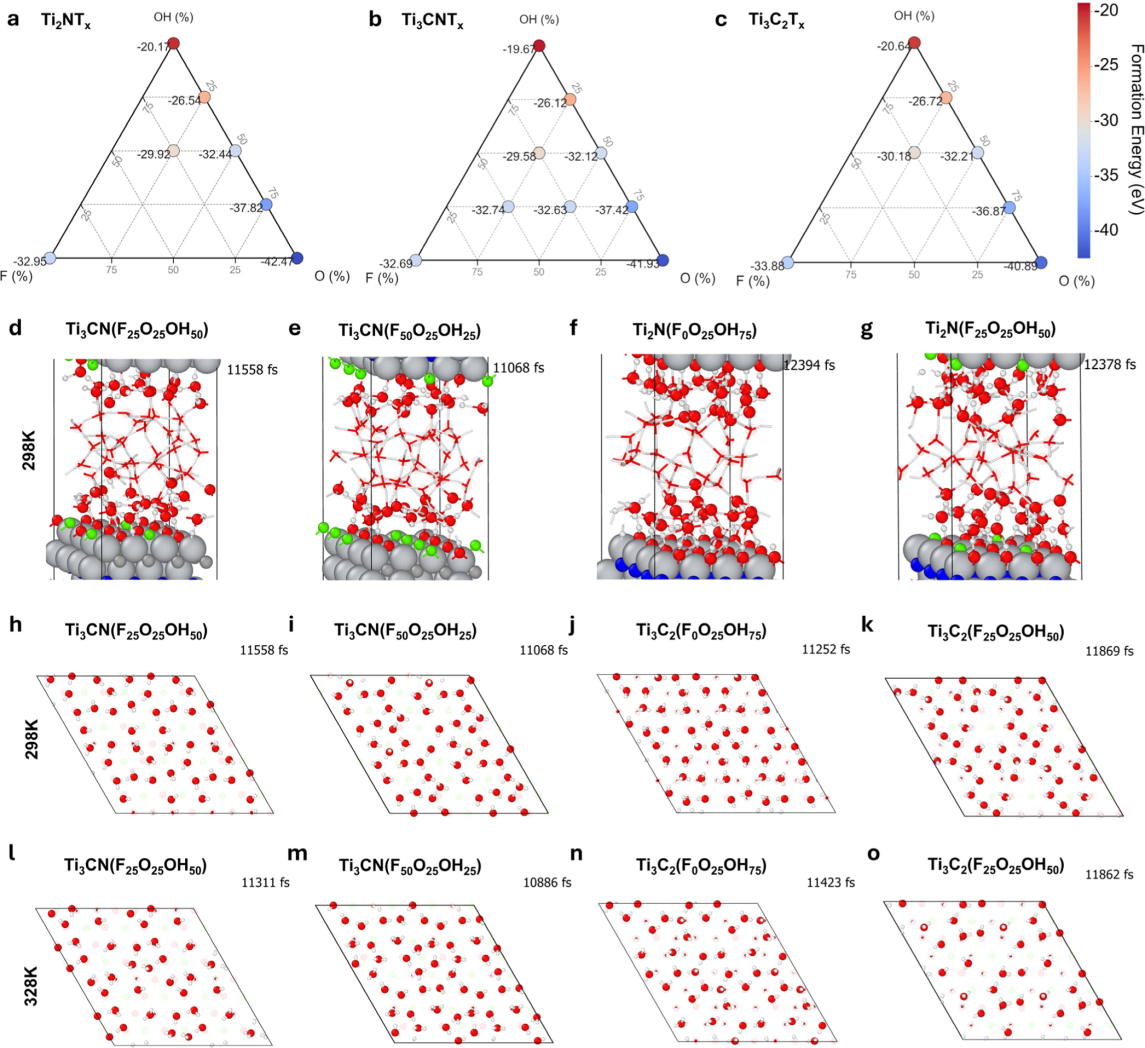
Stability and hydration structure of functionalized MXene surfaces. (a–c) Ternary diagrams showing the calculated formation energy (eV) relative to the bare surface for mixed-termination monolayers of (a) Ti_2_NT_*x*_ and (b) Ti_3_CNT_*x*_. (c) Ti_3_C_2_T_*x*_ with the varying compositions of –F, –O, and –OH groups. (d–g) Final configuration snapshots from AIMD simulations at 298 K, illustrating the side view of the water/MXene interface for (d) Ti_3_CNT_*x*_(F_25_O_25_OH_50_), (e) Ti_3_CNT_*x*_(F_50_O_25_OH_25_), (f) Ti_2_NT_*x*_(F_0_O_25_OH_75_), and (g) Ti_2_NT_*x*_(F_25_O_25_OH_50_), functionalized MXene surfaces at 298 K. (h–o) Top-down views of the first water layer structure from AIMD simulations on selected surfaces (h) Ti_3_CNT_*x*_(F_25_O_25_OH_50_), (i) Ti_3_CNT_*x*_(F_50_O_25_OH_25_), (j) Ti_3_C_2_T_*x*_(F_0_O_25_OH_75_), and (k) Ti_3_C_2_T_*x*_(F_25_O_25_OH_50_) at (h–k) 298 K and (l–o) 328 K.

AIMD snapshots of equilibrated configurations at 298 and 328 K (Figures S13 and S14) reveal that all functionalized MXene surfaces are highly hydrophilic, inducing a dense and ordered hydration layer that sharply contrasts with the less ordered nature of the bulk liquid water. The first interfacial layer often exhibits “ice-like” ordering stabilized by a continuous hydrogen-bonding network (Figure S15). Upon increasing the temperature, this network remains largely intact but becomes more dynamic and less crystalline (Figure S16), consistent with thermally activated mobility observed experimentally in QENS (Figure 3a). Deriving statistically converged macroscopic diffusion coefficients requires continuous simulation times in the order of 50–200 ps, which is currently beyond the reach of our AIMD setup, especially given the necessity of testing multiple termination systems. Because of this time scale limitation, we utilized hydrogen bonding lifetimes and time correlation functions as a rigorous, local proxy to estimate the interaction strength and relative mobility of the water layers. Therefore, AIMD provides insights into the *local* structural relaxation and binding lifetimes, which fundamentally underpin the macroscopic ensemble-averaged diffusion observed in QENS.

Interestingly, upon closer examination, a counterintuitive behavior emerges when comparing surfaces of varying termination chemistry. While –OH groups naturally promote hydrogen bonding, surfaces richer in –F, typically considered less hydrophilic, exhibit unexpectedly enhanced structural ordering in the first hydration layer, in which the water molecules are observed to “lock on” to nearby –OH groups, forming highly regular arrangements (Figure 4d,e,g) in contrast to the mobile case (Figure 4f). In this case, the F atoms act as passive constraints with their limited hydrogen-bonding capability, forcing water molecules to orient more uniformly around the remaining −OH and −O sites, leading to a low-entropy, quasi-crystalline arrangement. On the other hand, surfaces with abundant −OH groups provide multiple bonding options, introducing configurational freedom and higher dynamism (Figure 4e). These results highlight how mixed terminations collectively affect water structuring and dynamics in transition metal layered compounds.

Further insights into the observed structural templating are provided by top-down projections of the first hydration layer (Figures S15 and S16), which display polygonal ring motifs (4-, 5-, and 6-membered). The distribution of these motifs evolves with the surface termination chemistry. For instance, on the Ti_3_C_2_T_*x*_ surface at 298 K, increasing the –F concentration from 0% to 25% causes a shift in the dominant ring structures from 4- and 5-membered (Figure 4j) rings to 5- and 6-membered rings (Figure 4k). Conversely, a similar increase in –F content on the Ti_3_CNT_*x*_ surface leads to a reversion to a more grid-like pattern composed of 4- and 5-membered rings (Figure 4h,i). These results demonstrate that the local arrangement of interfacial water is a complex emergent property, dictated not by the surface termination or the MXene type alone, but by the specific combination of the two. Furthermore, by comparing the increase of temperature (Figure 4h–k versus Figure 4l–o), we further found out that the first water layer pattern changes significantly for the Ti_3_C_2_ surfaces with less –F_,_ but less change for Ti_3_CN with more –F, which further provides the evidence to support richer in –F could anchor the first water layer molecules and their water ring structures.

DFT analyses were done in MXene models with a 1 nm separation, whereas AIMD simulations were carried out using a larger 1.5 nm spacing to understand the multilayered structural dynamics of the water. We note that this 1.5 nm confinement distance sits at the upper bound of the experimental range of 0.68–1.43 nm and may exhibit slightly more bulk-like middle-layer characteristics than narrower experimental confinements. However, rather than solely investigating the first 1–2 layers of strongly bound interfacial water, we aimed to capture the structural transition of water across the entire interlayer, observing how the fluid behaves as it approaches bulk-like conditions.

### Quantitative Analysis of the Hydrogen Bond Network

Beyond visual inspection and rigorously quantifying the interfacial hydrogen bond (HB) network, we analyzed its density, composition, and spatial distribution. Quantitative analysis of the HB network (Figures S17–S20) confirms that the number and strength of interfacial HBs scale directly with the surface –OH concentration. Systems with higher hydroxyl coverage, such as Ti_2_NT_*x*_(F_0_O_25_OH_75_), sustain the largest HB densities at both 298 and 328 K. The temporal evolution of the total normalized number of hydrogen bonds (N_HB_) is presented in Figure 5a,b for medium-to-strong HBs (defined by a 2.9 Å O–O distance cutoff) and in Figure S17 for strong HBs (2.7 Å cutoff). A direct comparison between the two figures reveals that strong HBs constitute approximately 40–50% of the total medium-strong HB population. Furthermore, the temporal fluctuations in the total N_HB_ are primarily driven by the formation and breaking of these strong hydrogen bonds, while the number of medium to medium-strong bonds remains relatively stable. Moreover, with increasing temperature, the total number of strong HBs decreases slightly, reflecting enhanced molecular mobility but not structural breakdown. Deconvoluting the total HB population into slab–water and water–water components reveals a critical trade-off governed by surface composition (Figure 5c–f). Ti_3_CNT_*x*_, characterized by high –F coverage, exhibits the fewest slab–water HBs but compensates with a denser water–water HB network (Figure 5c–d). This shift from adhesive (surface-anchored slab-water network) to cohesive (water–water network-dominated) bonding implies that weakly interactive, fluorine-rich surfaces promote intralayer structuring and hinder interfacial diffusion. In contrast, –OH-rich Ti_2_NT_*x*_ and Ti_4_N_3_T_*x*_ favor strong surface adhesion and thermally activated translational motion.

**Figure 5:**
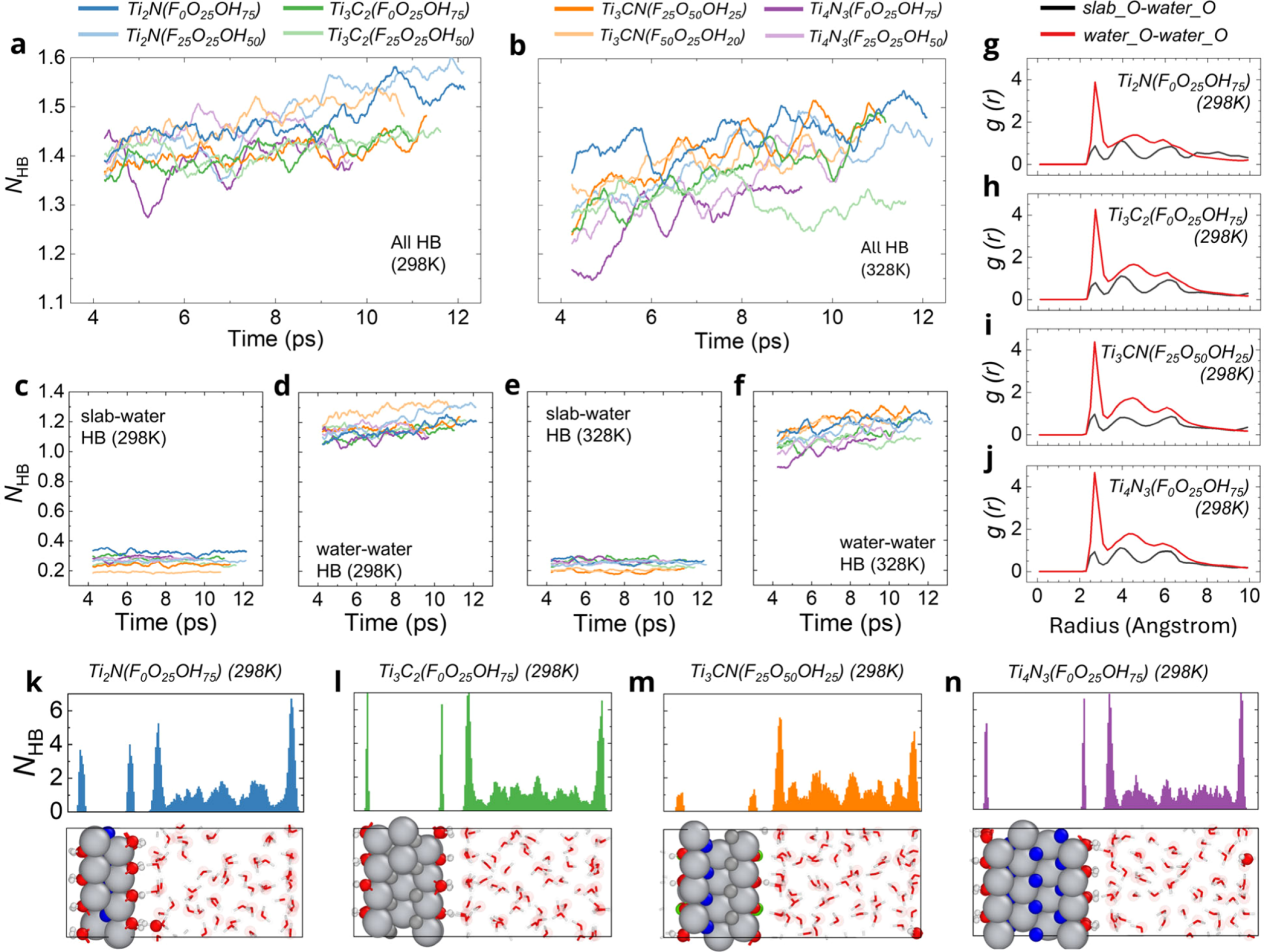
Dynamics and spatial distribution of HBs at MXene–water interfaces. (a–f) Time evolution of the normalized number of strong hydrogen bonds (N_HB_), defined by a distance cutoff of 2.9 Å, calculated using a 0.05 ps moving average. (a,b) Total system HBs, (c,e) slab–water HBs, and (d,f) water–water HBs at 298 and 328 K, respectively. (g–j) Radial distribution functions *g*(*r*) characterizing oxygen–oxygen pairwise interactions for solely water–water (blue) and solely between slab–water (orange) pairs at 298 K. (k–n) Spatial analysis of hydrogen bonding for average accumulation normalized hydrogen bonding results (4 ps to ∼10 ps) for (k) Ti_2_N­(F_0_O_25_OH_75_), (l) Ti_3_C_2_T_*x*_(F_25_O_50_OH_25_), (m) Ti_3_CN­(F_25_O_50_OH_25_), and (n) Ti_4_N_3_(F_0_O_25_OH_75_) MXene–water interfaces at 298 K and analysis with medium-strong to strong HB cutoff (2.9 Å). The bottom panels show representative simulation snapshots, while the top histograms illustrate the average accumulation of HBs as a function of the donor oxygen atom’s position (*z*) perpendicular to the MXene slab.

Our analysis focuses on the dominant H-bonding networks governed by O and OH terminations. When analyzing the AIMD trajectories, we observed that the water molecules generally maintain a larger spatial distance from the –F terminations, suggesting that the O–H···F interactions are relatively weak compared to the highly active O and OH sites. Consequently, we only included the H-bonds from the stronger OH and O interactions. While re-evaluating the entire dynamic data set to incorporate F-mediated bonding pathways exceeds our current computational capacities for this study, we acknowledge that transient O–H···F interactions may also contribute to the overall stabilization. Thus, our models represent idealized limits intended to isolate specific termination chemistry, whereas experimental hydration structures may inherently be more disordered due to lattice heterogeneity.

Spatially resolved HB distributions along the surface normal (Figures 5k–n, S13, and S20) further support the above interpretation. A pronounced N_HB_ appears within the first two Å adjacent to the MXene surface, confirming that most strong hydrogen bonds are localized at the immediate interface, involving either a surface –OH group acting as a donor or an oxygen atom in the first water layer acting as an acceptor. Very few strong HBs are observed between subsequent water layers, underscoring the highly localized nature of the surface’s templating influence. Interestingly, increasing the temperature broadens these peaks but does not eliminate them (Figures S19 and S20), reflecting the greater thermal motion and delocalization of the water molecules, yet the fundamental structure of a highly bonded interfacial layer persists. This effect is consistent with the thermally activated interfacial water mobility observed in QENS results (Figure 3). Overall, these results indicate that the strength and number of HBs at MXene–water interfaces depend strongly on surface terminations. Hydrophobic –F groups generally reduce MXene–water hydrogen bonding in Ti_2_NT_*x*_, Ti_3_C_2_T_*x*_, and Ti_4_N_3_T_*x*_ but enhance interfacial ordering in Ti_3_CNT_*x*_, where increasing surface fluorination yields the most stable and organized water layers (Figure 5k–n). Moreover, the Ti_3_CNT_*x*_ with 25% –F water layer structures remain when temperatures increase, consistent with *Q*-independent localized water dynamics observed in QENS (Figure 3e). More detailed discussion and quantitative analysis are provided in the Supporting Information.

### MXene Interlayer Water Structure and Orientation

Radial distribution functions (RDFs) were employed to reveal the finer details of water arrangement and average intermolecular distances within the MXene layers. The O–H RDFs (Figure S21) show sharp peaks near 1.8 Å for both slab–water and water–water pairs, typical of HBs. The O–O RDFs (Figures 5g–j and S22) feature first-peak positions at ∼2.7 Å for slab–water interactions, slightly shorter than the characteristic of bulk water (∼2.8 Å), indicating stronger and more constrained interfacial bonds. Interestingly, surfaces with reduced OH coverage display a narrower, more intense first peak in the slab–water RDF, suggesting that sparse –OH distributions favor well-defined, ordered hydration shells. On the other hand, temperature primarily affects the outer (second) coordination shell, leaving the primary interfacial HBs largely intact. For the O–O pair distance between water molecules, we observe that Ti_2_NT_*x*_ (F_0_O_25_OH_75_) shows a higher second-layer peak at 4.5 Å at higher temperatures. In contrast, Ti_3_C_2_T_*x*_ and Ti_3_CNT_*x*_ show minimal change with increasing temperature, which explains the temperature independence of diffusion coefficients observed in Figure 3e,f. As for Ti_4_N_3_T_*x*_ (F_0_O_25_OH_75_), its second peak shifts from 4.25 Å to 4.75 Å as the temperature increases (Figure S22d,l).

Likewise, angular distribution analyses (Figures S23–S26) reveal a clear orientational templating effect extending a few molecular layers from the surface, as illustrated in Figure 6a using Ti_3_CNT_*x*_ as the explanatory model. The H–H vector distributions peak at cos θ ≈ 0.2–0.4 (Figures 6d–e and S23), implying a consistent tilted orientation, whereas the O–H vector exhibits broad maxima near cos θ = 0 (Figures 6b–c and S24), corresponding to water molecules lying flat with their dipole moments nearly parallel to the surface to act as HB acceptors from the surface –OH groups. A smaller population shows peaks at cos­(θ) ≈ ± 1, indicative of molecules donating HBs either to the surface or toward the second layer. Layer-resolved analysis confirms that the first hydration layer is strongly oriented, with bimodal distributions centered around cos­(θ) ≈ ± 0.8, signifying a tightly locked configuration optimized for bonding with surface −O, −F, or −OH groups (Figures 6f–g and S25–S26). This high degree of alignment decays rapidly beyond the first layer, transitioning into the isotropic orientation characteristic of bulk water. With increasing temperature (328 K), these peaks broaden, indicating greater rotational freedom, while increased –F content flattens the orientation profile. In general, increasing either the temperature or the −F concentration promotes a flattened orientation for water molecules. This occurs because fewer flat-lying water molecules in the first layer allow the connected second layer to become more ordered (oriented up and down). Conversely, a highly oriented first water layer induces a more flattened orientation in the corresponding second layer. From the observations together, a slight change in a highly ordered first layer tends to induce a more flattened, disordered second layer, whereas a less ordered first layer can permit the second layer to adopt a more structured, up-and-down orientation. Interestingly, this explains the observed high activation energy in Ti_4_N_3_T_*x*_ compared to Ti_2_NT_*x*_ from QENS experiments (Figure 3b). While both systems retain a stable primary interfacial hydrogen-bond network, the enhanced termination density in Ti_4_N_3_T_*x*_ likely promotes stronger water binding and increased energetic barriers for translational motion, leading to a larger apparent activation energy for diffusion.

**Figure 6:**
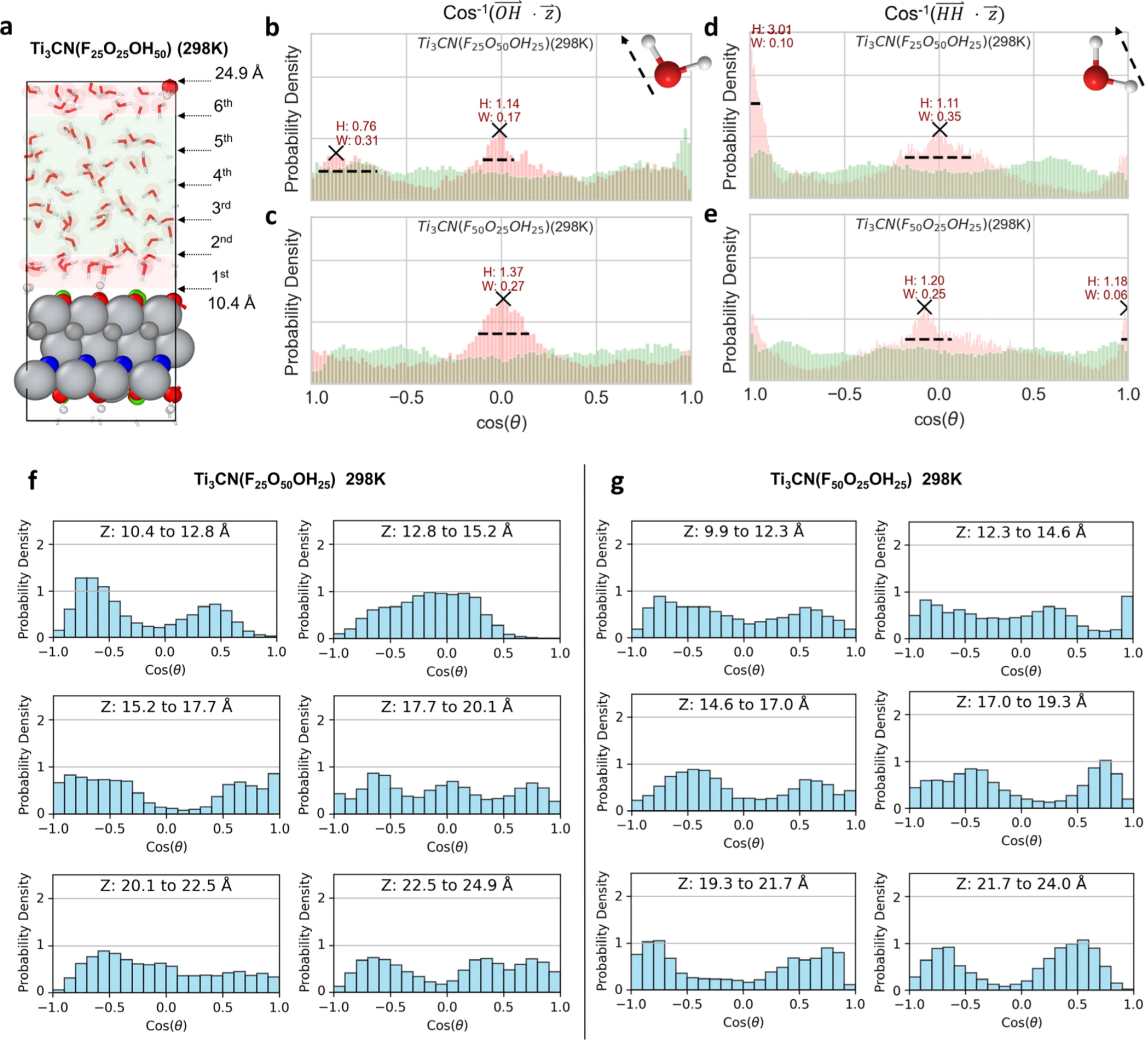
Interfacial water layering and orientational dynamics on functionalized Ti_3_CNT_*x*_ MXene surfaces at 298 K. (a) Illustration of the simulation cell for Ti_3_CN­(F_25_O_25_OH_50_), illustrating the definition of water layers along the *z*-axis. The vertical arrows delimit regions corresponding to the first through sixth hydration layers (spanning 10.4 Å to 24.9 Å), which serve as the sampling volumes for the analyses in panels b–g. (b–e) Probability density distributions of water molecule orientation in the first hydration layer (pink) compared to the background bulk water (green). The top row (b,d) corresponds to Ti_3_CN­(F_25_O_25_OH_50_), and the bottom row (c,e) to Ti_3_CN­(F_50_O_25_OH_25_). Panels b and c analyze the angle of the O–H bond vector relative to the surface normal (*z*-axis), while d and e analyze the H–H vector. Peak height (*H*) and full width at half-maximum (*W*) are provided to quantify the degree of ordering. (f,g) Spatial evolution of the water dipole orientation moving away from the surface. The histograms display the probability density of cos­(θ) (where θ is the angle between the water dipole vector and the *z*-axis) averaged over simulation times >4 ps. (f) Layer-by-layer analysis for the Ti_3_CN­(F_25_O_25_OH_50_) interface. (g) Layer-by-layer analysis for the Ti_3_CN­(F_50_O_25_OH_25_) interface. A value of cos θ = ± 1 indicates an orientation perpendicular to the surface, while cos­(θ) = 0 indicates a parallel orientation. Other surfaces are further compared in Figures S23–S26.

### Dynamics of the Interfacial Hydrogen Bond Network and Water Mobility

The final stage of our analysis shifts focus from the static structure to the time-dependent behavior of the interface. This study quantifies the stability and lifetimes of hydrogen bonds and the residence time of water molecules near the surface, culminating in a complete dynamic picture of the water–MXene interface. To quantify the persistence of hydrogen bonds, we calculated their time autocorrelation functions, *C*(τ). The results (Figures S27, S28, and S29) show that HBs between water molecules and the MXene surface persist far longer than those within the water network itself, confirming that surface sites act as deep potential wells that immobilize nearby molecules. A moderate increase in fluorine coverage from 0% to 25% tends to slow the decay of these correlations, producing longer-lived H-bonds.

For medium-strong HBs (2.9 Å cutoff), the role of fluorine in organizing the interfacial water layer is effective at 298 K but appears less effective at higher temperatures at 328 K (Figure S27a–h versus Figure S27i–p). At 298 K, the hydroxylated surface of Ti_4_N_3_T_*x*_ (F_0_O_25_OH_75_) initially exhibits a longer HB lifetime than its Ti_2_NT_*x*_ (Figure S27d,h,l,p) and Ti_3_C_2_T_*x*_ counterparts (Figure S27b,f,j,n), which diminishes upon fluorination or heating, consistent with the diffusion coefficient trends observed in Figure 3 with increasing temperature. In contrast, Ti_3_CNT_*x*_ exhibits an exceptional temperature resilience even at elevated temperatures; its medium-strong HB lifetime remains nearly unchanged (Figure S27c,g,k,o). This exceptional stability indicates that a sufficient concentration of fluorine on the Ti_3_CN surface can effectively push the first water layer to nearby –OH termination, creating an interfacial water network structure that is uniquely resistant to temperature fluctuations.

In the strong HB regime (2.7 Å cutoff), a clear substrate-dependent hierarchy can be observed (Figures S29 and S28a–d). For ACF at a fixed time lag of τ = 0.3 ps (Figure S28a), Ti_3_C_2_T_*x*_ initially forms the most stable H-bonds (Ti_3_C_2_T_*x*_ (0.280) > Ti_2_NT_*x*_ (0.222) > Ti_4_N_3_T_*x*_ (0.178)), but just 25% fluorination inverts this order (Ti_4_N_3_T_*x*_ (0.182) > Ti_2_NT_*x*_ (0.165) > Ti_3_C_2_T_*x*_ (0.128)), indicating that the influence of functional groups is not intrinsic but electronically coupled to the underlying MXene lattice. Charge-withdrawing –F alters the local polarization of adjacent –O and –OH sites differently on carbides versus nitrides, thereby modulating the binding energetics of the interfacial water. This demonstrates a synergistic or, in some cases, antagonistic coupling between the substrate electronics and the surface chemistry that ultimately governs the dynamics of the interfacial water. Overall, as expected, increasing temperatures generally decrease hydrogen bond stability. In contrast to the typical thermal degradation of hydrogen bonds, the strong HBs (2.7 Å cutoff) on the high-fluorine Ti_3_CNT_*x*_ surface are insensitive to temperature changes, and even show slower decay than at lower temperature, suggesting that high fluorine coverage indirectly “pins” the interfacial water to nearby –OH. This electronic stabilization allows the Ti_3_CNT_*x*_ surface to maintain a rigid first hydration layer even under higher thermal conditions that would typically disrupt the water–MXene interaction.

### Layer-Resolved Hydrogen-Bond Dynamics

To add spatial granularity to the dynamic analysis, HB lifetimes were calculated separately for water molecules within distinct regions: exclusively within the first water layer (w_layer11), between the first and second layers (w_layer12), exclusively within the second layer (w_layer22), and in the quasi-bulk region (w_layer_middle), as illustrated in Figure 7i. The results are shown for medium-strong HBs (<2.9 Å) in Figures 7a–d and S30 (continuous) and Figure S31 (intermittent) and for strong HBs (<2.7 Å) in Figures 7e–h and S32 (continuous) and Figure S33 (intermittent) and summarized their autocorrelation function (ACF) values specifically at a lag time of τ = 0.1 ps at Figure 7j. The first hydration layer (w_layer11) exhibits the slowest decay, confirming it behaves as a quasi-rigid, ice-like shell directly templated by the surface functional groups. In contrast, the interface between the first and second layers (w_layer12) represents a dynamically fragile zone where bond rearrangements occur rapidly, producing the shortest lifetimes. This interlayer region represents a zone of structural mismatch between the highly ordered, semisolid first layer and the more disordered, liquid-like second layer, making the formation of stable, long-lived hydrogen bonds difficult. Therefore, this interface between MXene hydration shells and the second layer is the primary point of dynamic failure in the interfacial structure that is highly susceptible to thermal energy. As for Ti_3_C_2_T_*x*_ (Figure 7b,f) and Ti_4_N_3_T_*x*_ (Figure 7d,h) systems with high −OH content, increasing the temperature from 298 to 328 K significantly shortens the HB lifetime in the second layer while leaving the first layer’s lifetime largely unaffected. This effect is even more pronounced for Ti_2_NT_*x*_ (F_0_O_25_OH_75_) (Figure S31), where the increased mobility of the second layer at 328 K could exert a relative shear force from thermal fluctuations on the first layer, causing the HB lifetime between the first and second layer (w_layer12) to decay more rapidly. The overall dynamic stability of the interfacial region is therefore not merely a function of the slab–water bond strength but is critically dependent on the mechanical and dynamic coupling between the first two hydration shells. Nitride-based MXenes display a more pronounced coupling between these two layers, suggesting that thermal motion in the outer water can shear or destabilize the first-layer network, while the carbides and carbonitrides maintain a clearer separation between solid-like and liquid-like domains.

**Figure 7:**
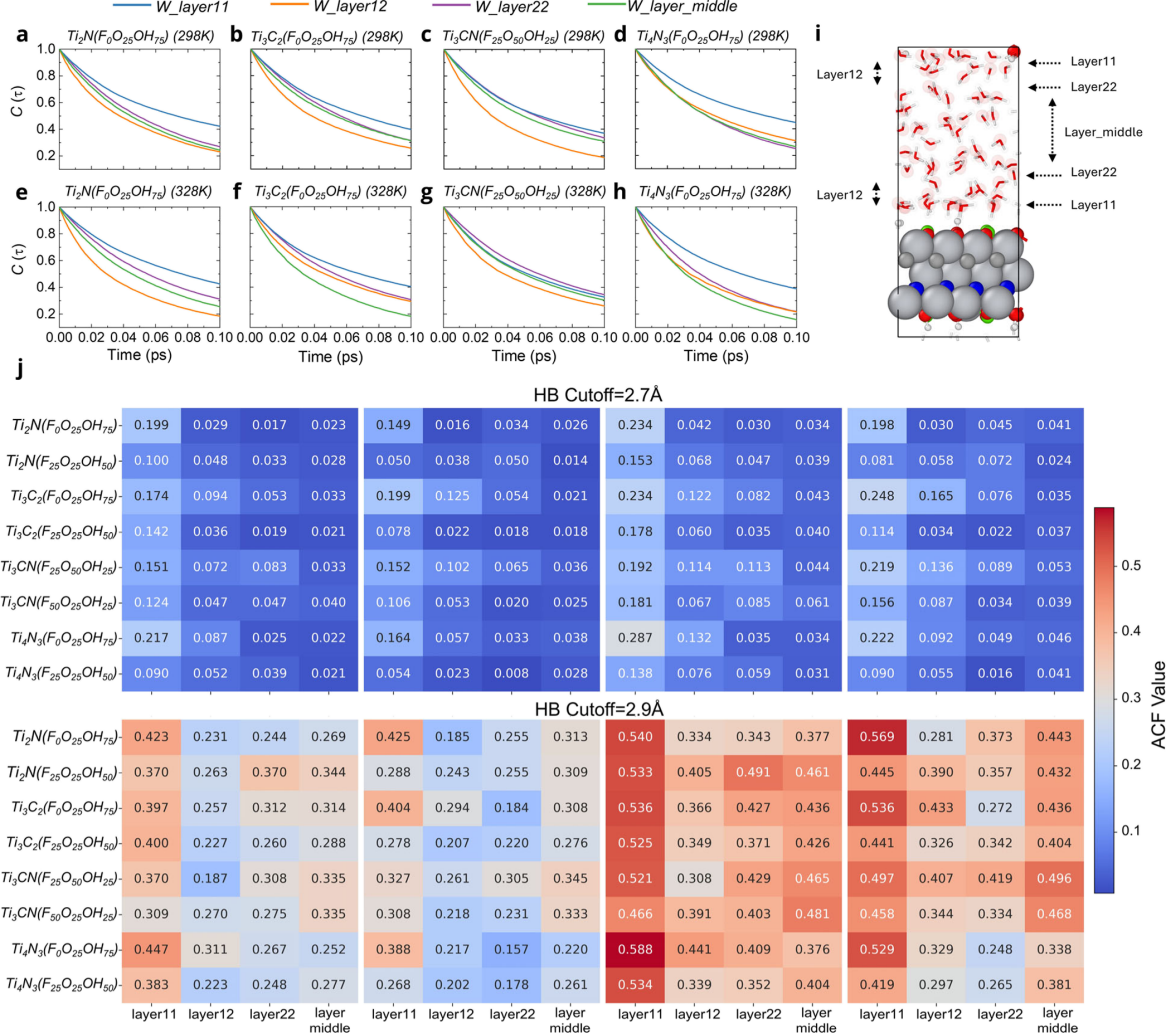
Analysis of layer-resolved hydrogen bond (HB) dynamics at functionalized MXene–water interfaces. (a–h) Continuous time autocorrelation functions (*C*_I_(τ) = 1), for hydrogen bonds using a distance cutoff of 2.9 Å at 298 K (a–d) and 328 K (e–h) on (a,e) Ti_2_N­(F_0_O_25_OH_75_), (b,f) Ti_3_C_2_(F_0_O_25_OH_75_), (c,g) Ti_3_CN­(F_25_O_50_OH_25_), and (d,h) Ti_4_N_3_(F_0_O_25_OH_75_). (i) Schematic illustration defining the spatial water regions relative to the surface: bonds formed exclusively within the first layer (layer11), between the first and second layers (layer12), exclusively within the second layer (layer22), and in the interlayer middle region (layer_middle). (j) Heatmaps of HB lifetime autocorrelation function (ACF) values at a lag time τ = 0.1 ps. The data is organized by HB distance cutoff (upper block: 2.7 Å; lower block: 2.9 Å) and lifetime definition (left columns: continuous, *C*_I_(τ) = 1; right columns: intermittent, allowing transient breaks, *C*_I_(τ) = 10). The color gradient indicates the decay rate, with red representing longer-lived HBs and blue representing shorter-lived HBs. Interestingly, the Ti_3_CN (with a 2.9 Å cutoff, 298 K, *C*_I_(τ) = 10) shows a relatively shorter layer11 lifetime than other systems, which indicates that due to formation of strong interactions between slabs, the lifetime of the first water layer itself decreases. Besides, layer_middle of Ti_3_CN shows a higher lifetime than all other systems, which indicates that Ti_3_CN with higher fluorine could promote water forming hydrogen bonds with itself. Besides, Row 7 is unique because it maintains a high **Layer 12** value. This suggests that the Ti_4_N_3_(F_0_O_25_OH_75_) lattice spacing or electronic structure, combined with –OH, creates a hydrogen-bonding network promoting a 1-water-layer morphology prone to form HBs with the second water layer.

### Water Residence Time at MXene Interfaces

As a final holistic measure of the overall strength of the water-surface interaction, we calculated the survival probability (SP) of water molecules in the vicinity of the MXene surface (within 2.5 Å upward and downward of slabs). The SP, as shown in Figure 8, represents the fraction of water molecules that start in this region and remain there continuously over time. A slower decay of the SP curve corresponds to a longer residence time and stronger water retention. All MXenes examined are strongly hydrophilic, retaining over half of their initial interfacial water layer throughout the simulation. However, their residence behavior varies dramatically for Ti_3_CNT_*x*_. Despite having fewer hydroxyl groups, Ti_3_CNT_*x*_ exhibits the slowest decay and hence the longest residence time, indicating that fewer but stronger H-bonds lead to a more stable and less mobile interfacial layer (Figure 8c,k). In contrast, Ti_2_NT_*x*_ and Ti_4_N_3_T_*x*_, while hosting a denser interfacial hydrogen-bond network, exhibit faster SP decay, indicating weaker individual water–surface interactions and a greater propensity for thermally activated water exchange at the interface. These results align directly with the QENS observation of localized hydrogen motion in Ti_3_CNT_*x*_, whereas Ti_2_NT_*x*_ and Ti_4_N_3_T_*x*_, possessing denser but weaker networks, allow thermally activated diffusion.

**Figure 8:**
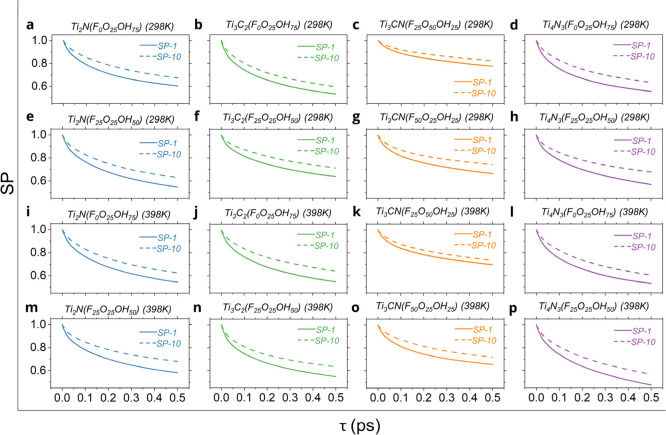
Survival probability (SP) of water molecules near various MXene surfaces (2.5 Å upward and downward from the MXene slab) at 298 K (upper subplots, (a–h)) and 328 K (lower subplots, (i–p)) across eight different MXene compositions. The plots compare two different intermittency intervals for SP: SP-1 (solid lines) and SP-10 (dashed lines) for different terminated MXenes (a,e,i,m) Ti_2_NT_*x*_, (b,f,j,n) Ti_3_C_2_T_*x*_, (c,g,k,o) Ti_3_CNT_*x*_, and (d,h,l,p) Ti_4_N_3_T_*x*_. These represent the probability that a water molecule located in a region at initial time *t*_0_ remains in that region at *t*_0_ + 10. The survival probability is defined as the fraction of water molecules that, having started within a specific region at time *t* = 0, remain continuously within that region up to a later time τ. A slower decay of the SP curve corresponds to a longer residence or permanence time of water molecules near the surface, implying stronger interactions. All the analyzed functionalized MXenes are strongly hydrophilic, keeping more than half of their initial water layer stable and staying close to the surfaces. Unexpectedly, Ti_3_CNT_*x*_, which has fewer hydroxyl (−OH) groups, showed the best water retention ability of all the MXenes at both 298 and 328 K. This suggests that even though a surface with fewer hydroxyl (−OH) groups has a lower total number of hydrogen bonds between water and the slab, reducing the −OH groups strengthens the individual HBs with the MXene surface. This stronger bonding creates a more stable and less mobile initial water layer that is less susceptible to temperature changes. In other words, having fewer but stronger hydrogen bonds creates a more rigid and temperature-resistant first water layer.

When considering the effect of temperature, the most common expectation is that as temperature increases, water molecules should gain more thermal energy and escape the surface more easily, leading to a lower survival probability. Interestingly, certain compositions display a counterintuitive temperature response. For instance, Ti_3_C_2_T_*x*_(F_0_O_25_OH_75_) (Figure S15b,j) and Ti_2_NT_*x*_(F_25_O_25_OH_50_) (Figure S15e,m) show increased water retention at 328 K. This behavior suggests that moderate heating can promote rearrangement into more energetically favorable bonding configurations, similar to a fragile-to-strong transition observed in supercooled or glassy water.^52^ More detailed discussion is provided in the Supporting Information. This also implies that interfacial water in confined MXene environments can deviate markedly from bulk thermodynamics, with surface chemistry dictating whether temperature enhances or disrupts stability. Overall, a surface with a high density of weaker H-bonds (e.g., high −OH) may allow for easy hopping and thus a shorter residence time, whereas a surface with fewer, but stronger and more localized, H-bonds can lead to a more stable, less mobile water layer with a longer residence time.

## Summary

In summary, we provide mechanistic, atomistic-level resolved insights into water dynamics confined within layered transition-metal carbides, nitrides, and carbonitrides by systematically integrating QENS with AIMD simulations. AIMD reveals that interlayer water forms a strongly templated first hydration layer at the metal–(C/N) surface, characterized by highly oriented molecules and long-lived hydrogen bonds, while the second hydration layer remains dynamically fragile and governs the onset of long-range translational motion. Quantitative analysis of hydrogen-bond density, lifetimes, residence times, and orientational distributions demonstrates that lattice light-element chemistry (C vs N) and surface termination composition jointly control the balance between surface water and water–water hydrogen bonding. Nitride-rich lattices (Ti_2_NT_*x*_ and Ti_4_N_3_T_*x*_) favor dense but comparatively weaker surface water hydrogen-bond networks dominated by –O/–OH terminations, resulting in thermally activated exchange between the first and second hydration layers and enabling long-range translational diffusion observed by QENS. In contrast, mixed C/N lattices (Ti_3_CNT_*x*_) stabilize a quasi-rigid interfacial water network in which fluorine-rich environments constrain the first hydration layer by anchoring water molecules to nearby –OH sites, producing long hydrogen-bond lifetimes, extended surface residence times, and temperature-insensitive, localized dynamics. Fully carbide systems (Ti_3_C_2_T_*x*_) exhibit intermediate behavior, highlighting that water mobility is not controlled only by the hydration level or interlayer spacing but by the coupling between the lattice electronic structure and termination-dependent interfacial bonding. Overall, this study provides a quantitative framework for understanding and tuning solid–liquid interfaces in layered transition-metal materials, with direct implications for electrochemical energy storage, catalysis, and ion-transport processes. While our simulations isolate the specific influences of C/N lattice chemistry and idealized surface terminations on water mobility, we note that the macroscopic dynamics observed experimentally are intrinsically convoluted with the material’s synthesis history, including the presence of residual salts, structural defects, and variable stacking morphology.

## Supplementary Material





## Notes

AIMD data (ZIP)
Computational details; EDS and XPS characterization; QENS spectra and fitting analysis; optimized MXene structures and formation-energy calculations; AIMD snapshots; interfacial water structure; hydrogen-bond network analysis; MXene interlayer water structure and orientation; hydrogen-bond lifetimes; layer-resolved hydrogen-bond dynamics; and water residence-time analysis (PDF)
