# Supplementary material for: Interfacial Hydrogen-Bond Dynamics in Transition Metal Compounds
Source: Chem Mater. 2026 Jul 25;38(15):7965–81. doi: 10.1021/acs.chemmater.6c01283 (PMC13471723; doi:10.1021/acs.chemmater.6c01283)
Supplement: Supplementary file 1 [file cm6c01283_si_002.pdf]

Supporting Information

Interfacial Hydrogen-Bond Dynamics in Transition Metal Compounds

David Kumar Yesudoss[1#], Hao-En Lai[1#], Naresh C. Osti[2], Bright Ngozichukwu[1], Joshua Ferguson[1], Eugene Mamontov[2], Perla B. Balbuena[1,3*], Abdoulaye Djire[1,4*]

[1]Artie McFerrin Department of Chemical Engineering, Texas A&M University, College Station, TX 77843, USA

[2]Neutron Scattering Division, Oak Ridge National Laboratory, Oak Ridge, TN 37831, USA

[3]Department of Chemistry, Texas A&M University, College Station, TX 77843, USA

[4]Department of Materials Science and Engineering, Texas A&M University, College Station, TX 77843, USA

Corresponding Authors: balbuena@tamu.edu; adjire@tamu.edu

# Indicates co-first author

Table of Contents

I. Computational details... 3
II. Experimental data… 6
III. Discussion 1. Energetic Stability and Structural Configurations of MXene Systems...... 13
IV. Discussion 2. Interfacial Water Structure and Hydrogen Bonding Network....................15
V. Discussion 3. Quantitative Analysis of the Hydrogen Bond Network 18
VI. Discussion 4. MXene Interlayer Water Structure 24
VII. Discussion 5. MXene Interlayer Water Orientation. 27
VIII. Discussion 6. Hydrogen Bond Lifetimes and Stability…..33
IX. Discussion 7. Layer-Resolved Hydrogen Bond Dynamics...38
X. Discussion 8. Water Residence Time at MXene Interfaces..42
XI. References...44

I. Computational Details

Geometry optimizations of slab

Optimized geometries were determined using the Vienna Ab initio Simulation Package (VASP)[1]. These calculations employed the Perdew–Burke–Ernzerhof (PBE) functional[2] within the generalized gradient approximation (GGA) and projector augmented wave (PAW)[3] pseudopotentials. The initial structures of slab systems were based on a reoptimized supercell from our prior works[4-6]. A 10 Å vacuum layer was added along the MXene edge direction to isolate the cleaved surfaces to prevent interactions between periodic images of slab. Geometry optimization was performed for 2 × 2 supercells. This procedure of structural optimization utilized the Monkhorst–Pack method[7] for Brillouin zone integration, with k-point meshes of 6 × 6 × 1. A cutoff energy of 500 eV was applied, with convergence tolerances of 0.01 eV/Å for forces and 10^{-6} eV for energy. Grimme's D3 method[8] and spin polarization were included. For degree of freedom. we fixed slab cell total volume, and allow All atomic positions, lattice parameters were fully relaxed while using the conjugate gradient method to obtain relax MXene slab structures.

AIMD simulation Setup

Ab Initio Molecular Dynamics (AIMD) simulations were performed to study hydrogen bonding change, hydrogen bonding strength, total hydrogen bonding number, water orientation and possibly diffusion of water change due to mainly three aspects: MXene material, MXene termination group composition ratio difference and temperature. AIMD Simulations were run in the NVT ensemble at 298 K and 328K using a Nosé thermostat (Nosé-mass = 0.5) and a 1 fs time step, for a total duration of 9.5-12 ps per simulation depending on the system sizes. We used a cutoff energy of 450 eV and set force and energy convergence tolerances to 10^{-5} eV with Grimme's D3 method and spin polarization were included. During the MD simulation, all atoms moved freely in all directions to correctly capture the vibration and the interaction between water molecules with MXene surface.

Overall, we study four total MXenes, including Ti_2NT_x, $Ti_3C_2T_x$, $Ti_4N_3T_x$ and Ti_3CNT_x with different surface termination group at 298 K and 328K. For Ti_2NT_x, $Ti_3C_2T_x$, $Ti_4N_3T_x$, previous experiment show a lower concentration of fluorine termination group on the surface, so we choose two lower fluorine cases $F_0O_{25}OH_{75}$, $F_{25}O_{25}OH_{50}$ to explore. As for Ti_3CNT_x, a higher fluorine

termination group was observed in experiment, so we chose two higher fluorine cases $F_{25}O_{50}OH_{25}$, $F_{50}O_{25}OH_{25}$ to explore how higher fluorine termination would influence the water dynamic of the system. To construct the simulation models, fully relaxed MXene slabs were solvated with water molecules using the amorphous packing tool in Materials Studio[9]. A 15 Å water interlayer was created above the monolayer, with the total number of water molecules calculated based on the bulk water density at 298 K (0.997 g cm^{-3}) and 328 K (0.9857 g cm^{-3}). This initial amorphous packing generated via Materials Studio relies on classical force fields, which are not ideal for evaluating equilibrium water structures. Because of this, the first several hundred femtoseconds of the AIMD simulations serve purely to relax the water molecules, allowing the system to shed its initial force-field-biased geometry and form equilibrium hydrogen-bonding networks driven by ab initio forces. Because the initial several picoseconds are excluded from our hydrogen bond correlation function calculations, the initial packing environment does not significantly bias the final equilibrium dynamics.

Hydrogen Bond autocorrelation and Lifetime

The dynamics of hydrogen bonding were investigated by calculating hydrogen bond time autocorrelation functions from the molecular dynamics (MD) trajectories. A hydrogen bond was considered to be formed if it satisfied specific geometric criteria for both distance and angle. A hydrogen bond was defined using the general formula D-H···A, where D-H is the donor group and A is the acceptor atom. To probe different interaction strengths, two separate distance cutoffs were applied for the donor-acceptor (D···A) separation: 2.7 Å to identify strong hydrogen bonds and 2.9 Å to also include those of intermediate strength. Concurrently, a strict angular criterion was enforced, requiring the donor-hydrogen-acceptor (D-H···A) angle to be greater than 150°. This angular constraint ensures that our analysis selects stable, linear hydrogen bonds, enabling a clear comparison of their respective lifetimes. Based on the system's topology, hydrogen atoms covalently bonded to oxygen were identified as donors. As our MXene surfaces lack other electronegative exposed elements like nitrogen, all oxygen atoms were treated as the sole potential acceptors.

We utilized the MDanalysis package[10, 11] and Water dynamics analysis module[12] to analysis calculated two distinct types of time autocorrelation functions to characterize the bond lifetimes: continuous autocorrelation function $C_C(t)$ and intermittent autocorrelation function $C_I(t)$. The

continuous autocorrelation function, $C_C(t)$, measures the probability that a specific hydrogen bond, present at a given starting point time t_0, remains continuously bonded until time t. This metric provides the lifetime of a single, uninterrupted bond based on geometric criteria, as it does not count the bond as present if it breaks and later reforms. The lifetime of these bonds is monitored as autocorrelation function as below;

$$C_C(t) = \left\langle \frac{h_{ij}(t_0)h_{ij}(t_0+\tau)}{h_{ij}(t_0)^2} \right\rangle \quad \text{(Equation 1)}$$

Where h_{ij} (t_0) =1 or h_{ij} (t_0) = 0 determined by whether the hydrogen bonding pair ij meet the geometric criteria at given t_0. And h_{ij} (t0+τ)=1 indicate this pair ij remain hydrogen bonded throughout the period t_0 to $t_0+\tau$. Angular brackets represent an expectation value over different starting point t_0 in given trajectory.

Conversely, the intermittent autocorrelation function, $C_I(t)$, calculates the probability that a hydrogen bond exists at an initial time t_0 and is also present at a later time t, regardless of the bond's state in the interim. This function allows for transient breaking and reformation, thereby reflecting the overall probability that a given donor-acceptor pair remains associated over time.

II. Experimental data

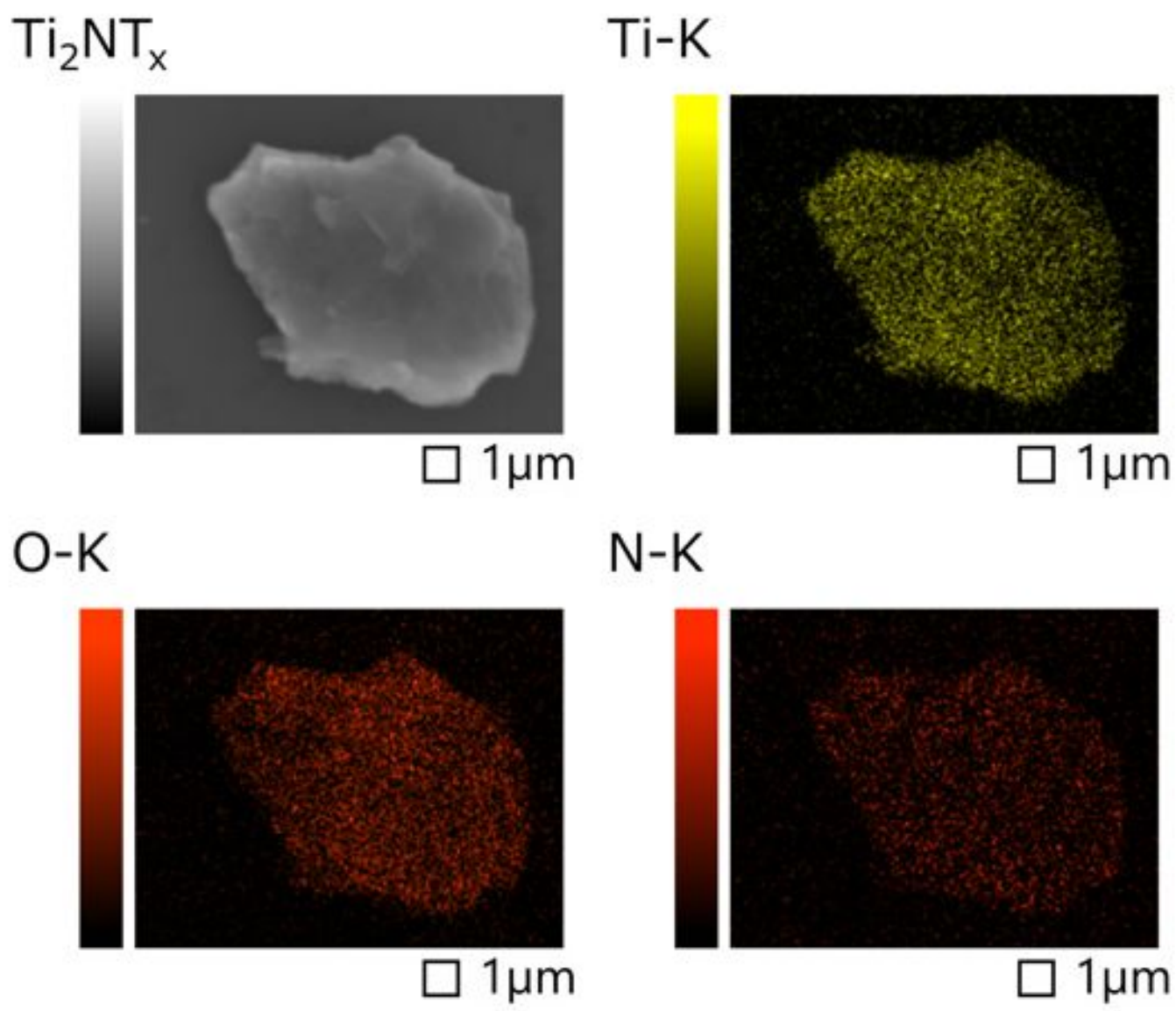

Figure S1. Energy-Dispersive X-ray Spectroscopy (EDS) mapping of Ti_2NT_x MXene.

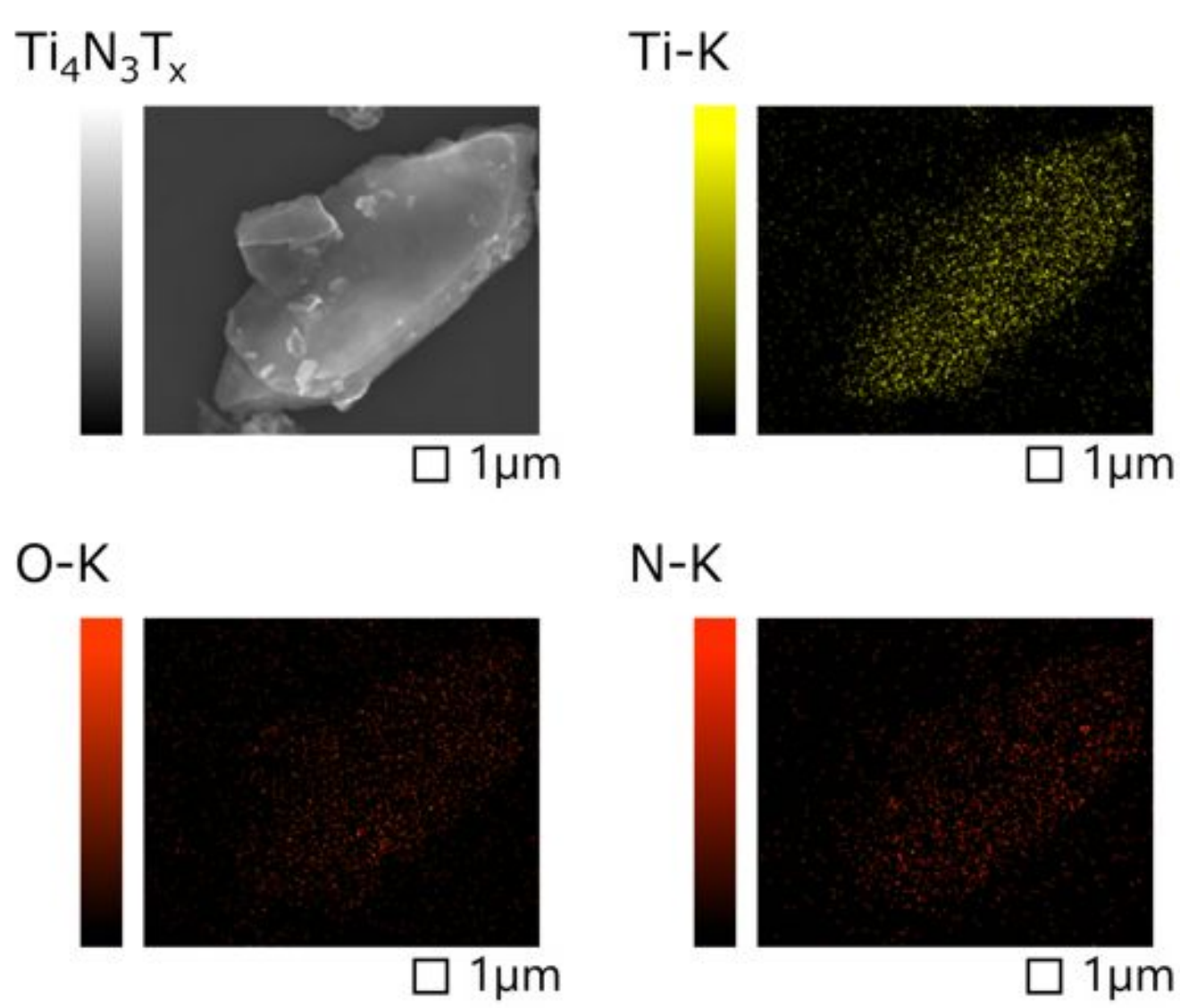

Figure S2. EDS mapping of $Ti_4N_3T_x$ MXene.

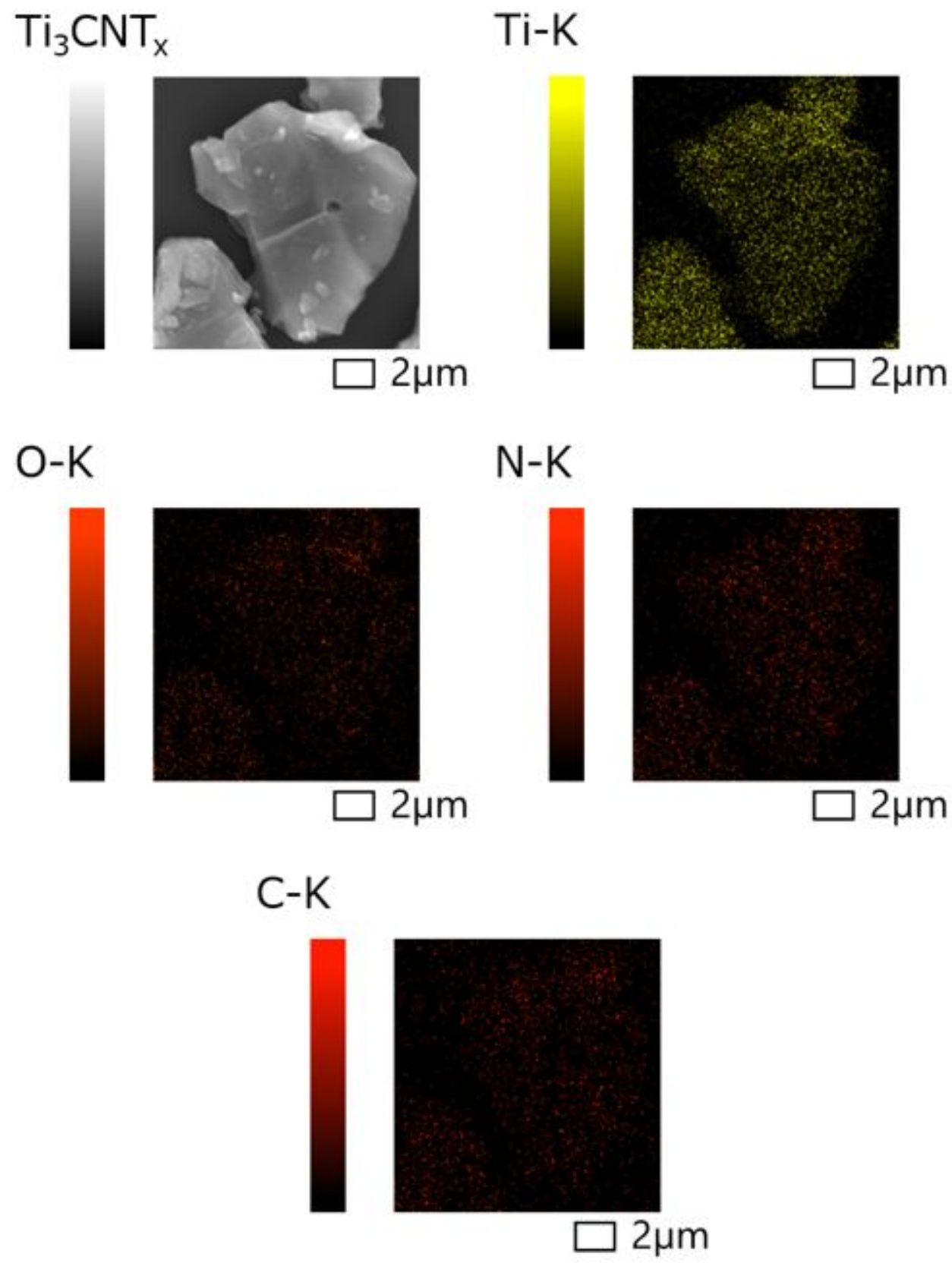

Figure S3. EDS mapping of Ti_3CNT_x MXene.

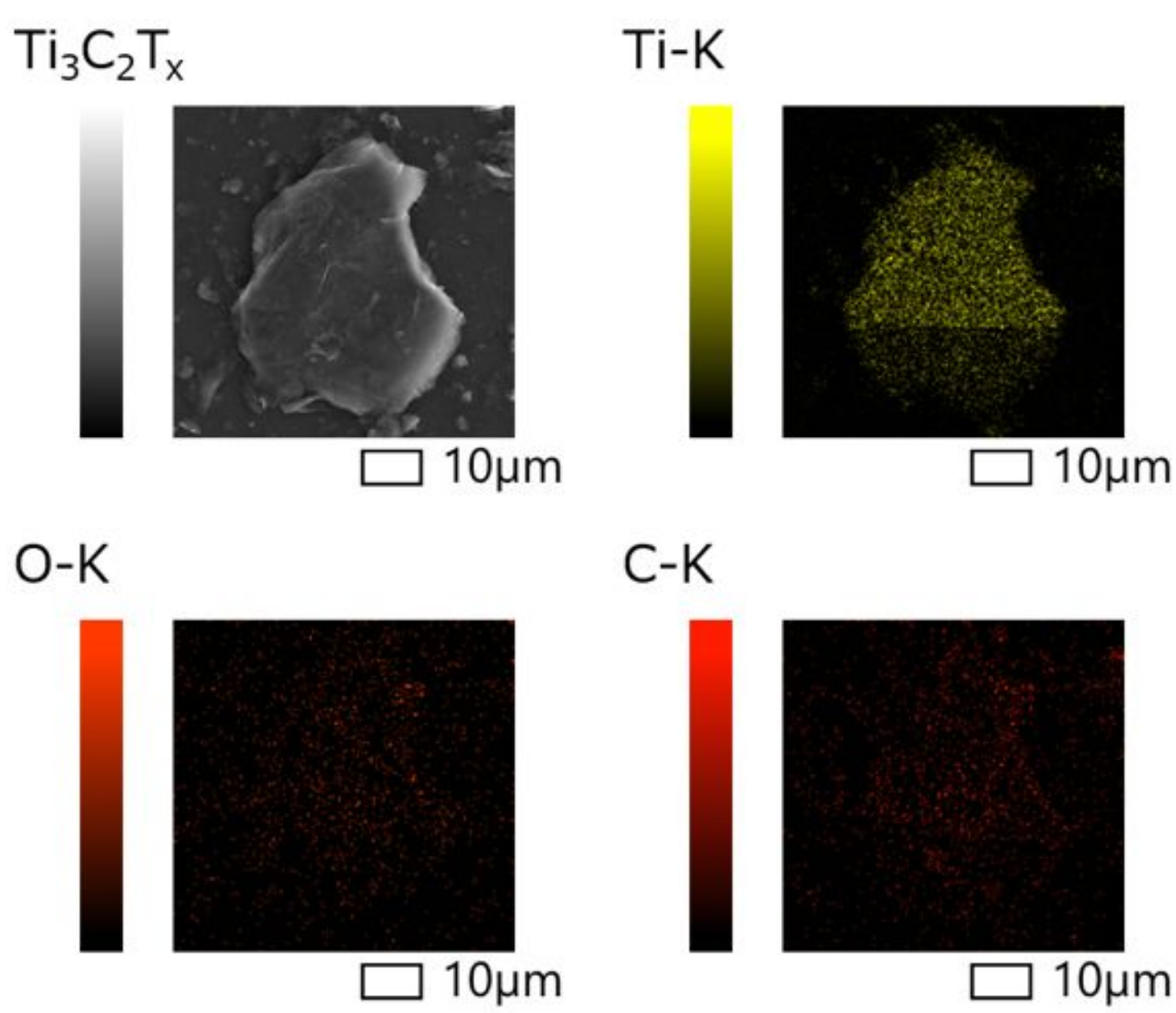

Figure S4. EDS mapping of $Ti_3C_2T_x$ MXene.

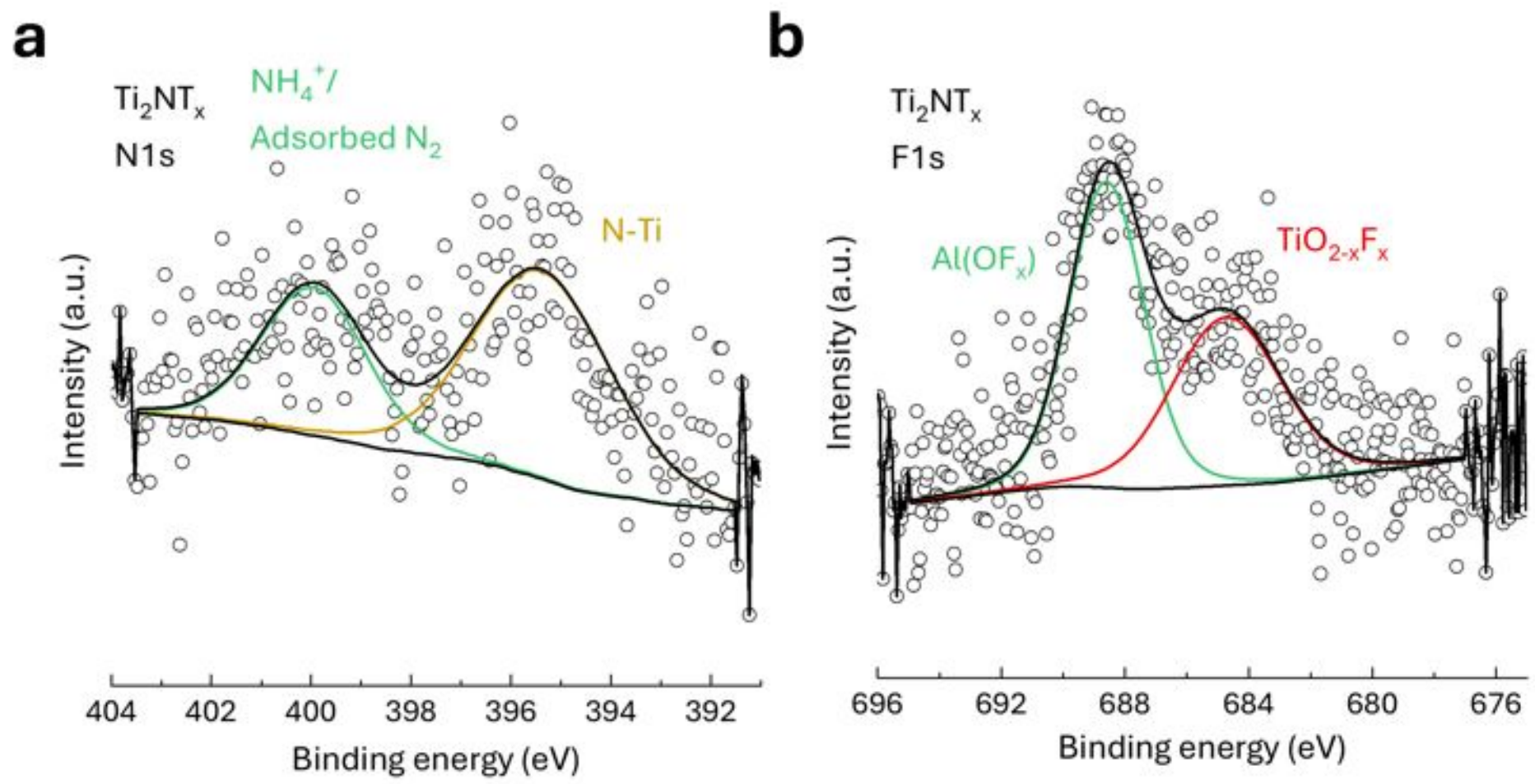

Figure S5. X-ray photoelectron spectroscopy (XPS) results of Ti_2NT_x MXene. (a) N1s, (b) F1s.

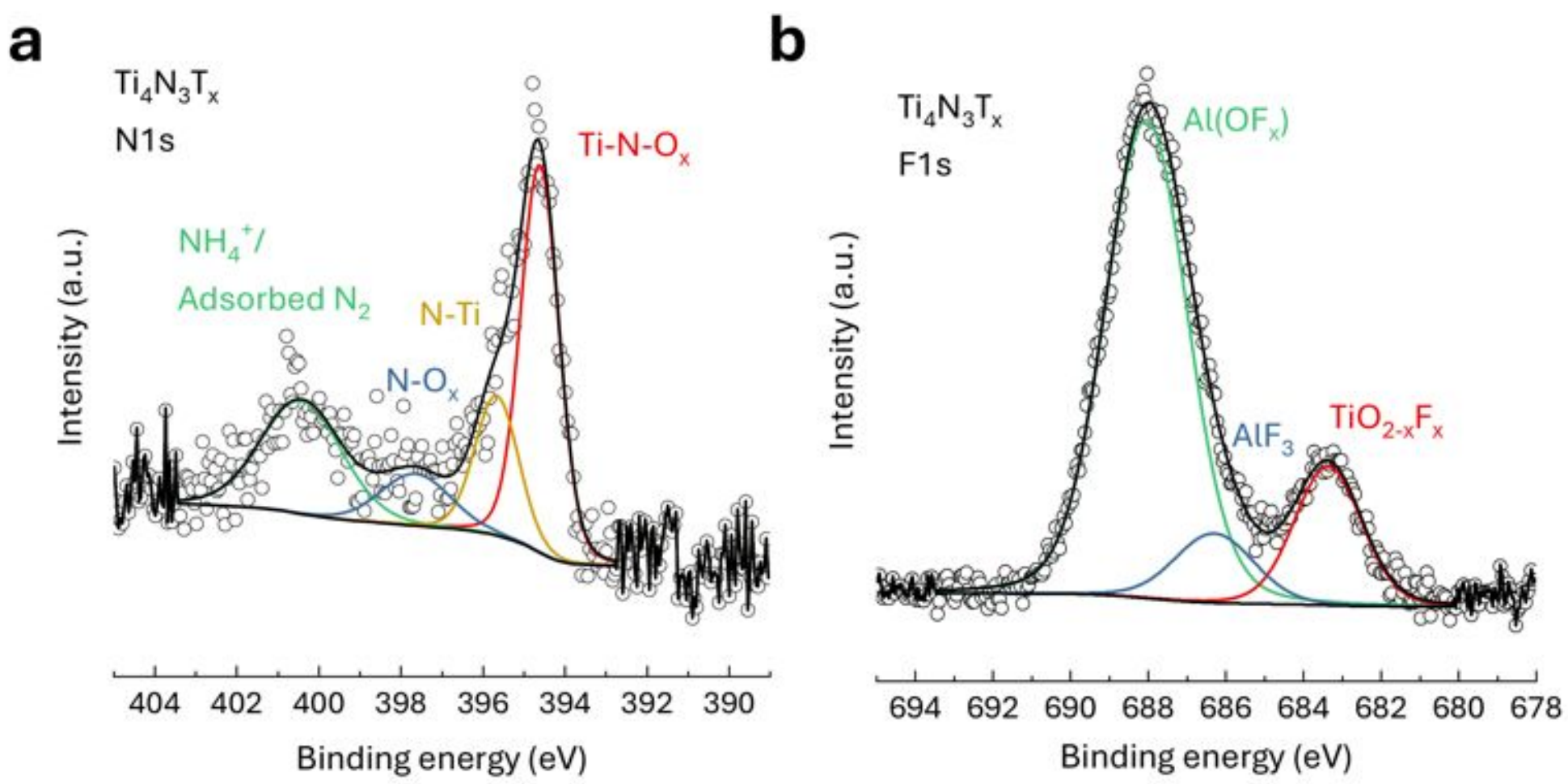

Figure S6. XPS results of $Ti_4N_3T_x$ MXene. (a) N1s, (b) F1s.

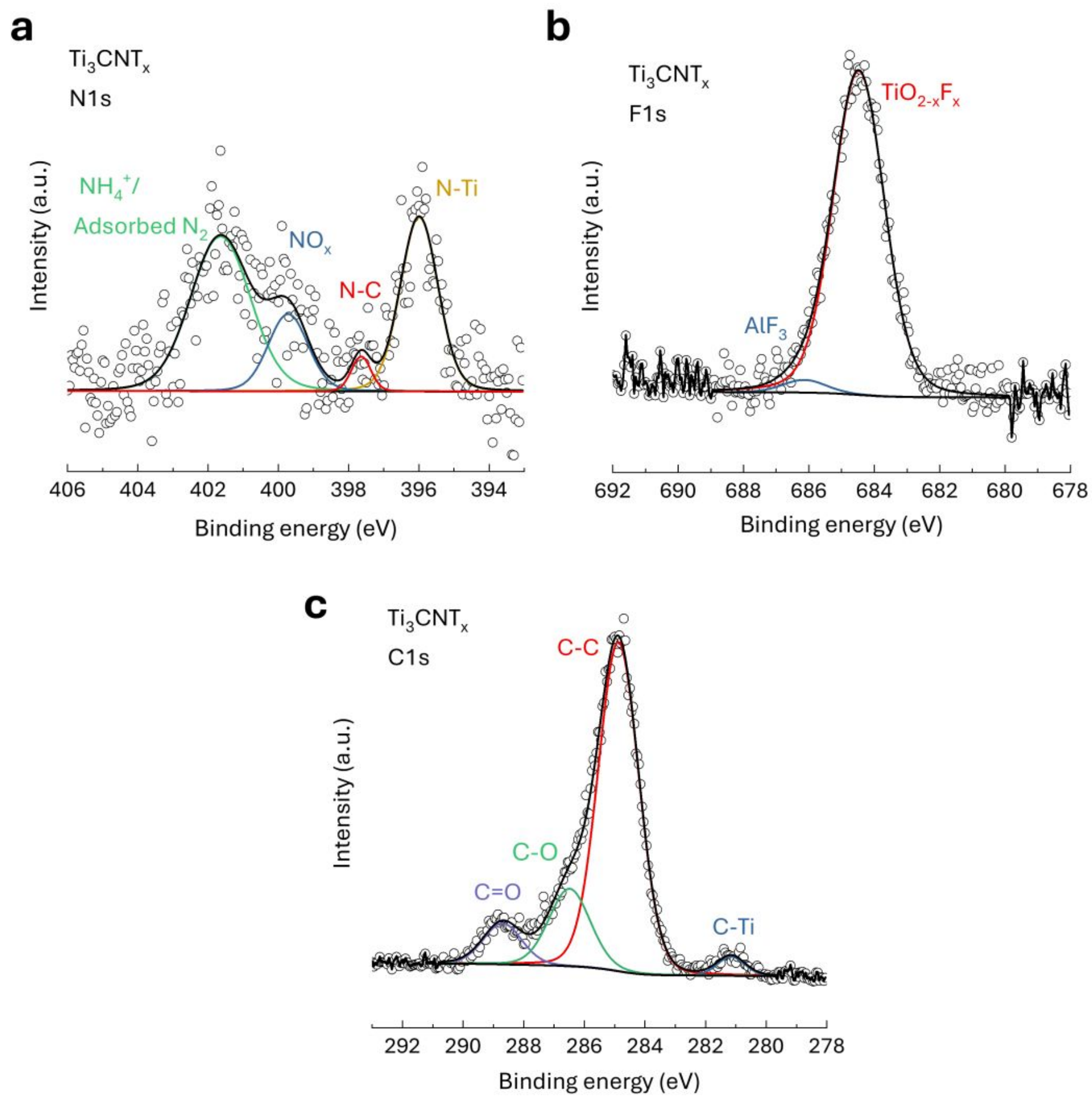

Figure S7. XPS results of Ti_3CNT_x MXene. (a) N1s, (b) F1s, (c) C1s.

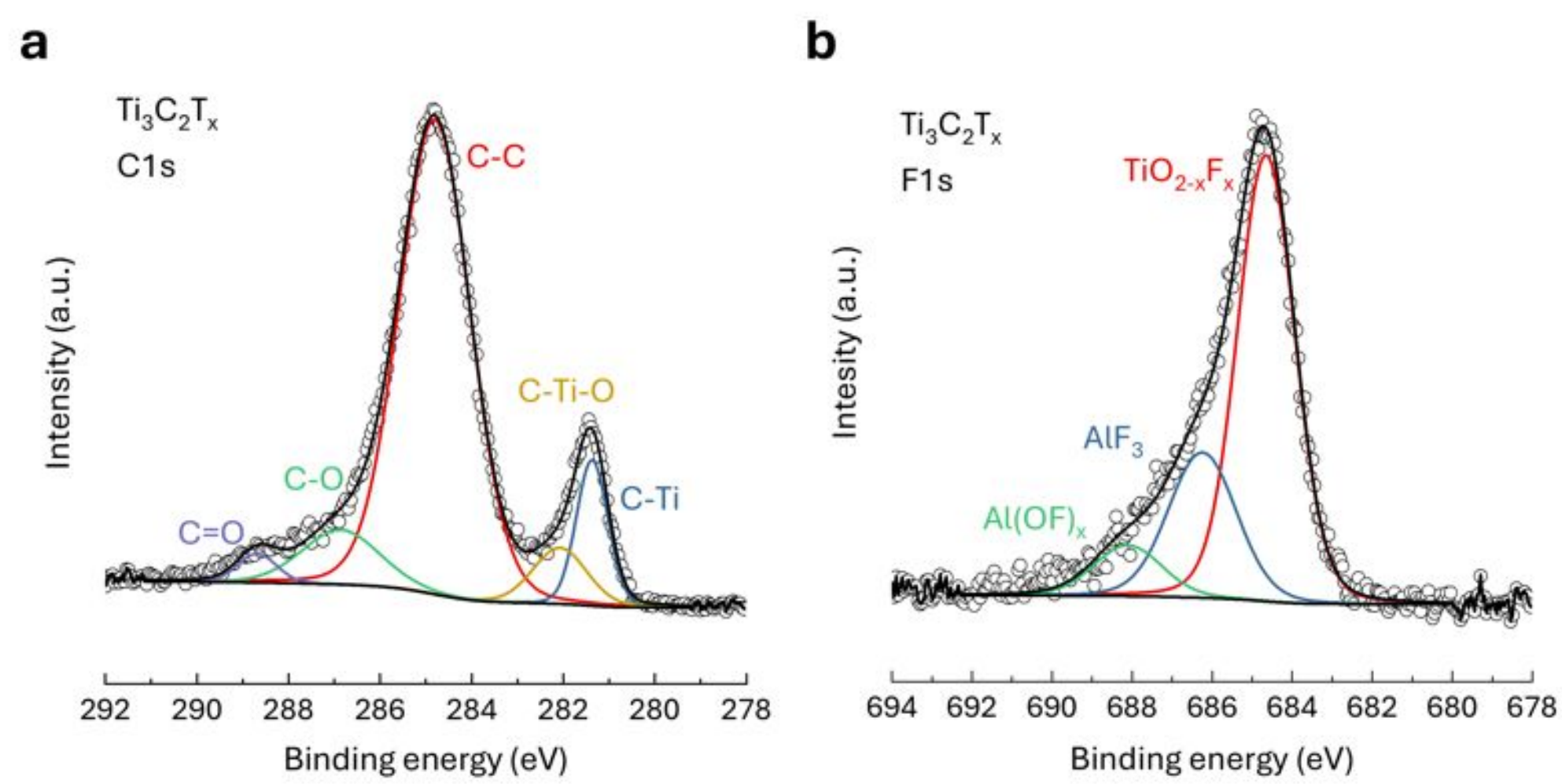

Figure S8. XPS results of $Ti_3C_2T_x$ MXene. (a) C1s, (b) F1s.

Table S1. XPS fitting results of delaminated MXenes.

		Binding Energy (eV)			
Region Spectra	Peak Assignment	Ti_2NT_x	$Ti_4N_3T_x$	Ti_3CNT_x	$Ti_3C_2T_x$
Ti $2p_{3/2}$ ($2P_{1/2}$)	Ti^{3+}	457.9 (463.7)	457.7 (463.6)	456.7(461.8)	456.9(462.4)
	Ti^{4+}	459.7(465.3)	458.8(464.2)	458.7(464.5)	459.0(464.8)
	Ti^{2+}	-	455.9(460.9)	455.2 (460.3)	455.49(460.9)
	Ti-C	-	-	-	454.64(459.9)
O 1s	TiO_2	529.2	529.2	-	-
	X-Ti-O_x	530.6	530.7	530.1	530.1
	X-Ti-$(OH)_x$	532.1	532.0	531.7	531.8
N 1s	Ti-N-O_x	-	394.6	-	-
	N-Ti	395.4	395.7	395.9	-
	N-O_x		397.6	399.7	-
	NH_4^+/Adsorbed N_2	399.9	400.4	401.6	-
	N-C	-	-	397.6	-
C 1s	C-Ti	-	-	281.1	281.4
	C-Ti-O	-	-	-	282.0
	C-C	-	-	284.8	284.8
	C-O	-	-	286.5	286.8
	C=O	-	-	288.7	288.7
F 1s	$Al(OF)_x$	688.6	688.0	-	688.1
	AlF_3	-	686.3	686.3	686.2
	$TiO_{2-x}F_x$	684.7	683.4	684.5	684.6

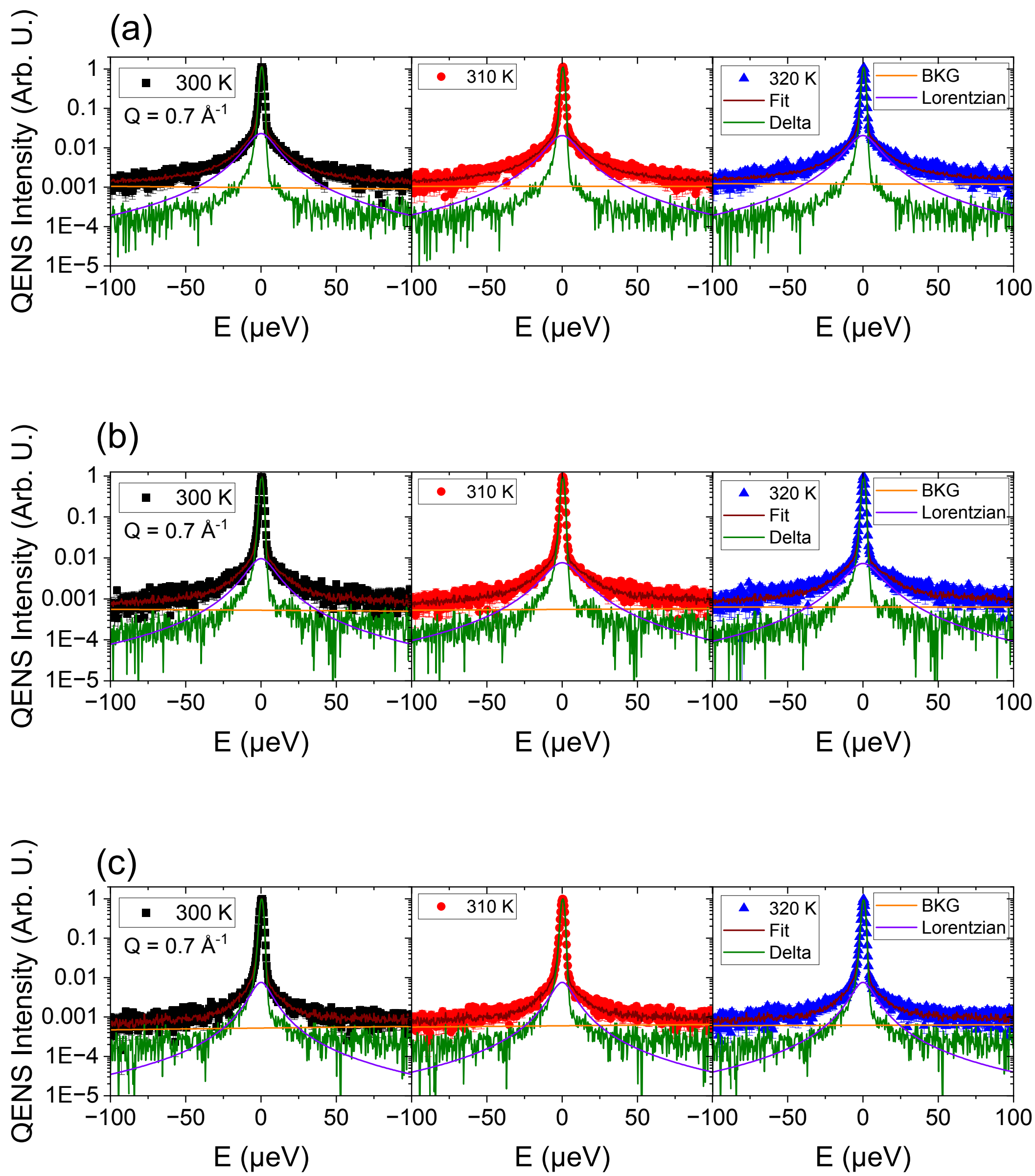

Figure S9. Representative QENS spectra at Q = 0.7 Å^{-1} along with model fits, as described in the main text, for (a) Ti_2NT_x, (b) $Ti_4N_3T_x$, and (c) Ti_3CNT_x MXenes. Symbols represent experimental data at the indicated temperatures.

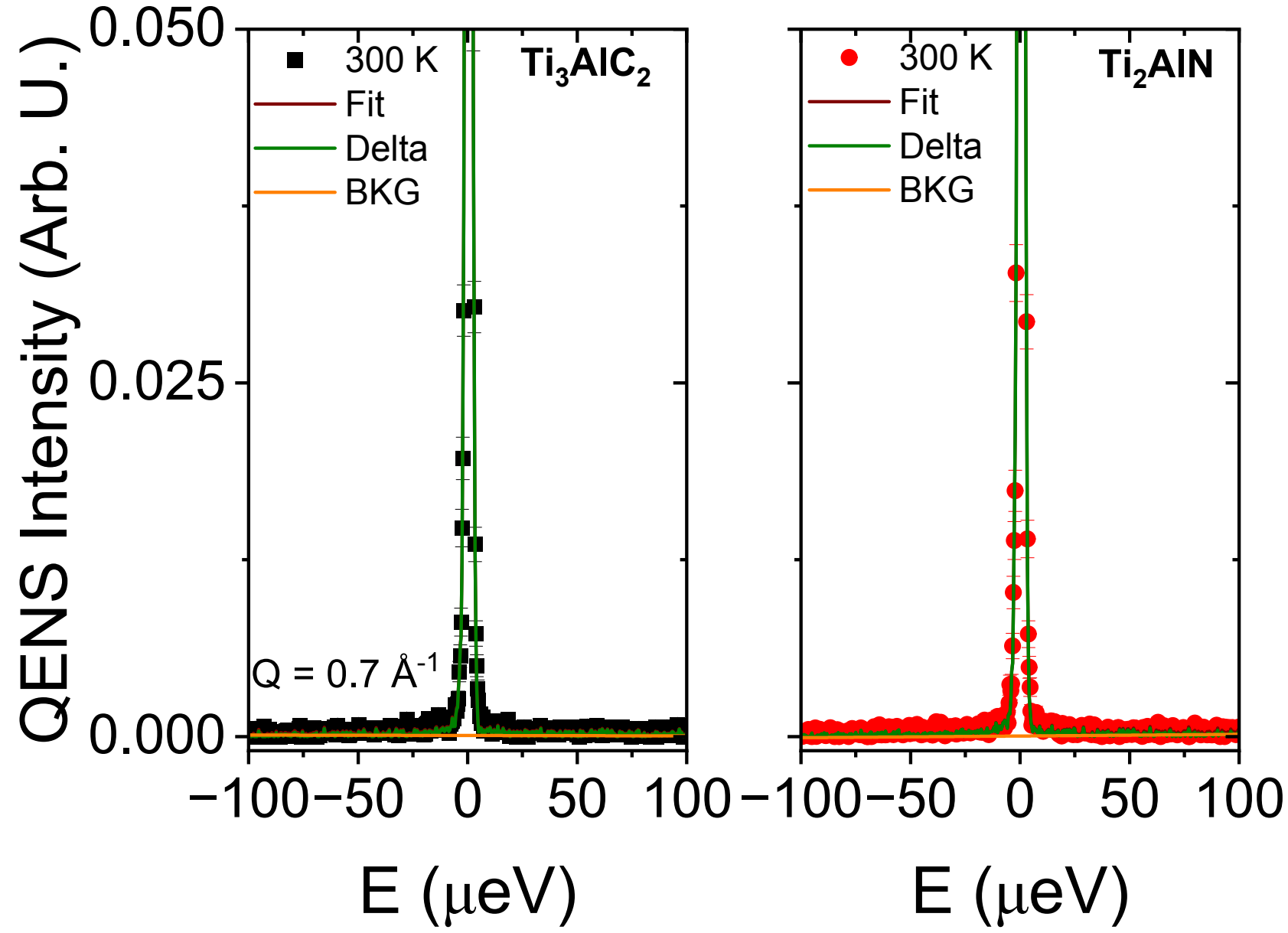

Figure S10. Representative QENS spectra of MAX phases at Q = 0.7 $Å^{-1}$ along with the model fit using a delta function

III. Discussion 1. Energetic Stability and Structural Configurations of MXene Systems

i. Optimized Atomic Configurations

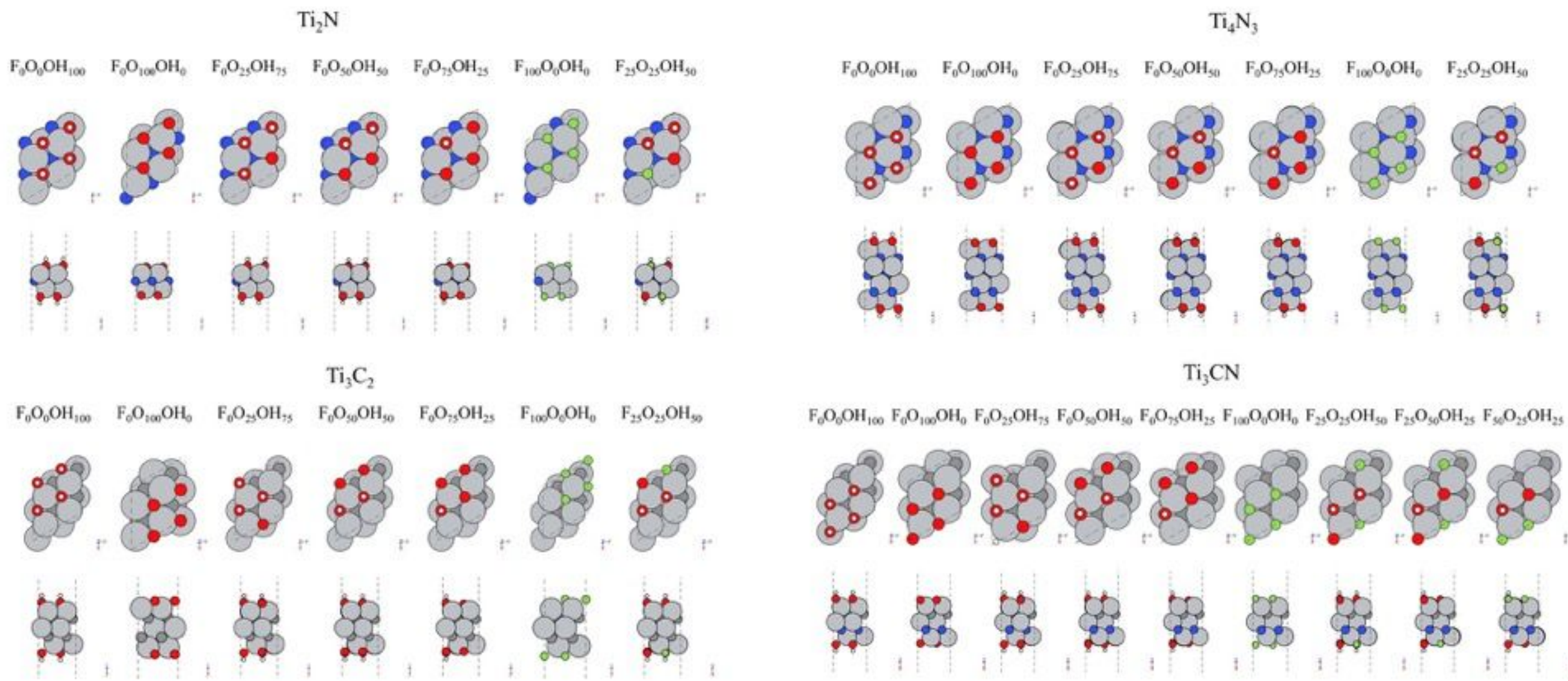

Figure S11. Illustrations of top and side views of the computationally optimized structures for four different functionalized MXene monolayers relaxed structures, including Ti_2NT_x, $Ti_4N_3T_x$, $Ti_3C_2T_x$, and Ti_3CNT_x with varying surface. The labels of the form ($F_xO_yOH_z$) denote the surface coverage percentage of each functional group above each column. Color Codes: Titanium (grey), Carbon (dark grey), Nitrogen (blue), Oxygen (red), Fluorine (green), and Hydrogen (white).

ii. Influence of Surface Terminations on Formation Energy

To quantitatively assess the thermodynamic viability of these structures, the formation energy (G_f) was calculated as a function of the surface termination composition, with the results compiled in **Figure S12**. A clear and consistent trend emerges from the analysis of binary compositions (**Figure S12a-c**). For all four MXene families, increasing the surface coverage of –F and –O groups results in a monotonic decrease in formation energy, signifying enhanced thermodynamic stability. Conversely, increasing the concentration of –OH groups is energetically less favorable than forming -F or -OH in vacuum. This fundamental energetic hierarchy is rooted in the high electronegativity of fluorine and oxygen, which allows them to form exceptionally strong, stable bonds with the surface titanium atoms. The hydroxyl group, being less electronegative and structurally more complex, forms a comparatively weaker bond. A more comprehensive view of the stability landscape is provided by the ternary diagrams in **Figure S12d-g**, which map the

formation energy across a wide array of mixed-termination compositions. These diagrams consistently show that the regions of greatest stability, indicated by the most negative formation energies (blue color map), are located in the corners of the diagrams corresponding to high concentrations of -F and -O. This analysis confirms that, at 0 K, surfaces dominated by fluoride and oxide terminations are thermodynamically preferred over those rich in hydroxyl groups.

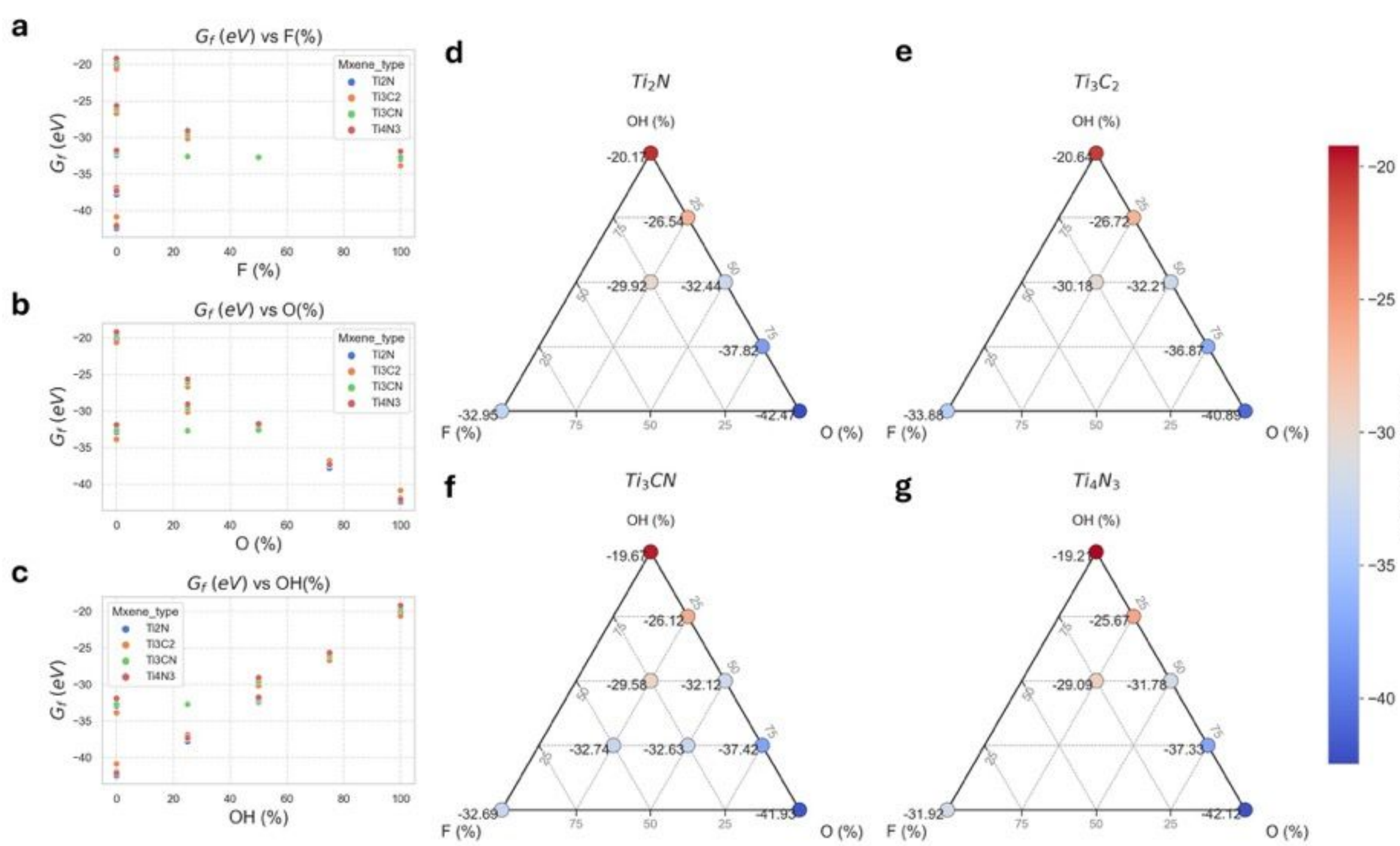

Figure S12. Thermodynamic stability and Formation energy plots of four MXenes (Ti_2NT_x, $Ti_4N_3T_x$, $Ti_3C_2T_x$, and Ti_3CNT_x) with mixed surface. (a-c) Formation energy (G_f) relative to its Mxene naked surface of four MXene types as a function of the percentage of individual surface terminations at 0 K: (a) Fluorine, (b) Oxygen, and (c) Hydroxyl. (d-g) The ternary diagrams plot the calculated formation energy (eV) against the relative surface coverage of monolayers with various compositions of F, O, and OH functional groups. Each point on the diagram corresponds to a specific surface composition, with the relative formation energy to its MXene naked surface. The trends in both figures indicate that for all systems, configurations rich in -F and -O are energetically more favorable than those with a high concentration of OH groups for monolayer MXene surface at 0K, as shown by the trend in binary plots and increasingly negative formation energies in ternary plots (blue region of the colormap).

IV. Discussion 2. Interfacial Water Structure and Hydrogen Bonding Network

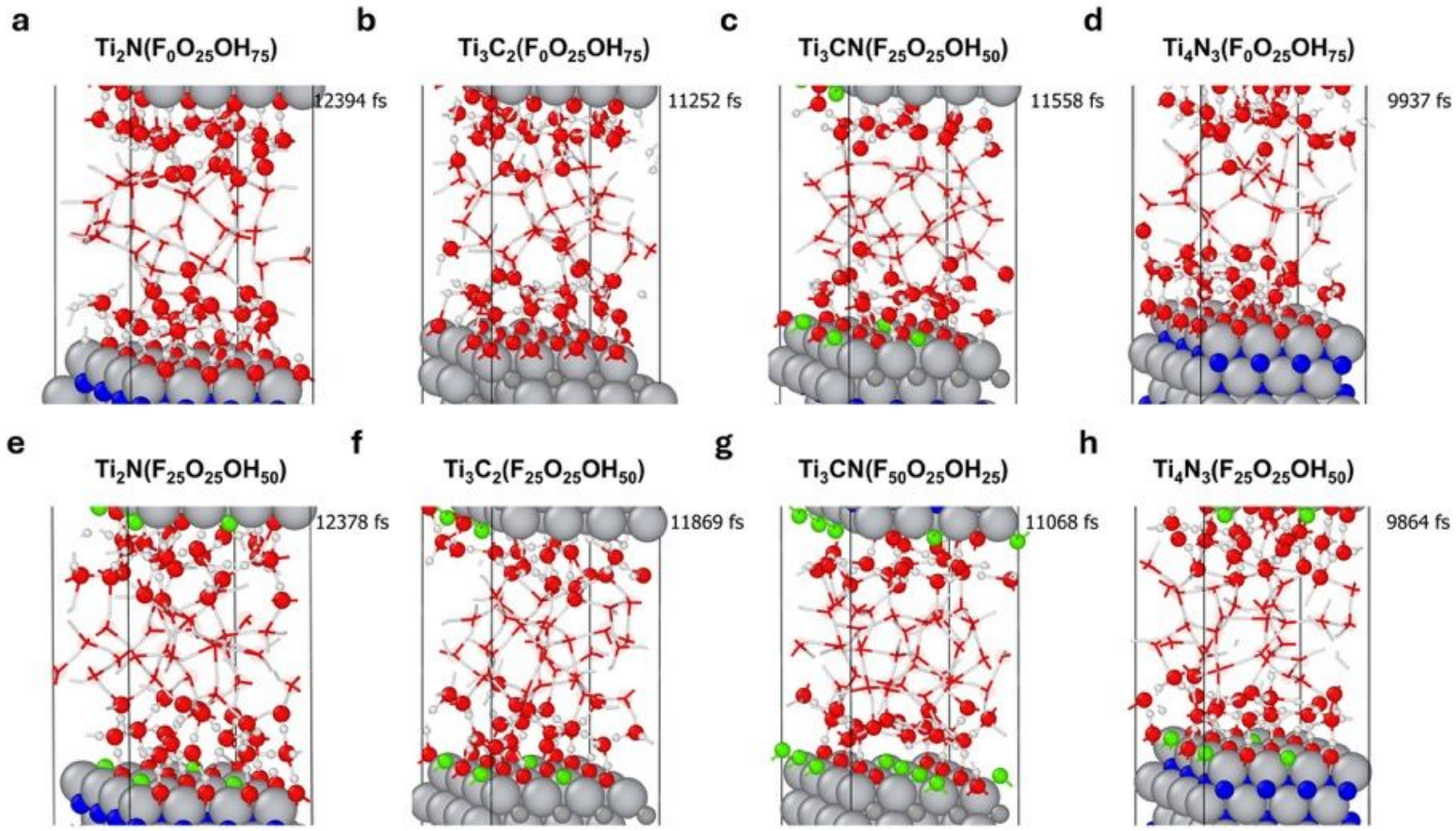

Figure S13. Final configuration snapshots from AIMD simulations illustrating the structure of liquid water on different functionalized MXene surfaces at 298 K. The figure displays two different termination compositions for each of the three of MXenes Ti_2NT_x, $Ti_3C_2T_x$, and $Ti_4N_3T_x$, and higher -F concentration with lower -OH concentration on Ti_3CNT_x surface to approach experiment surface compositions. The specific surface chemistry and the simulation time (in fs) are indicated for each panel. Overall, all functionalized MXene surfaces are highly hydrophilic, forming a dense icy hydrogen-bonding network with especially -OH functional groups. Higher -OH concentration likely leads to a denser but less organized hydrogen-bonding network at the interface. However, Counter-intuitively, fluorine (-F) termination causes water molecules in the first layer to fixate on nearby hydroxyl (-OH) groups and form organized structures. Conversely, on a surface with more -OH groups, these water molecules are more dynamic, readily moving between the different hydroxyl sites.

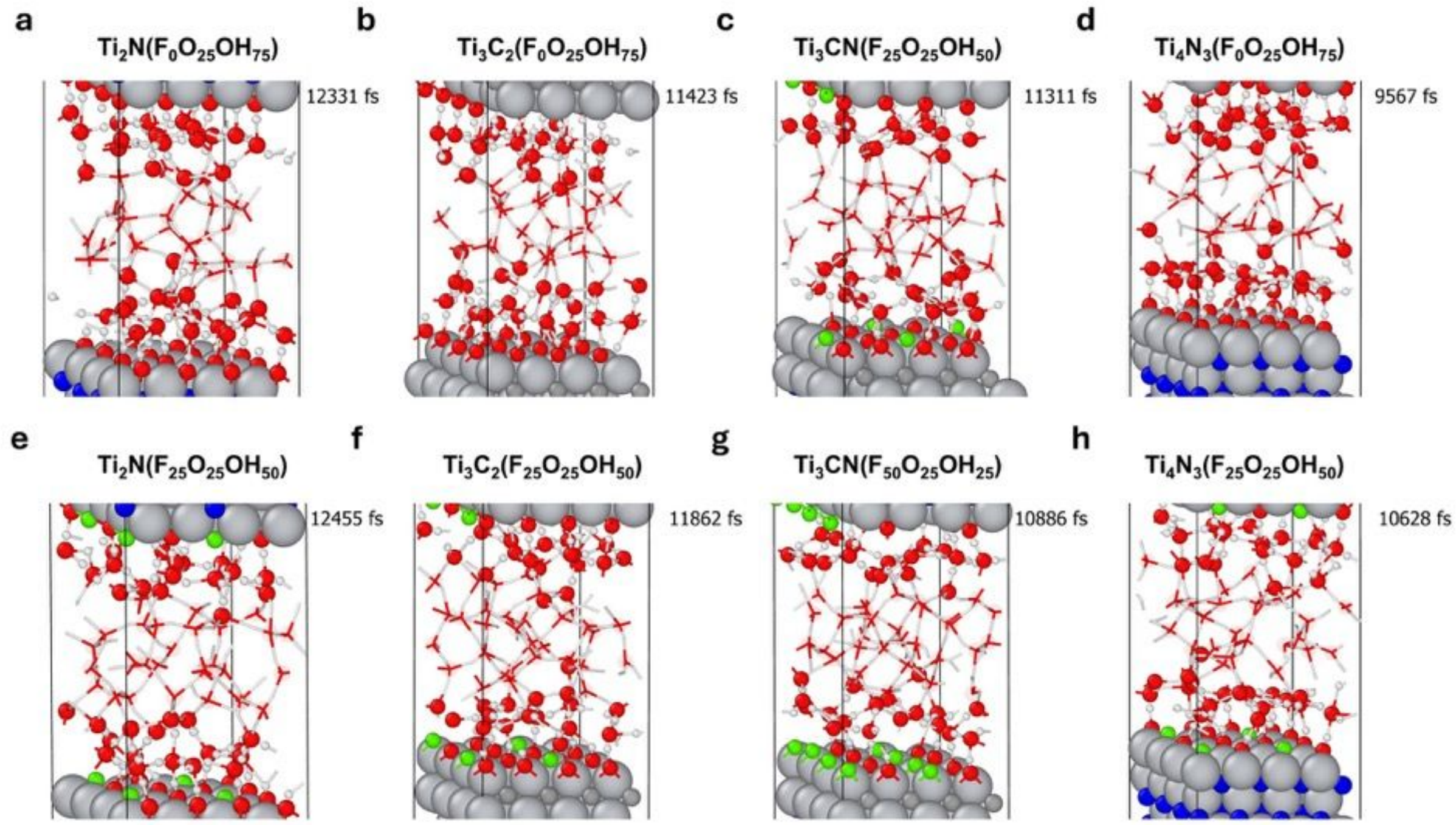

Figure S14. Final configuration snapshots from AIMD simulations illustrating the structure of liquid water on different functionalized MXene surfaces at 328 K. The figure displays two different termination compositions for each of the three of MXenes Ti_2NT_x, $Ti_3C_2T_x$, and $Ti_4N_3T_x$, and higher -F concentration with lower -OH concentration on Ti_3CNT_x surface to approach experiment surface compositions. The specific surface chemistry and the simulation time (in fs) are indicated for each panel. Overall, all functionalized MXene surfaces are still highly hydrophilic compared to 298K, forming an icy hydrogen-bonding network with especially -OH functional groups. However, the hydrogen bonding between water and -OH groups become more dynamic, fluid, and energetic environment and less crystalline form.

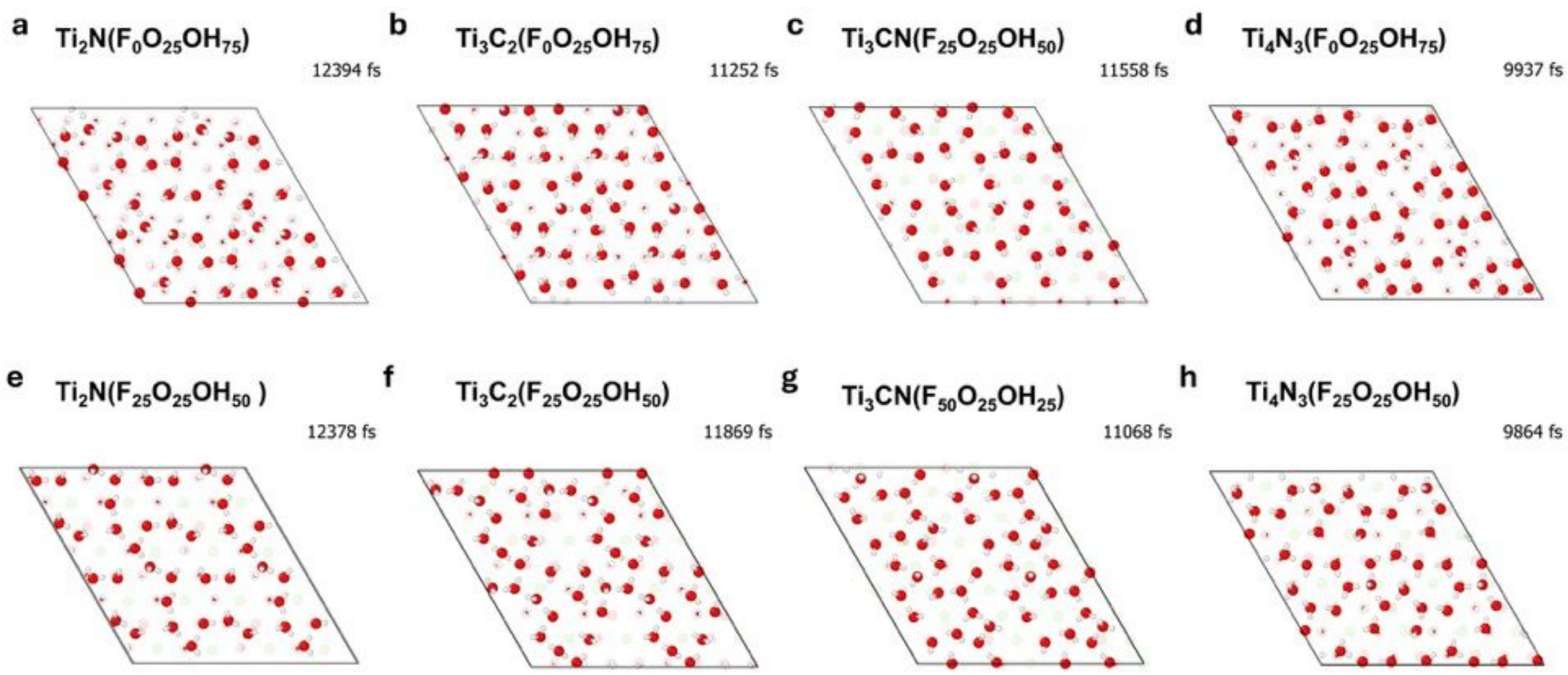

Figure S15. The simulations reveal that water molecules consistently avoid residing above fluorine (-F) terminations, preferring instead to form hydrogen bonds with hydroxyl (-OH) and oxygen (-O) groups. This interaction preference governs the structural organization of the water molecules. For example, as the -F concentration on $Ti_3C_2T_x$ increases from 0% to 25%, the dominant water structures shift from 4- and 5-membered rings to 5- and 6-membered rings. In contrast, on the Ti_3CNT_x surface, increasing the -F concentration from 25% to 50% causes the water network to revert to a more grid-like pattern composed primarily of 4- and 5-membered rings, demonstrating that both the surface type and its chemical termination are critical to interfacial water structure.

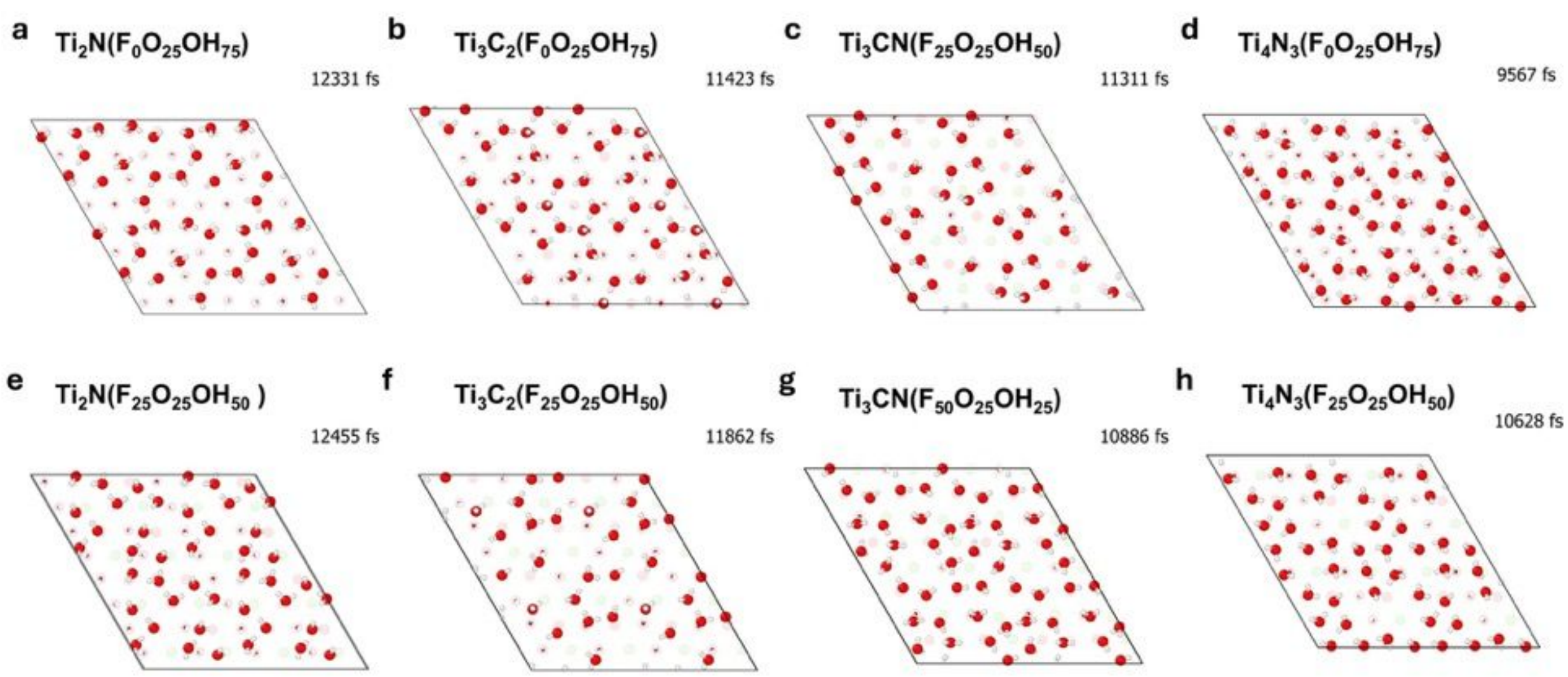

Figure S16. Top-down snapshots from AIMD simulations of the final configuration, showing the structure of the first water layer on various MXene surfaces at 328 K. Other notations are the same as in **Figure S15**. With the temperature increase from 298 K to 328 K, we observe that more of

the water molecules' OH groups point upwards. While the system still shows a preference for forming hydrogen bonds (HBs) with the MXene surface and between water molecules in the same layer, this observation suggests a slight relative weakening of the MXene-OH hydrogen bonds as all HBs become weaker at a higher temperature.

V. Discussion 3. Quantitative Analysis of the Hydrogen Bond Network

Overall, In **Figure 5a-b**, the total number of hydrogen bonds is primarily driven by the concentration of surface hydroxyl (–OH) groups. The Ti_2NT_x (dark blue line) has the highest normalized number of hydrogen bonds (N_{HB}) at both 298 K and 328 K. Although both the Ti_2NT_x and $Ti_3C_2T_x$ systems have 75% –OH termination, the Ti_2NT_x surface (dark blue, light blue) sustains more hydrogen bonds than the $Ti_3C_2T_x$ surface (dark green, light green). As the temperature increases from 298 K to 328 K, the N_{HB} for the Ti_3CNT_x surface remains almost unchanged, while the N_{HB} for Ti_2NT_x and $Ti_4N_3T_x$ drop.

To deconstruct the network's architecture, the total N_{HB} was separated into contributions from interactions between the MXene slab and water (slab-water) and interactions between water molecules themselves (water-water). The time evolution of these components is shown for strong HBs in **Figure S18** and for medium-strong HBs in **Figure 5c-f**. In a water slab medium between 298K and 328K, we observed a significant change (~0.1) in medium-strong hydrogen bonds (N_{HB}) for $Ti_2NT_x(F_0O_{25}OH_{75})$ and $Ti_3CNT_x(F_0O_{25}OH_{75})$, whereas other systems showed only minor changes. Within the same temperature range, the strong hydrogen bonds of a $Ti_2NT_x(F_0O_{25}OH_{75})$, $Ti_3CNT_x(F_0O_{25}OH_{75})$ and $Ti_4N_3T_x(F_0O_{25}OH_{75})$ also exhibited a more pronounced change compared to the others shown in **Figure S18**. These results suggest that a higher -OH concentration is significantly affected by temperature. Furthermore, it indicates that hydrogen bonding interaction of Nitride and Carbonitride MXenes between MXene and water are more susceptible to thermal effects than $Ti_3C_2T_x$ MXene.

It reveals a critical trade-off at the interface, governed by surface chemistry. An example is the Ti_3CNT_x surface, which, due to its high –F content, forms the lowest number of slab-water hydrogen bonds. However, it simultaneously promotes a relatively high number of water-water bonds, even more than observed for some high-OH Ti_2NT_x surfaces. This behavior suggests that the relatively hydrophobic nature of the Ti_3CNT_x surface reinforces the cohesion of the water

network. Decreasing in forming stable strong bonds with the MXene, the interfacial water molecules turn inward, strengthening their interactions with other waters. This effectively shifts the nature of the interface from strongly adhesive (dominated by slab-water interactions) to relative cohesive (dominated by water-water interactions), shows a profound implication for transport within MXene at the nanoscale.

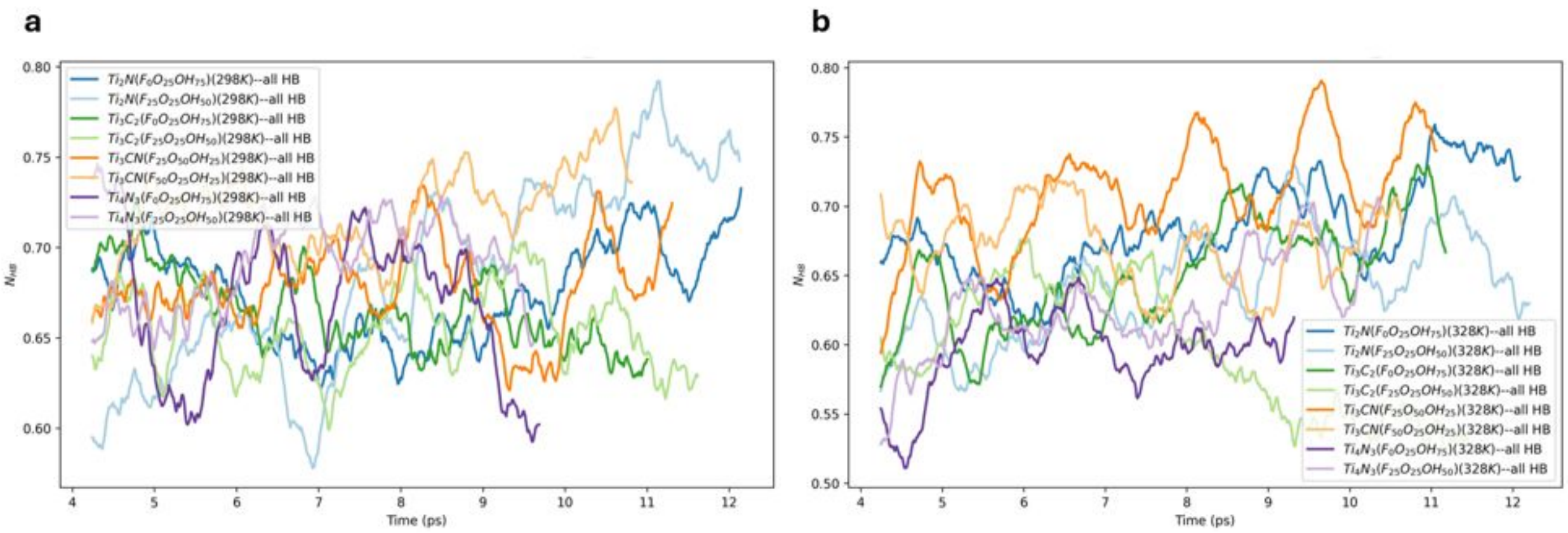

Figure S17. Illustration of comparison of hydrogen bonding dynamics across different functionalized MXene systems simulated at (a) 298 K and (b) 328 K from a 9–11 ps AIMD simulation, considering strong hydrogen bonds (2.7 Å cutoff). The normalized number of hydrogen bonds (N_{HB}) is plotted as moving average of 50 points (0.05ps average), showing a semi-equilibrium region from approximately 4 ps to 11 ps. Each line corresponds to a unique combination of MXene type and surface termination (–F, –O, –OH). Overall, Ti_2NT_x and Ti_3CNT_x show a slightly higher normalized number of both medium-strong and strong hydrogen bonds, which is mainly due to the intrinsic properties of the MXene. The number of strong hydrogen bonds constitutes 40-50% of the number of medium-strong hydrogen bonds. The overall N_{HB} range changes from 0.60-0.75 at 298 K to 0.55-0.70 at 328 K.

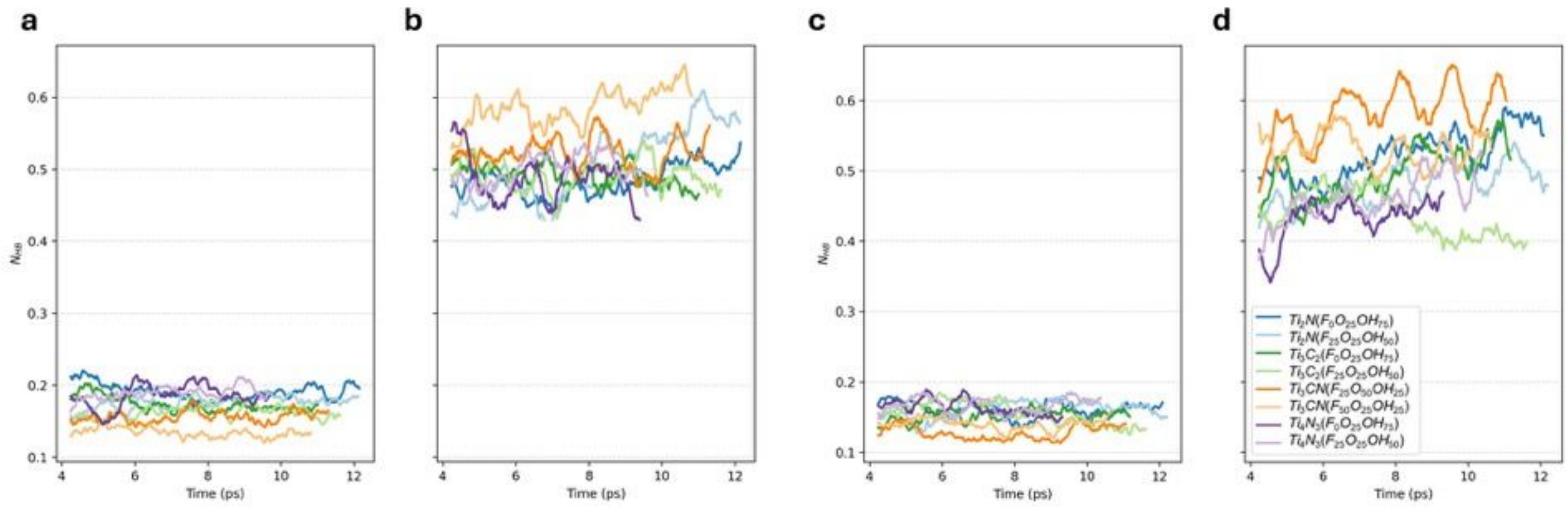

Figure S18. Illustration of time evolution of the moving average (0.05ps) for the normalized number of strong hydrogen bonds (N_{HB}), defined by a 2.7Å cutoff, for various MXene compositions at (a-b) 298 K and (c-d) 328 K. Panels (a) and (c) depict hydrogen bonds between the MXene slab and water molecules (slab-water), while (b) and (d) show those between water molecules (water-water). Overall, the slab-water N_{HB} reaches equilibrium faster, stabilizing with fluctuations of approximately 0.025. In contrast, the water-water N_{HB} relaxes more slowly and exhibits larger fluctuations of around 0.1. Interestingly, $Ti_4N_3T_x$ and Ti_2NT_x form a higher number of slab-water hydrogen bonds (N_{HB}) at both 298 K and 328 K. Conversely, Ti_3CNT_x shows the lowest number of slab-water bonds. However, it promotes a relatively high number of water-water bonds (N_{HB}), even more than is observed for Ti_2NT_x. It suggests that Ti_3CNT_x surface with more -F presents a relative hydrophobic surface to Ti_2NT_x reinforce the water-water interaction and weaken the water-slab interaction.

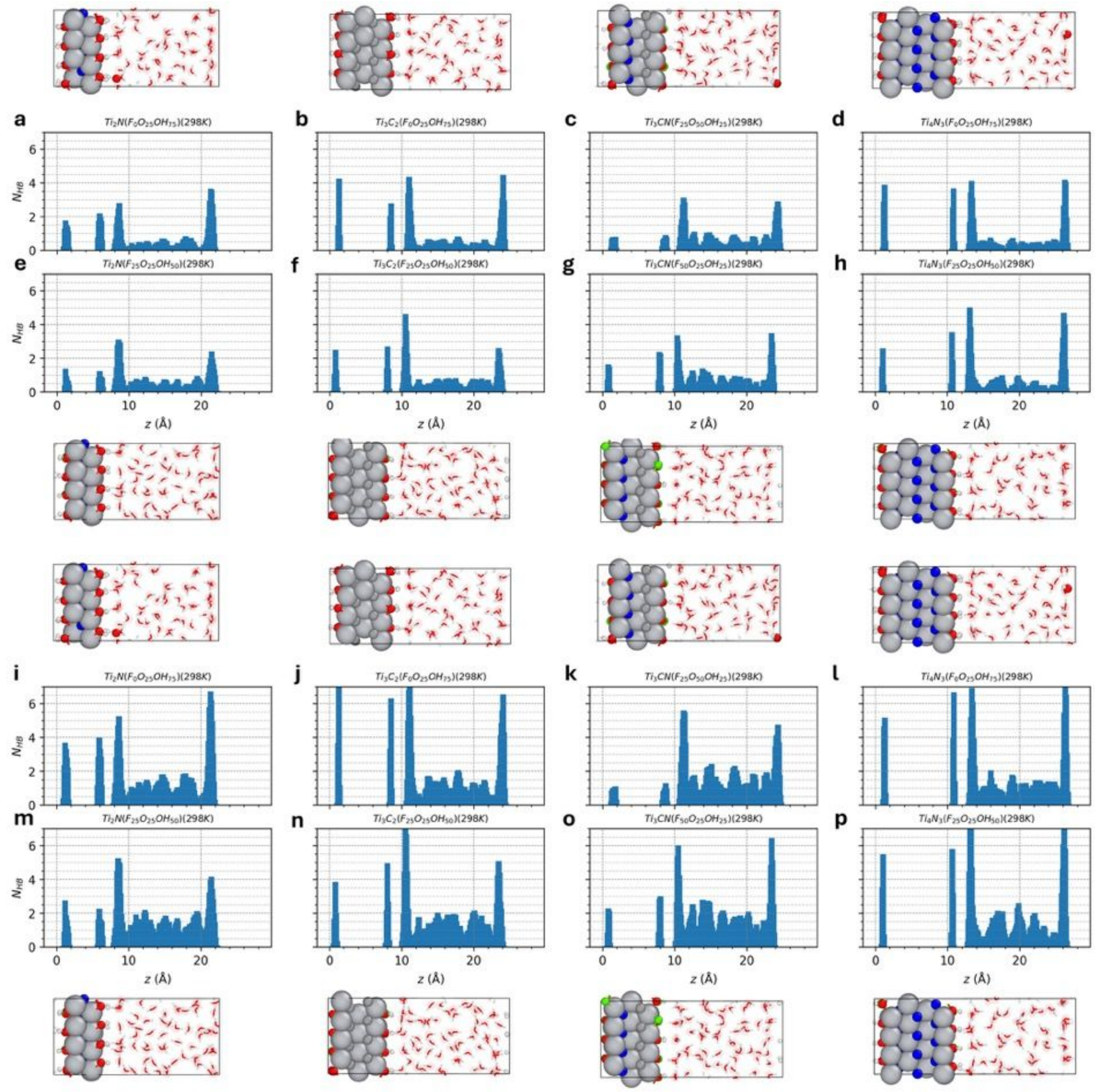

Figure S19. Average accumulation normalized hydrogen bonding results (4ps to ~10ps) for various MXene-water interfaces at 298 K and analysis with strong HB cutoff (2.7 Å, upper subplots) or medium-strong to strong HB cutoff (2.9 Å, lower subplots). The top and bottom rows show representative snapshots for different functionalized MXene surfaces, as labeled in the corresponding plots near them. The central graphs illustrate the number of hydrogen bonds (N_{HB}) as a function of position of the donor oxygen atom (z) perpendicular to the MXene slab. Most strong hydrogen bonds near the surface are formed in one of two ways: either from the hydrogen of a surface hydroxyl (-OH) group acting as a donor, or to an oxygen atom in the first water layer acting as an acceptor. In contrast, few strong hydrogen bonds are found between the water layers themselves.

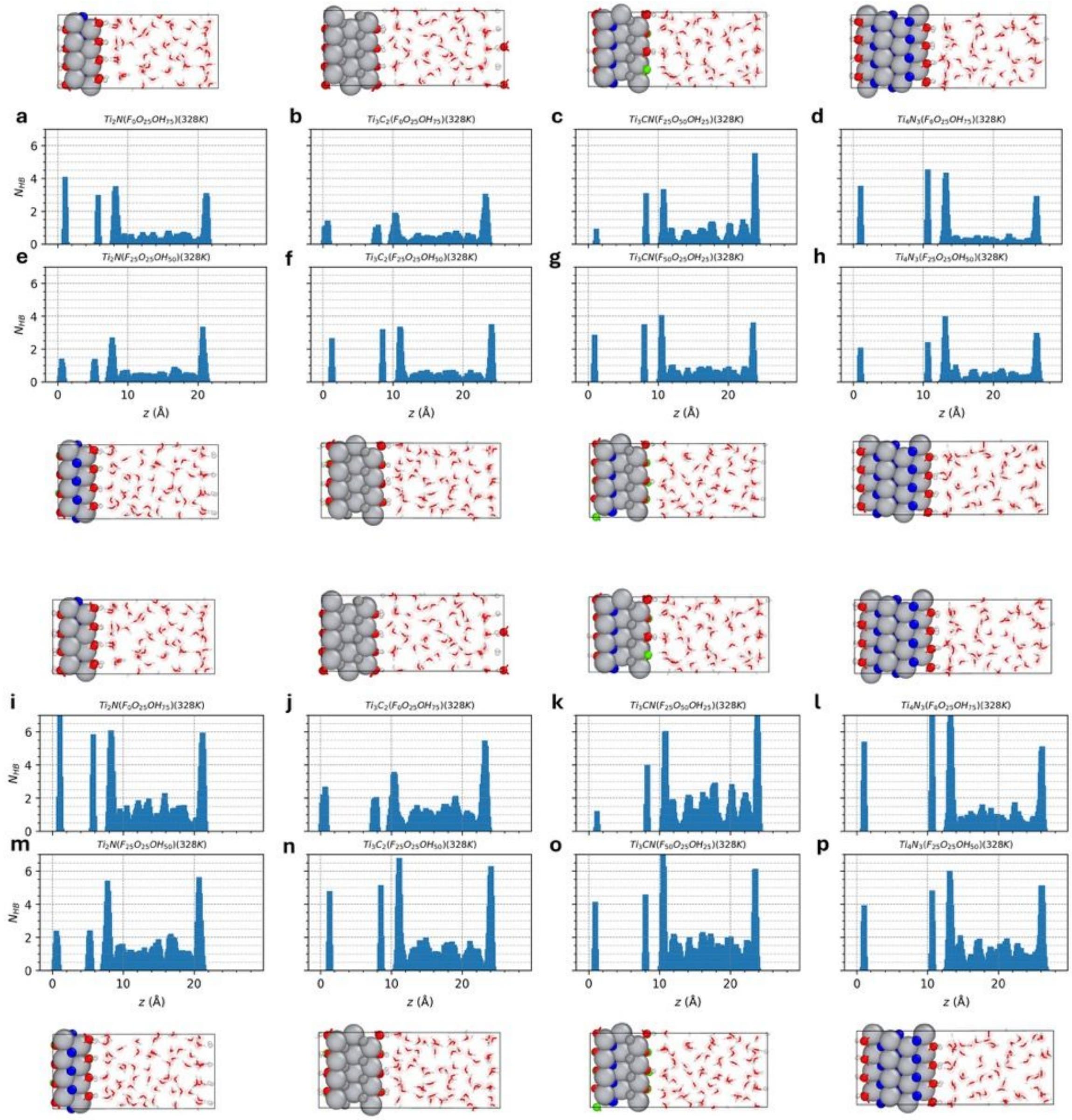

Figure S20. Average accumulation normalized hydrogen bonding results (4ps to ~10ps) for various MXene-water interfaces at 328 K and analysis with strong HB cutoff (2.7 Å, upper subplots) or medium-strong to strong HB cutoff (2.9 Å, lower subplots). The top and bottom rows show representative snapshots for different functionalized MXene surfaces, as labeled in the corresponding plots near them. The central graphs illustrate the number of hydrogen bonds (N_{HB}) as a function of position of the donor oxygen atom (z) perpendicular to the MXene slab. As temperatures increase, the peaks become boarding.

Interesting, few very strong hydrogen bond could be discovered within the threshold of 2.7 Å between water and MXene, suggesting very strong hydrogen bonding formation between water and MXene slab.

Overall, for strong hydrogen bonds (HBs) (<2.7 Å), the number of hydrogen bonds (N_{HB}) on the MXene surface and in the first water layer is related to the surface terminations and the MXene type. Specifically, the N_{HB} from HB donors on the MXene surface decreases with the introduction of more -F groups in Ti_2NT_x, $Ti_3C_2T_x$, and $Ti_4N_3T_x$. However, the N_{HB} on MXene surface increases when -F surface terminations are raised from 25% to 50% by replacing -O functional groups. For the first water layer, the N_{HB} from HB donors decreases for Ti_2NT_x and $Ti_3C_2T_x$ but slightly increases for Ti_3CNT_x and $Ti_4N_3T_x$ when introducing more -F.

When including medium-strong to strong HBs (<2.9 Å), we observe a much higher N_{HB} at the surface and in the first water layer in both $Ti_4N_3T_x$ and $Ti_3C_2T_x$ compared to the other systems. Conversely, $Ti_3C_2T_x$ and Ti_2NT_x show relatively weaker HB interactions with a lower N_{HB}. Again, an opposite trend is observed as the number of -F groups on Ti_3CNT_x increases; a higher N_{HB} is observed on the MXene surface, while all other cases show a significant decrease in N_{HB} at the MXene surface.

When considering the water layer structures, we found that Ti_3CNT_x with 25% or 50% -F terminations show more organized water layers, and $Ti_4N_3T_x$ with 25% -F shows a similar trend. However, this behavior is less pronounced in Ti_2NT_x and $Ti_3C_2T_x$. As temperature increase, the peaks show similar trend for N_{HB} of MXene surface and 1st water layer, but the water-water structure become slightly unclear, and suggest a less organized water bulk layers as temperatures increase. Interestingly, the Ti_3CNT_x with 25% -F water layers structures maintain when temperatures increase.

VI. Discussion 4. MXene Interlayer Water Structure

To probe the finer details of water arrangement within the structured MXene interlayers, radial distribution functions (RDFs) and angular analyses were employed. Pairwise RDFs provide quantitative measures of the average intermolecular distances. The O-H RDFs, shown in **Figure S21**, display a sharp first peak at approximately 1.8 Å for both slab-water and water-water pairs. This is the classic signature of a hydrogen bond, corresponding to the distance between a hydrogen-bond donor (H) and an acceptor (O). As for O-O RDFs, presented in **Figure S22**, offer deeper insights into the water structure. The first peak for the slab_O-water_O interaction is located at approximately 2.7 Å, a distance slightly shorter than that of the first peak for bulk water_O-water_O, which occurs at about 2.8 Å. This compression indicates that the hydrogen bonds formed directly with the MXene surface are, on average, stronger and more constrained than those in bulk water. A notable trend is observed when comparing surfaces with different hydroxyl concentrations: decreasing the surface hydroxylation from 75% to 50% –OH results in a sharper and more intense first peak in the slab-water RDF. This signifies that a lower density of surface hydroxyl groups promotes a more ordered and well-defined first hydration layer, enhancing the structural coherence of water at the interface.

Regarding the influence of temperature on the O-O pair distance between water molecules, we observe that $Ti_2NT_x(F_0O_{25}OH_{75})$ shows a higher second-layer peak at 4.5 Å at higher temperatures. In contrast, $Ti_3C_2T_x$ and Ti_3CNT_x show minimal change with increasing temperature. As for $Ti_4N_3T_x(F_0O_{25}OH_{75})$, its second peak shifts from 4.25 Å to 4.75 Å as the temperature increases. For the O-O pair distance between water and the MXene oxygen slab, the second O-O peak for the $Ti_4N_3T_x(F_{25}O_{25}OH_{50})$ and $Ti_3CNT_x(F_{50}O_{25}OH_{25})$ cases shifts from 3.8–3.9 Å to 4.0–4.1 Å as temperature increases. We also found that for $Ti_2NT_x(F_0O_{25}OH_{75})$ and $Ti_3C_2T_x(F_0O_{25}OH_{75})$, the slab-water second peak becomes lower as the temperature increases, while the first water layer peak remains almost identical. Therefore, this suggests that the strong, first-layer hydrogen bonding between water and the MXene slab is less susceptible to temperature, while the second-layer structures are affected, resulting in a broader O-O distance peak.

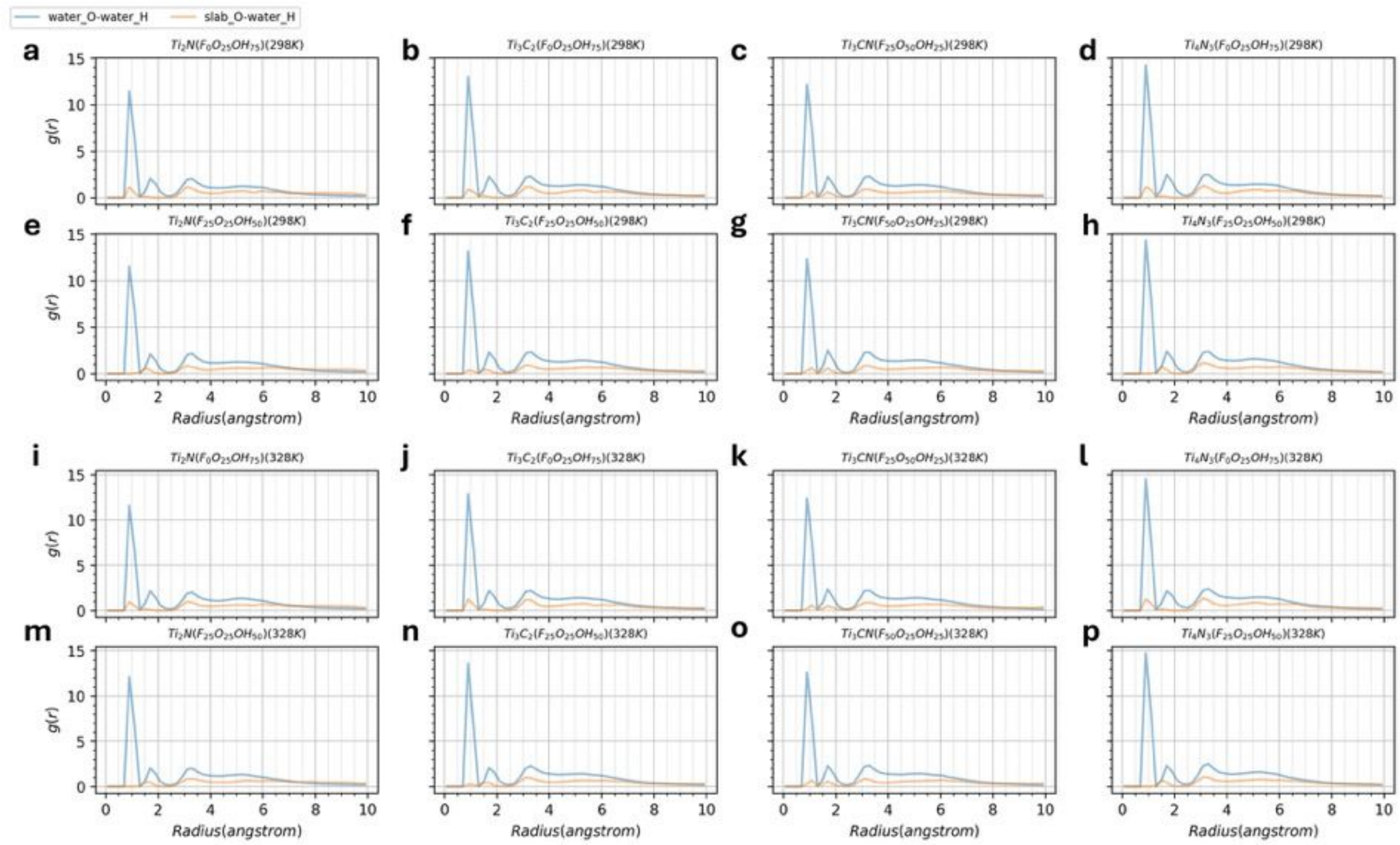

Figure S21. Pairwise O-H radial distribution functions (RDFs), g(r), for various MXene-water systems at 298K (upper) and 328 K (lower). The plots compare the RDF between oxygen and hydrogen atoms within bulk water (water_O-water_H, blue line) and between the surface oxygen atoms of the MXene slab and water hydrogen atoms (slab_O-water_H, orange line). The sharp, intense first peak located at approximately 1.8 Å in all plots is the characteristic signature of hydrogen bonding. Its position indicates the average distance between a hydrogen-bond donor (H) and acceptor (O). Across all systems at either 298K and 328K, the fundamental structure of O-H pair distribution the interfacial hydrogen bonds remains qualitatively similar, with only very slightly broadening behavior nearby second peak at around 3.2 Å when increase temperature.

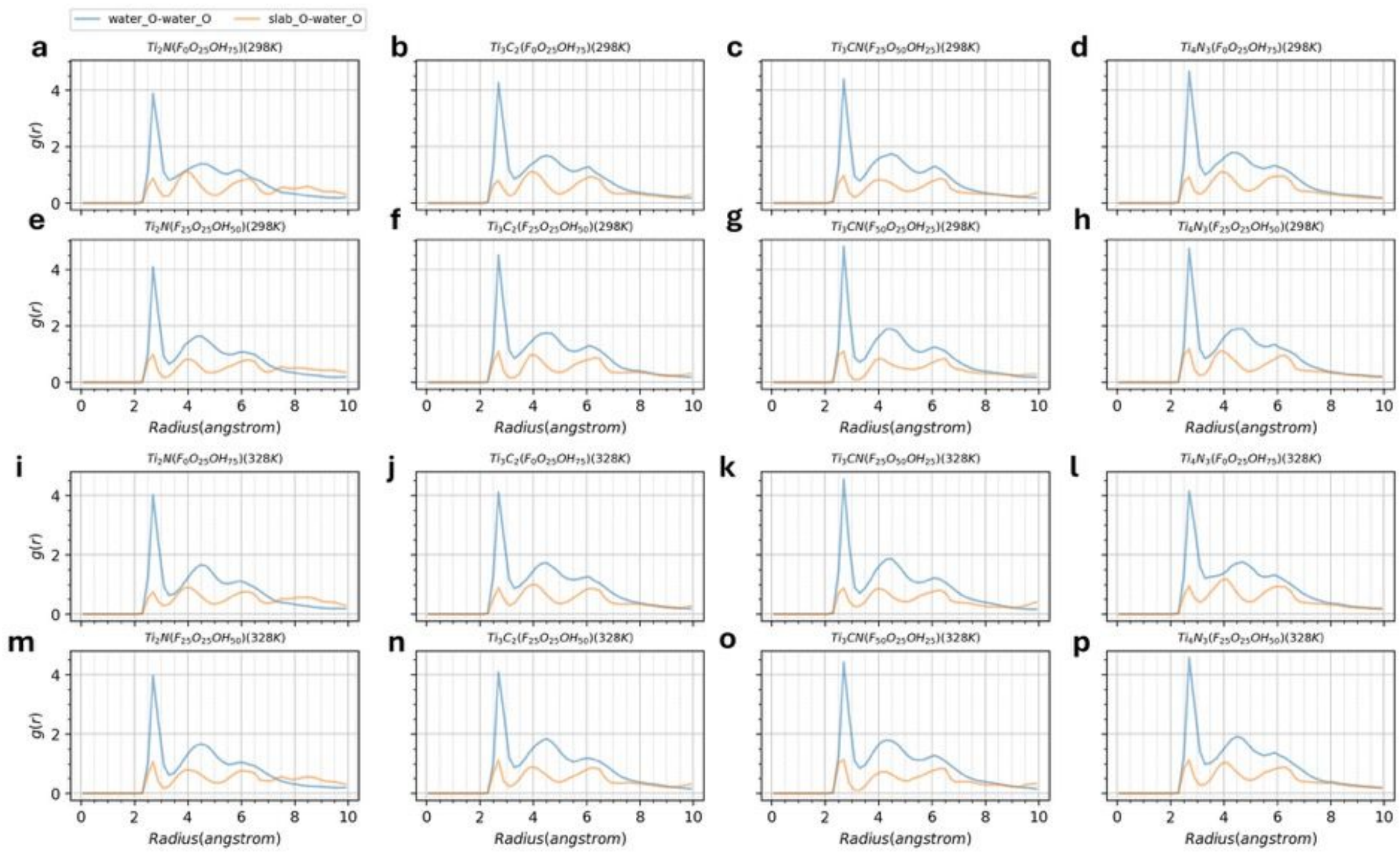

Figure S22. Pairwise oxygen-oxygen radial distribution functions (RDFs), g(r), for different MXene systems at 298K (upper, a-h) and 328 K (lower, i-p). The plots compare the RDF between oxygen atoms on the slab surface and water oxygen atoms (slab_O-water_O, orange) with the RDF between oxygen atoms in bulk water (water_O-water_O, blue). The location of the first peak at 2.7 Å for the slab-water interaction confirms that water molecules form strong hydrogen bonds directly with the -O/-OH functional groups on the MXene surface. This bond distance is slightly shorter than that of bulk water, which has its first sharp peak at approximately 2.8 Å. Comparing the top row (75% OH) to the bottom row (50% OH), the first peak of the slab-water RDFs becomes noticeably sharper and more intense with a slight decrease in surface hydroxylation. A lower concentration of surface hydroxyl groups promotes a more ordered and well-defined first hydration layer, enhancing the structuring of water at the interface.

VII. Discussion 5. MXene Interlayer Water Orientation

The orientational preference of water molecules was quantified by analyzing the probability distribution of cos(θ), where θ is the angle between a specific molecular vector and the surface normal (z-axis) and analyze their FWHM to estimate the population size. Analysis of the H-H vector (**Figure S23**) reveals sharp peaks for interfacial water at small positive cos(θ) values (approximately 0.2–0.4). This indicates a consistent, tilted orientation relative to the surface plane, a stark contrast to the disordered, uniform angular distribution characteristic of bulk water. Analysis of the O-H vector (**Figure S24**) shows a dominant, broad peak near cos(θ)=0, suggesting that a primary orientation involves water molecules lying flat, with their dipole moments parallel to the surface, to act as hydrogen bond acceptors from the surface –OH groups. Smaller, secondary peaks near cos(θ)=±1 indicate that a fraction of O-H vectors also point directly towards or away from the surface, participating as donors to the surface or to the second water layer.

A layer-resolved analysis of the water dipole vector orientation provides the most detailed picture of this structural ordering (**Figure S25** at 298 K and **Figure S26** at 328 K). The results demonstrate that the MXene surfaces acts as platforms imposing a distinct, multi-layered orientational structure that propagates outwards. The first layer of water, in direct contact with the MXene, is highly structured and almost ice-like, with strong orientational peaks around cos(θ)~±0.8, indicating that the molecules are "locked" into a sharply angled configuration to maximize hydrogen bonding with the surface functional groups. Overall, the first water layer dipole moment distribution is bimodal distribution overlapping a trapezoidal, skewed to the right for the bottom surface and to the left for the top surface. There are very few instances where the cosine of the angle, cos(θ), is close to -1 or 1, indicating that few water molecules are aligned perfectly parallel or anti-parallel to the surface normal.

This pronounced ordering decays rapidly with distance. In the middle of the simulated water slab, the orientational distribution becomes uniform, resembling that of disordered bulk water. This templating effect creates a cascade of ordering: the highly structured first layer presents a new, ordered surface to the second layer, which in turn must orient itself to optimize its hydrogen bonding with the first. This propagation of structural information is a indicator of hydrophilic interfaces and is critical for understanding how surface effects can be around 0.5-0.75 Å (two to three water layers) into a liquid. At 328 K, the orientational peaks broaden due to increased thermal

motion, and a complex interplay emerges. For the $F_0O_{25}OH_{75}$ surface on Ti_2NT_x, $Ti_4N_3T_x$, and $Ti_3C_2T_x$, an increase in temperature causes the distribution peak for the bottom surface to shift from -0.7 to -0.5. On the other hand, for the $F_{25}O_{25}OH_{50}$ surface at 328 K, both Ti_2NT_x and $Ti_4N_3T_x$ show more dispersed dipole distributions at both the upper and bottom surfaces. $Ti_3C_2T_x$, however, appears relatively unaffected by temperature changes for first water layers. Even though the first water layer on the $Ti_3C_2T_x(F_0O_{25}OH_{75})$ surface shows no significant change, its corresponding second water layer still displays a broader and more dispersed orientational behavior. On the $F_{25}O_{25}OH_{50}$ surface of Ti_3CNT_x, the second water layer exhibits a flattened orientation. As the temperature or the concentration of fluoride (–F) groups increases, the water molecules in this second layer become more oriented, with their dipole moment pointing up and down. In general, increasing either the temperature or the –F concentration promotes a flattened orientation for water molecules. This occurs because fewer flat-lying water molecules in the first layer allow the connected second layer to become more ordered (oriented up and down). Conversely, a highly oriented first water layer induces a more flattened orientation in the corresponding second layer. From the observations together, a slight change on highly ordered first layer tends to induce a more flattened, disordered second layer, whereas a less ordered first layer can permit the second layer to adopt a more structured, up-and-down orientation.

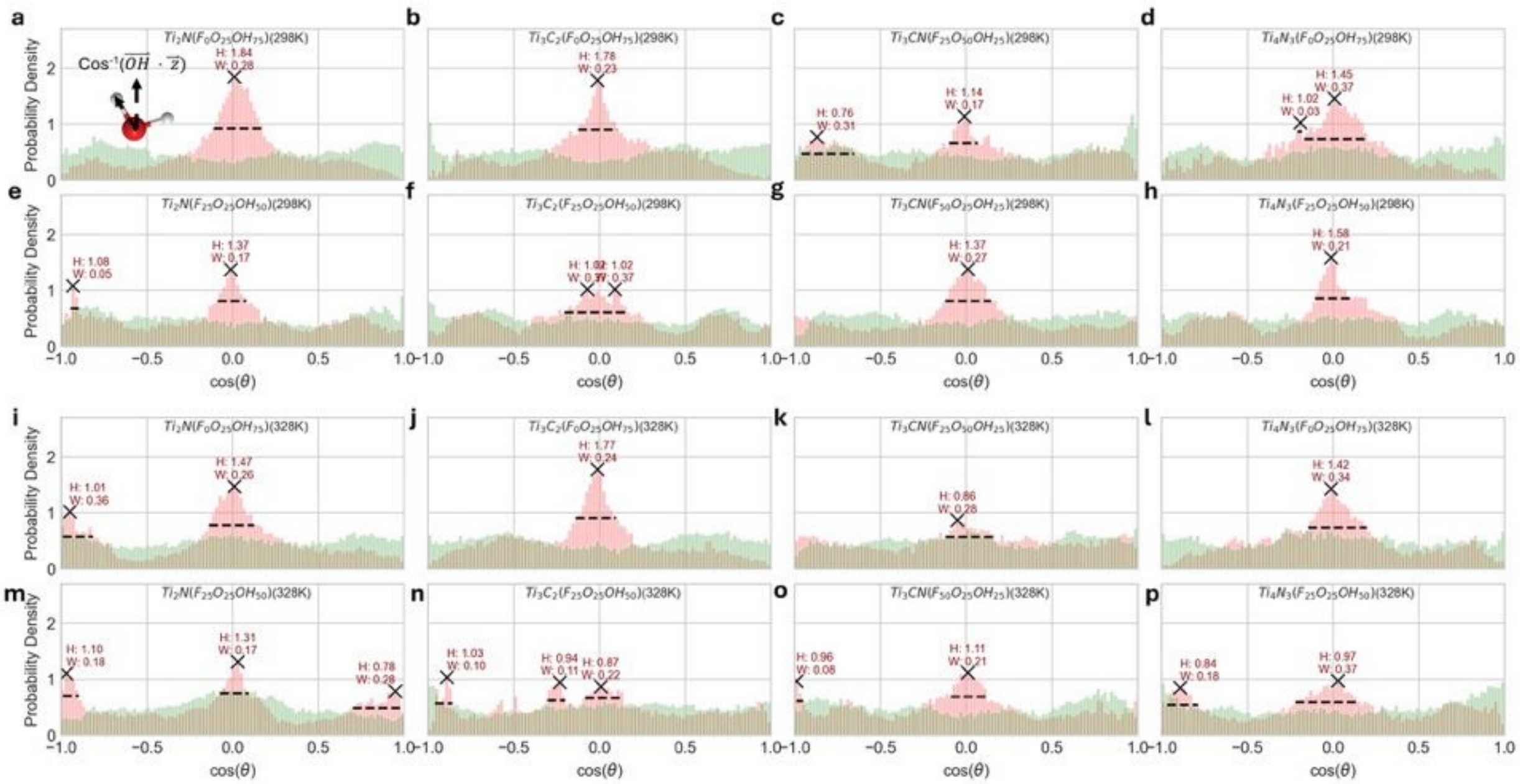

Figure S23. Comparative analysis of water molecule orientation at the interface of eight different functionalized MXene surfaces at 298 K and 328K. The figure displays the probability density distribution of cos(θ), where θ is the angle between the H-H vector of a water molecule and the z-axis (surface normal). The similar H-H vectors representation are illustrated in Figure 3d and 3e. The pink and green histograms correspond to the water molecules in the first hydration layer at MXene and background distribution of all other water molecules in the system, respectfully. Each panel is labeled with the specific chemical composition of the MXene. The peak height (H) and Full Width at Half Maximum (FWHM, W) further quantify this ordering, with taller, narrower peaks indicating a more uniform and strongly held molecular arrangement. This metric reveals the water molecules' preferred pose: cos(θ) ~ 1 or cos(θ) ~ -1 corresponds to a water molecule "standing up" with its H-H vector perpendicular to the surface, while cos(θ) = 0 corresponds to it "lying flat." The sharp peaks for interfacial water (pink) signify a highly ordered structure imposed by the MXene, contrasting with the disordered, uniform distribution of bulk water (green). Notably, the peaks for all surfaces appear at small positive cos(θ) values (e.g., ~0.2-0.4), revealing a consistent, tilted orientation as the most stable configuration.

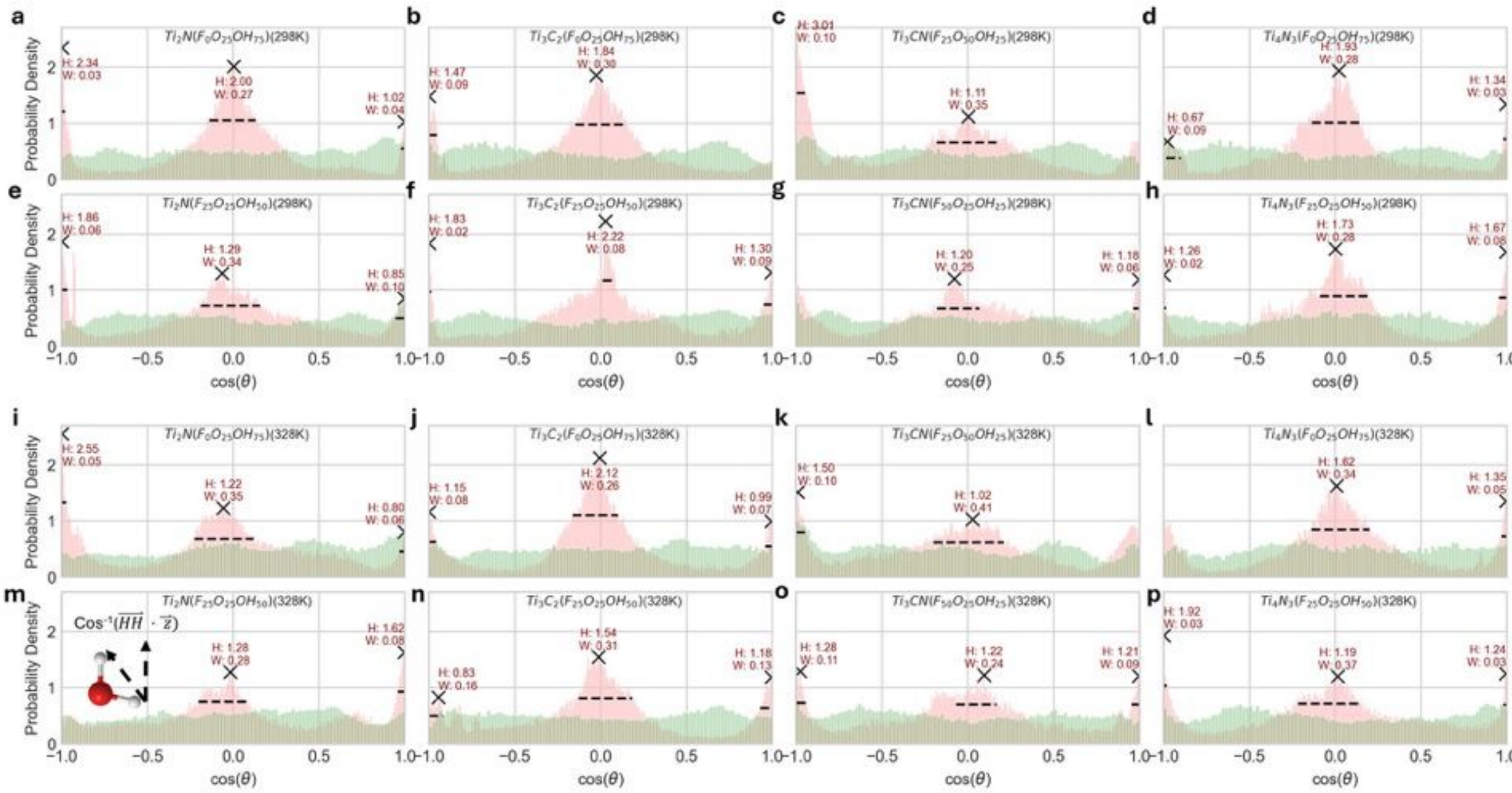

Figure S24. Comparative analysis of water molecule orientation at the interface of eight different functionalized MXene surfaces at 298 K and 328K. The figure displays the probability density distribution of cos(θ), where θ is the angle between the O-H vector of a water molecule and the z-axis (surface normal). The similar H-H vectors representation are illustrated in Figure 3b and 3c. The pink and green histograms correspond to the water molecules in the first hydration layer at MXene and background distribution of all other water molecules in the system, respectfully. Each panel is labeled with the specific chemical composition of the MXene. The peak height (H) and Full Width at Half Maximum (FWHM, W) further quantify this ordering, with taller, narrower peaks indicating a more uniform and strongly held molecular arrangement. This metric reveals the preferred pose of the water molecules: values of cos(θ)~1 and cos(θ)~−1 correspond to a molecule "standing up" with its O-H vector pointing perpendicularly up and down, respectively, while cos(θ)=0 corresponds to the O-H vector "lying flat." The sharp peaks for interfacial water (pink) signify a highly ordered structure imposed by the MXene, contrasting with the disordered, uniform distribution of bulk water (green). Notably, all surfaces show a strong, broad peak near cos(θ)=0, suggesting that water near the MXene serves as a hydrogen bond acceptor in a flat orientation. Some surfaces also show a peak between cos(θ)=−0.8 ~ −1.0, and cos(θ)=0.8 ~ 1.0,. This indicates that near water-Mxene surfaces, some portion of water's O-H vectors prefer to point down and point up.

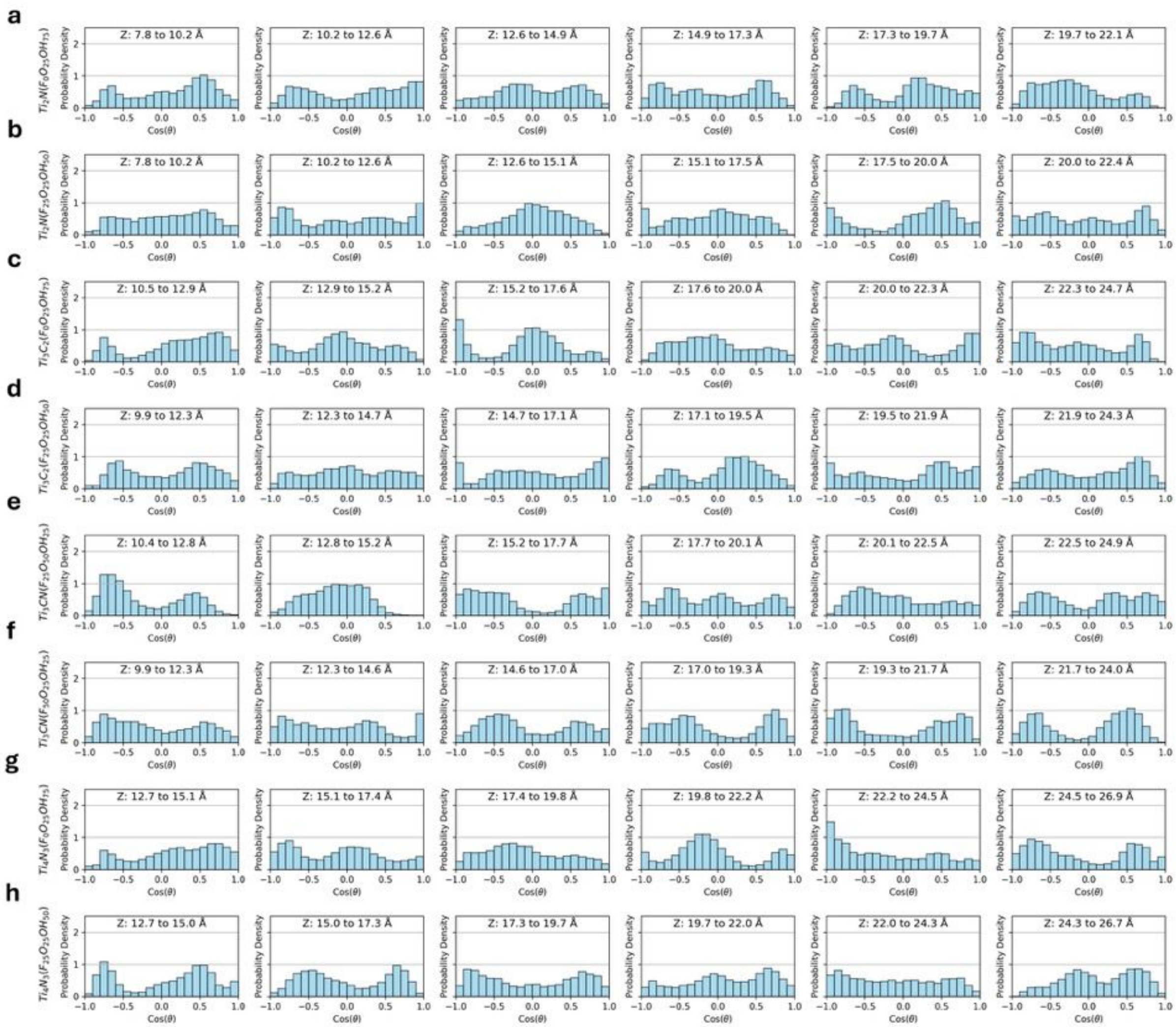

Figure S25. Spatial analysis of water molecule orientation at various functionalized MXene interfaces from AIMD. All populations were collected and averaged for simulation times greater than 4000 fs at 298 K. The orientation is measured using Cos(θ), where θ is the angle between the water dipole vector and the surface normal (Z-axis). A value of Cos(θ) = 1 or -1 indicates an orientation perpendicular to the surface, while Cos(θ) = 0 indicates a parallel orientation. Definition of the first through sixth water hydration layers along the z-axis are shown as in Figure 3a. Noted that the upper and lower bounds vary depending on the specific system. In a confined space between functionalized MXene sheets, water molecules behave oriented. The chemically active surface of the MXene creates a strong electrostatic field with specific "docking sites" that force the water molecules into preferred alignments. The layer of water directly touching the MXene sheet, known as the interfacial layer, is highly structured and almost ice-like. Instead of random tumbling, the molecules are "locked" into position, and depend on surface chemistry of the MXene, functional groups type and percentage. The data shows strong peaks around Cos(θ) ~ ±0.8, indicating that most molecules are sharply angled either toward or away from the surface. This specific orientation allows them to form strong hydrogen bonds with the oxygen, fluorine, or

hydroxyl groups on the MXene. As moving away from this initial layer into the middle of the channel, the water begins to behave like normal "bulk" water. The sharp orientational peaks disappear, and the molecules become disordered, interacting primarily with each other rather than the MXene walls. However, as the water approaches the top MXene sheet, this structured, ordered state re-emerges as a mirror image of the bottom layer.

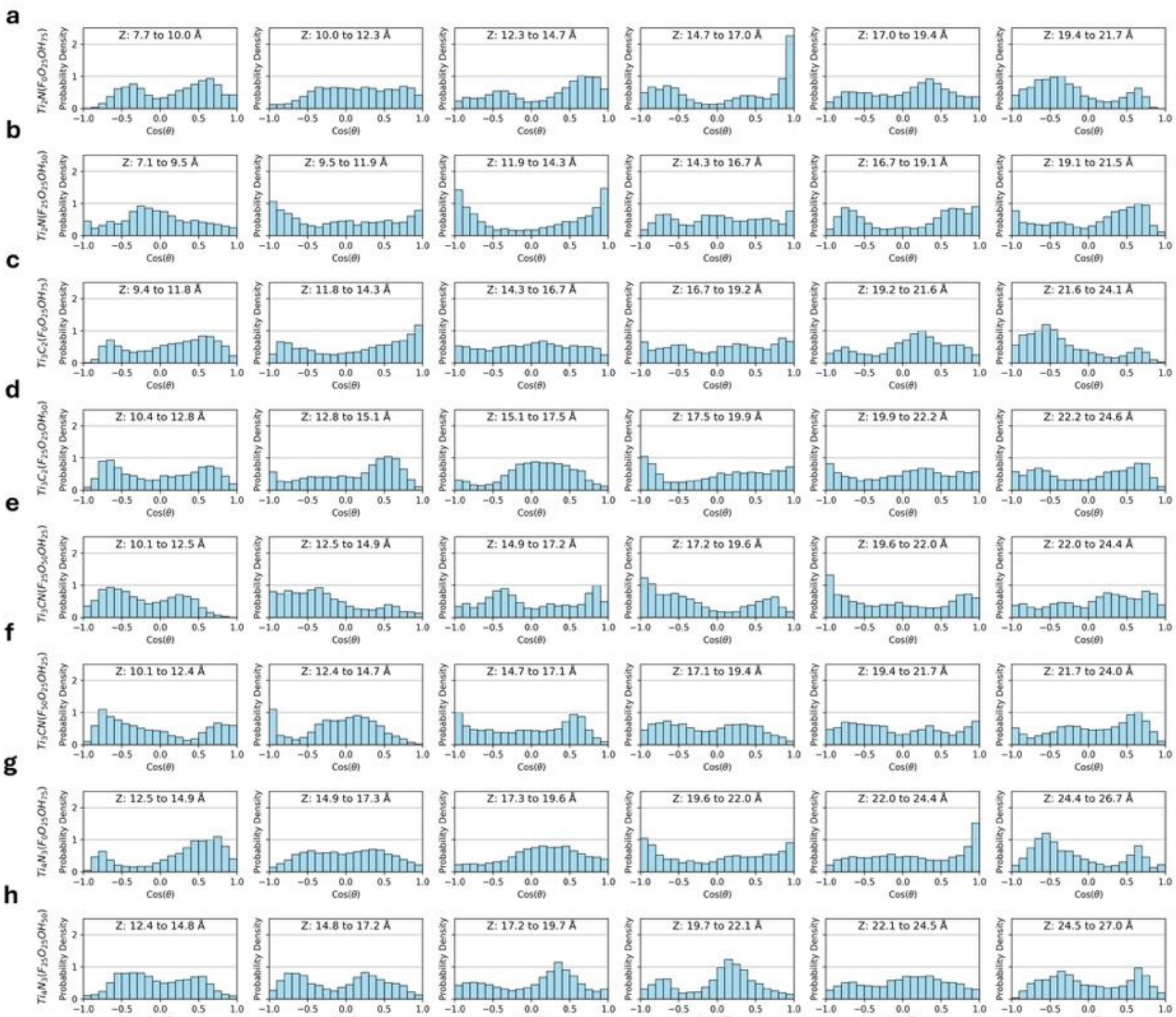

Figure S26. Spatial analysis of water molecule orientation at various functionalized MXene interfaces from AIMD. Definition of the first through sixth water hydration layers along the z-axis are shown as Figure S15 and Figure 3a. Noted that the upper and lower bounds vary depending on the specific system. All probability density was collected and average for simulation times greater than 4000 fs at 328 K over one sixth of water layer regions. All the axis and notations are described as **Figure S15**. As the temperature increases from 298 K to 328 K, the orientation peak for the first water layer broadens slightly. In contrast, the second water layer shows a much broader orientation profile. This indicates that water molecules in the second layer are less parallel to the surface and are more likely to be oriented perpendicular to it, with some waters molecules cos(θ) values approaching -1 or 1, suggesting a increase in interlayer hydrogen bonding formation.

VIII. Discussion 6. Hydrogen Bond Lifetimes and Stability

To quantify the persistence of hydrogen bonds, we calculated their time autocorrelation functions, C(τ). A slower decay of C(τ) corresponds to a longer average hydrogen bond lifetime and thus a more stable interaction. The results are presented for medium-strong HBs (2.9 Å cutoff) in **Figure S27** and for strong HBs (2.7 Å cutoff) in **Figure S29**. A consistent finding across all systems and temperatures is that the decay rate for slab-water HBs (orange lines) is significantly slower than that for water-water HBs (blue lines). This directly demonstrates that the hydrogen bonds formed between water and the MXene surface are more stable and longer-lived than those within the interlayer bulk liquid, confirming the strong anchoring effect of the functionalized surface.

The influence of surface chemistry on these dynamics is complex and multifaceted. For instance, increasing the –F termination from 0% to 25% on several MXene types appears to increase the lifetime of the remaining slab-water HBs, again supporting the model where –F atoms create isolated, deep potential wells for H-bonding that trap water molecules for longer durations. To facilitate a direct quantitative comparison, the decay intercepts of the autocorrelation functions at a fixed time lag of τ=0.3 ps are summarized in Figure S18a-d (for 2.7 Å HBs) and Figure S18e-h (for 2.9 Å HBs).

Medium-Strong Hydrogen Bond Dynamics (Distance < 2.9 Å)

When analyzing the broader category of medium-strong hydrogen bonds, the role of fluorine in organizing the interfacial water layer is effect at 298K, but appears less effective at higher temperatures. At 298 K, the pristine hydroxylated surface of $Ti_4N_3T_x(F_0O_{25}OH_{75})$ initially exhibits a longer HB lifetime than its Ti_2NT_x and $Ti_3C_2T_x$ counterparts, an advantage that diminishes upon fluorination or heating. Counterintuitively, a modest increase in -F concentration at 298 K was observed to slightly enhance the HB lifetime for Ti_2NT_x, $Ti_3C_2T_x$, and Ti_3CNT_x systems, suggesting more stable Medium-Strong Hydrogen after introducing -F. The influence of temperature, however, reveals critical distinctions in thermal stability. Upon heating from 298 K to 328 K, all hydroxyl-terminated systems ($F_0O_{25}OH_{75}$) experience a decay in HB lifetime. For fluorinated systems, while Ti_2NT_x, $Ti_3C_2T_x$, and $Ti_4N_3T_x$ also show decay, the Ti_3CNT_x surfaces ($F_{25}O_{50}OH_{25}$ and $F_{50}O_{25}OH_{25}$) are a remarkable exception. The medium-strong HB lifetime on these Ti_3CNT_x surfaces remains almost entirely unaffected by the temperature increase. This exceptional stability indicates that a sufficient concentration of fluorine on the Ti_3CNT_x surface

can effectively pushed the first water layer to nearby -OH termination, creating a interfacial water network structures that is uniquely resistant to temperature fluctuations.

Strong Hydrogen Bond Dynamics (Distance < 2.7 Å)

The stability of strong hydrogen bonds (HBs) at the MXene-water interface is governed by a complex interplay between surface functionalization, substrate identity, and temperature. On the Ti_2NT_x surface at 298 K, a higher hydroxyl concentration ($F_0O_{25}OH_{75}$) promotes a more stable HB network, indicated by a decay intercept of 0.22, compared to a lower concentration ($F_{25}O_{25}OH_{50}$) which yields a value of 0.18. While fluorine (-F) functionalization generally appears to diminish HB stability, notable exceptions exist. For instance, the Ti_3CNT_x material at 328 K shows that a surface with 50% fluorine ($F_{50}O_{25}OH_{25}$) possesses greater HB stability (0.25) than one with 25% fluorine ($F_{25}O_{50}OH_{25}$) (0.21). The most striking finding, however, is the substrate-dependent effect of fluorination. At 298 K, in the absence of fluorine, the order of HB stability is $Ti_3C_2T_x$ (0.29) > Ti_2NT_x (0.22) > $Ti_4N_3T_x$ (0.18). The introduction of just 25% -F dramatically inverts this trend to $Ti_4N_3T_x$ (0.19) > Ti_2NT_x (0.18) > $Ti_3C_2T_x$ (0.12), demonstrating that the influence of a functional group is not intrinsic but is instead modulated by the electronic and structural properties of the underlying MXene substrate. Furthermore, at elevated temperatures, the presence of -F significantly accelerates the decay of strong HB lifetimes for Ti_2NT_x and $Ti_3C_2T_x$, whereas $Ti_4N_3T_x$ and Ti_3CNT_x exhibit much greater resilience. This suggests that the $Ti_4N_3T_x$ and Ti_3CNT_x MXenes, while potentially forming fewer strong HBs, maintain a more persistent and thermally robust hydrogen-bonding network.

Overall, we believe the electronic structure of the underlying MXene slab (carbide vs. nitride) interacts with the charge-withdrawing fluorine atoms differently, which in turn alters the H-bonding capability of the adjacent –O and –OH sites. This demonstrates a synergistic or, in some cases, antagonistic coupling between the substrate electronics and the surface chemistry that ultimately governs the dynamics of the interfacial water. As expected, increasing temperature generally decreases hydrogen bond stability. However, the strong HBs on the high-fluorine Ti_3CNT_x surface are insensitive to temperature changes, suggesting the -F surface group stabilizes the water structure.

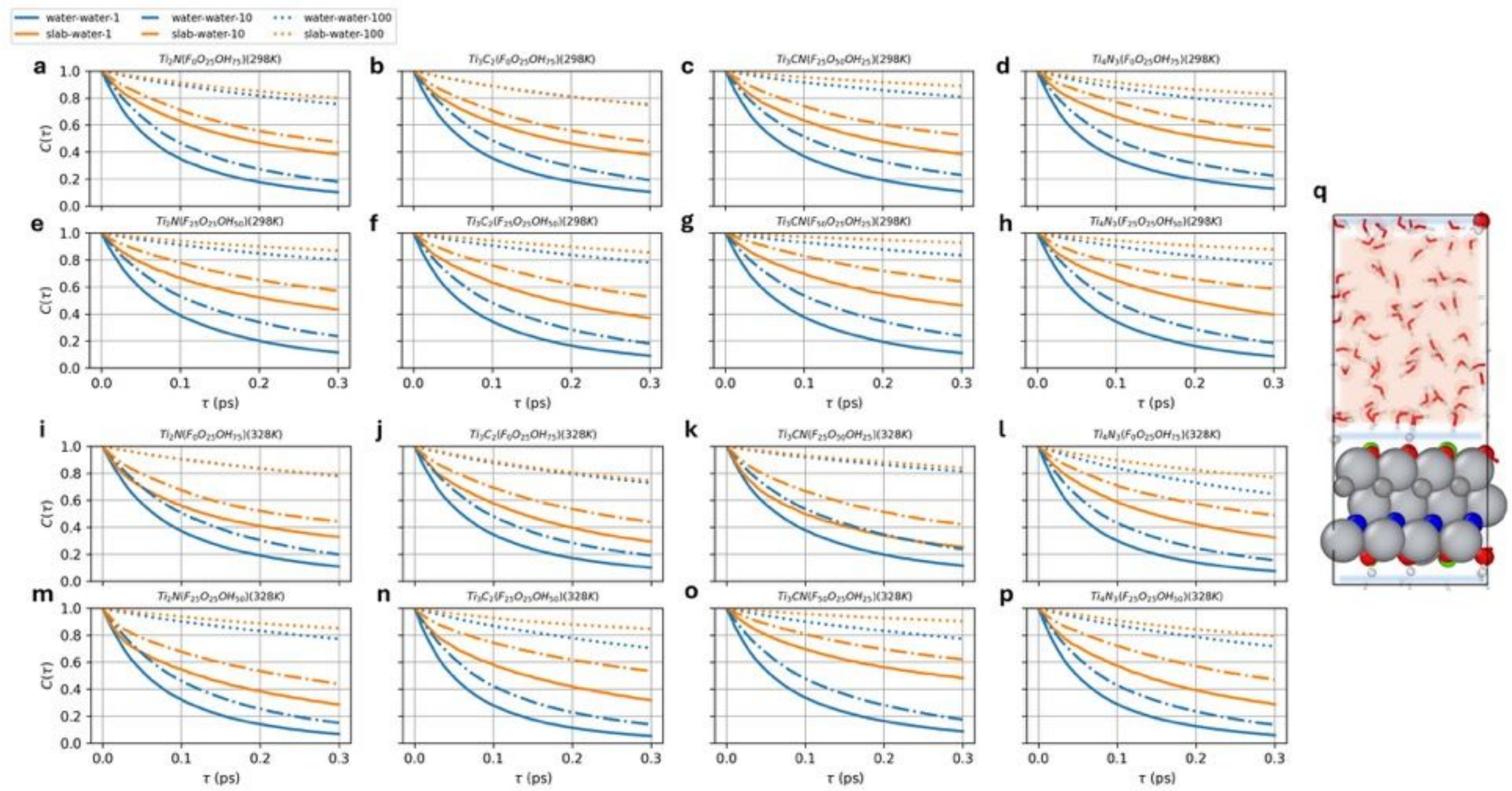

Figure S27. Hydrogen bond autocorrelation functions, C(τ), comparing the dynamics of water-water (blue lines) and slab-water (orange lines) medium-strong to strong hydrogen bonding with 2.9 Å cutoff at 298 K (upper subplots, a-h) and 328K (lower subplots, i-p). The solid, dash-dotted, and dotted lines correspond to intermittency of $C_I(t) = 1$ ($=C_c(t)$), 10, 100 fs respectively. The plots compare hydrogen bonds formed between water molecules (water-water, blue) with those formed between the slab surface and a water molecule (slab-water, orange). q) Cartoon illustration snapshot of water-water and slab-water hydrogen bonding region. Hydrogen bonding between "slab-water" only included the hydrogen bonding between terminations of surface and water molecules, while "water-water" include all other hydrogen bonding that ever form. For example, water-water-10 represents hydrogen bonds formed between water molecules with $C_I(t)$=10 fs intermittency. The top rows correspond to surfaces with lower -F terminations. ($F_0O_{25}OH_{75}$ for Ti_2NT_x, $Ti_3C_2T_x$, $Ti_4N_3T_x$ and $F_{25}O_{50}OH_{25}$ for Ti_3CNT_x), and bottom rows correspond to relative higher -F terminations ($F_{25}O_{25}OH_{50}$ for Ti_2NT_x, $Ti_3C_2T_x$, $Ti_4N_3T_x$ and $F_{50}O_{25}OH_{25}$ for Ti_3CNT_x). Overall, the slower decay rates for the slab-water interactions indicate more stable and longer-lived hydrogen bonds at the solid-liquid interface. As -F termination increases from 0% to 25%, longer HB lifetime of MXene-water observed. The decay intercept (τ=0.3ps) value are recorded in **Figure S28e-h**.

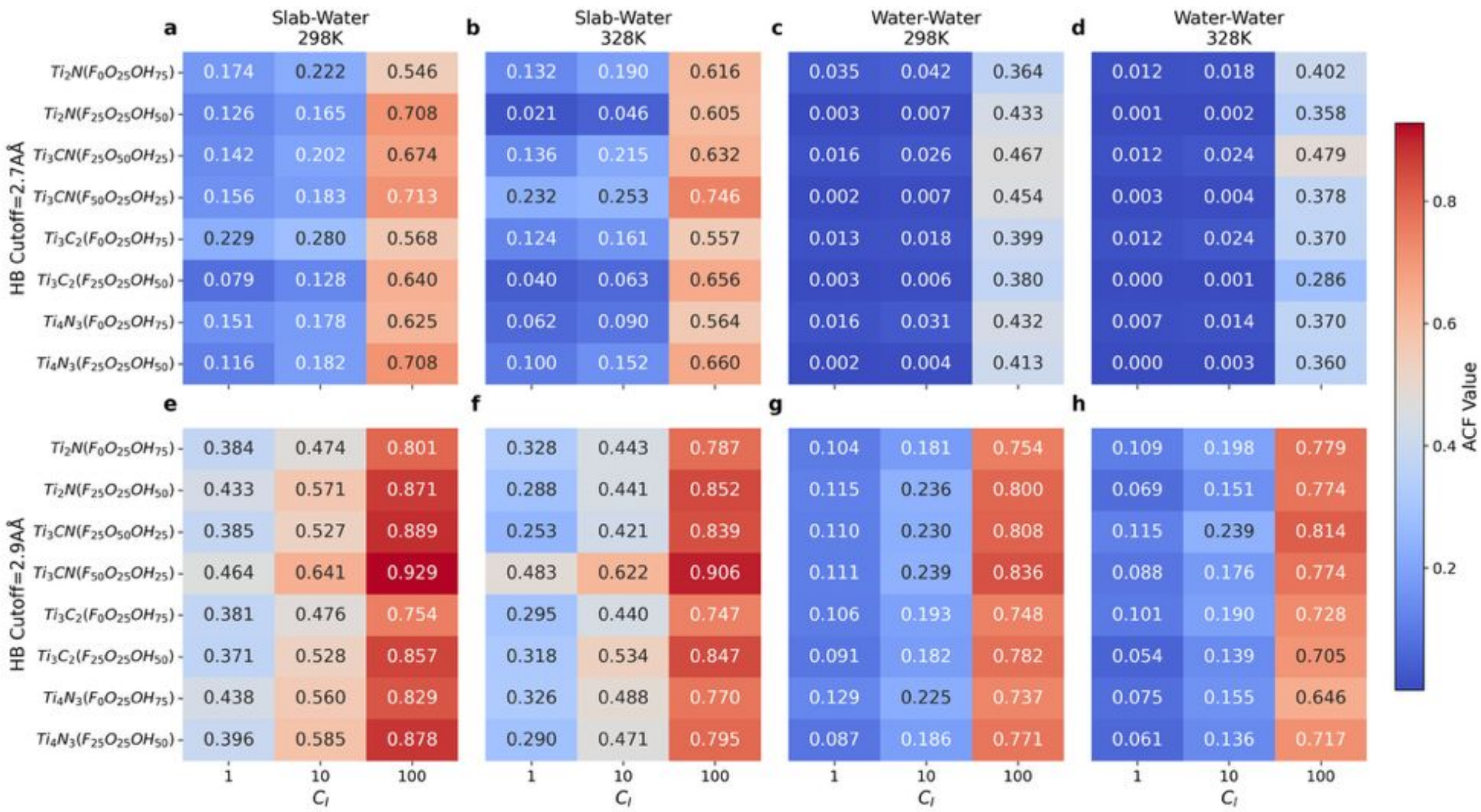

Figure S28. A summary of decay intercepts heatmap table for the hydrogen bond autocorrelation function, recorded ACF at a fixed time lag of τ=0.3 ps to assess interaction stability for strong hydrogen bonding with 2.7 Å HB cutoff (a-d) and for medium-strong to strong hydrogen bonding with 2.9Å HB cutoff (e-h). The table compares ACF values for four different MXene materials (Ti_2NT_x, $Ti_3C_2T_x$, $Ti_4N_3T_x$ and Ti_3CNT_x) under different temperatures and surface compositions. The values for each case correspond to the intermittent autocorrelation function calculated with an intermittency C_I=1, C_I=10 and C_I=100. Overall, for strong HB with 2.7 Å cutoff (a-d), the two values for each case correspond to the intermittent autocorrelation function calculated with an intermittency CI=1 and CI=10. Increasing the temperature from 298K to 328K generally decreases the stability of strong hydrogen bonds. This is evident across almost all MXene types and surface compositions. And overall higher -OH Content boosts connectivity of strong HB. However, the strong hydrogen bond decay rate of Ti_3CNT_x MXene shown lesser affected by -F concentration and temperature with higher -F surface terminations. As for 2.9 Å HB cutoff (e-h), the hydrogen bond (HB) lifetime of Ti_2NT_x and $Ti_3C_2T_x$ is similar, while $Ti_4N_3T_x$ shows a longer lifetime with ($F_0O_{25}OH_{75}$) functionalization at 298K compared to the other MXenes, but this behavior disappears after increase -F or temperature. At 298K, increasing the -F concentration from ($F_0O_{25}OH_{75}$) to ($F_{25}O_{25}OH_{50}$) increases the HB lifetime of Ti_2NT_x and $Ti_3C_2T_x$. A similar trend occurred for Ti_3CNT_x when changing from ($F_{25}O_{50}OH_{25}$) to ($F_{50}O_{25}OH_{25}$), however, the HB lifetime increased only very slightly. For Temperature change, as the temperature increases from 298K to 328K for the ($F_0O_{25}OH_{75}$) systems, the HB lifetime decays faster for all of them, where Ti_2NT_x shows the smallest but still obvious decrease. When the temperature increases over the same range for the ($F_{25}O_{25}OH_{50}$) system, the HB lifetime decays for Ti_2NT_x, $Ti_3C_2T_x$, and $Ti_4N_3T_x$. However, for Ti_3CNT_x with ($F_{25}O_{50}OH_{25}$) and ($F_{50}O_{25}OH_{25}$) functionalization, the change is

almost negligible. This indicates that when the -F concentration is high enough, the first water layer tends to stay fixated with the same -OH group and is less influenced by temperature fluctuations.

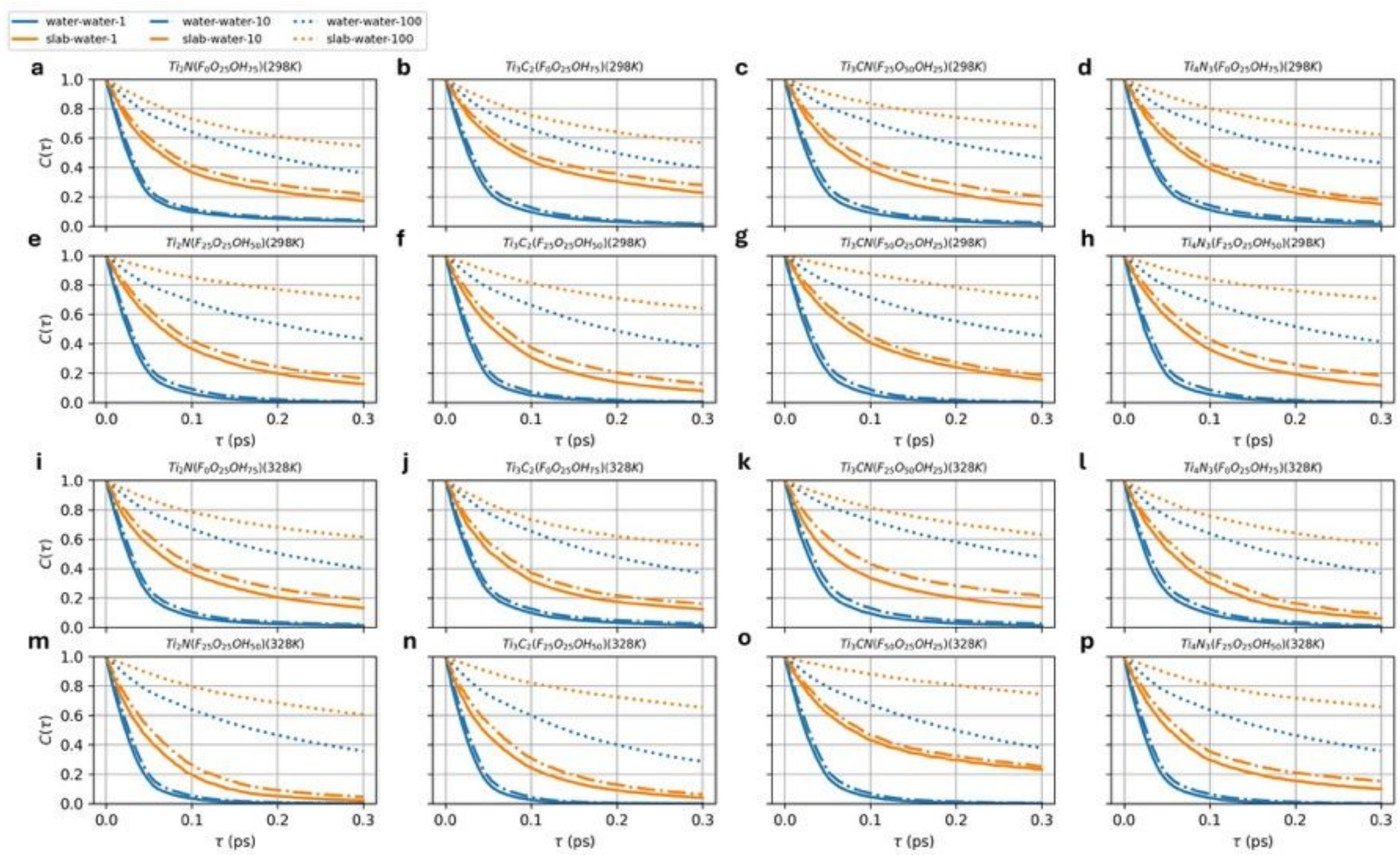

Figure S29. Hydrogen bond autocorrelation functions, C(τ), comparing the dynamics of water-water (blue lines) and slab-water (orange lines) of strong hydrogen bonding with 2.7 Å cutoff at 298 K (upper subplots) and 328K (lower subplots). The solid, dash-dotted, and dotted lines correspond to intermittency of $C_I(t) = 1$ ($=C_c(t)$), 10, 100 fs respectively. The plots compare hydrogen bonds formed between water molecules (water-water, blue) with those formed between the slab surface and a water molecule (slab-water, orange). The legends style and detail explanations are as shown S17 and S17q. The top rows correspond to surfaces with lower -F terminations. ($F_0O_{25}OH_{75}$ for Ti_2NT_x, $Ti_3C_2T_x$, $Ti_4N_3T_x$ and $F_{25}O_{50}OH_{25}$ for Ti_3CNT_x), and bottom rows correspond to relative higher -F terminations ($F_{25}O_{25}OH_{50}$ for Ti_2NT_x, $Ti_3C_2T_x$, $Ti_4N_3T_x$ and $F_{50}O_{25}OH_{25}$ for Ti_3CNT_x). MXene materials show distinct responses to thermal fluctuations. The strongest hydrogen bonding occurs at 298 K for $Ti_3C_2T_x$ ($F_0O_{25}OH_{75}$) but at a higher temperature of 328 K for Ti_3CNT_x ($F_{50}O_{25}OH_{25}$), highlighting the differing influence of the material and its functional groups.

IX. Discussion 7. Layer-Resolved Hydrogen Bond Dynamics

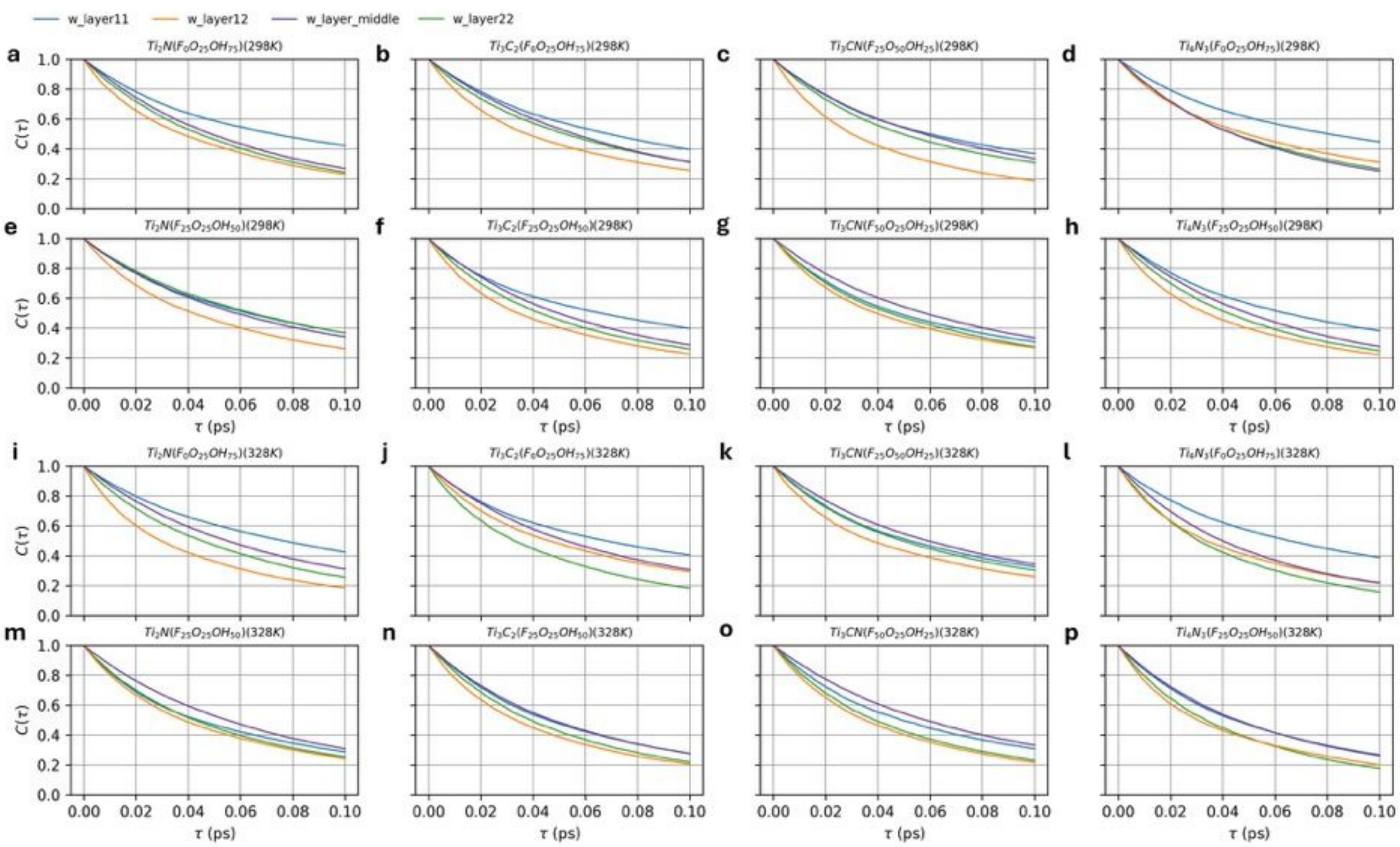

Figure S30. Continuous time autocorrelation function, $C_c(\tau)$, for hydrogen bonds in different water regions near various functional MXene surfaces at 298 K (upper subplots), 328 K (lower subplots). The functions are calculated for medium-strong to strong HBs (2.9Å cutoff) formed exclusively within the first water layer (w_layer11), between the first and second water layers excluding within layers themselves (w_layer12), exclusively within the second water layer (w_layer22), and among all water molecules excluding the first two layers (w_layer_middle). The layer representation legends are shown as **Figure 7i** illustration. Overall, HB within the first water layer show longer lifetime, and HB between the first water layer and the second water layer is the weakest and decay faster. Interestingly, HB within the first water layer (w_layer11) of $Ti_2NT_x(F_0O_{25}OH_{75})$ and $Ti_4N_3T_x(F_0O_{25}OH_{75})$ show much longer lifetime than all other layres and $Ti_3C_2T_x(F_0O_{25}OH_{75})$ show slightly longer lifetime. However, as increase of -F coverage ratio and temperature, we observed the trend disappeared and the HB lifetime become very similar to the interlayer middle water layers (w_layer_middle).

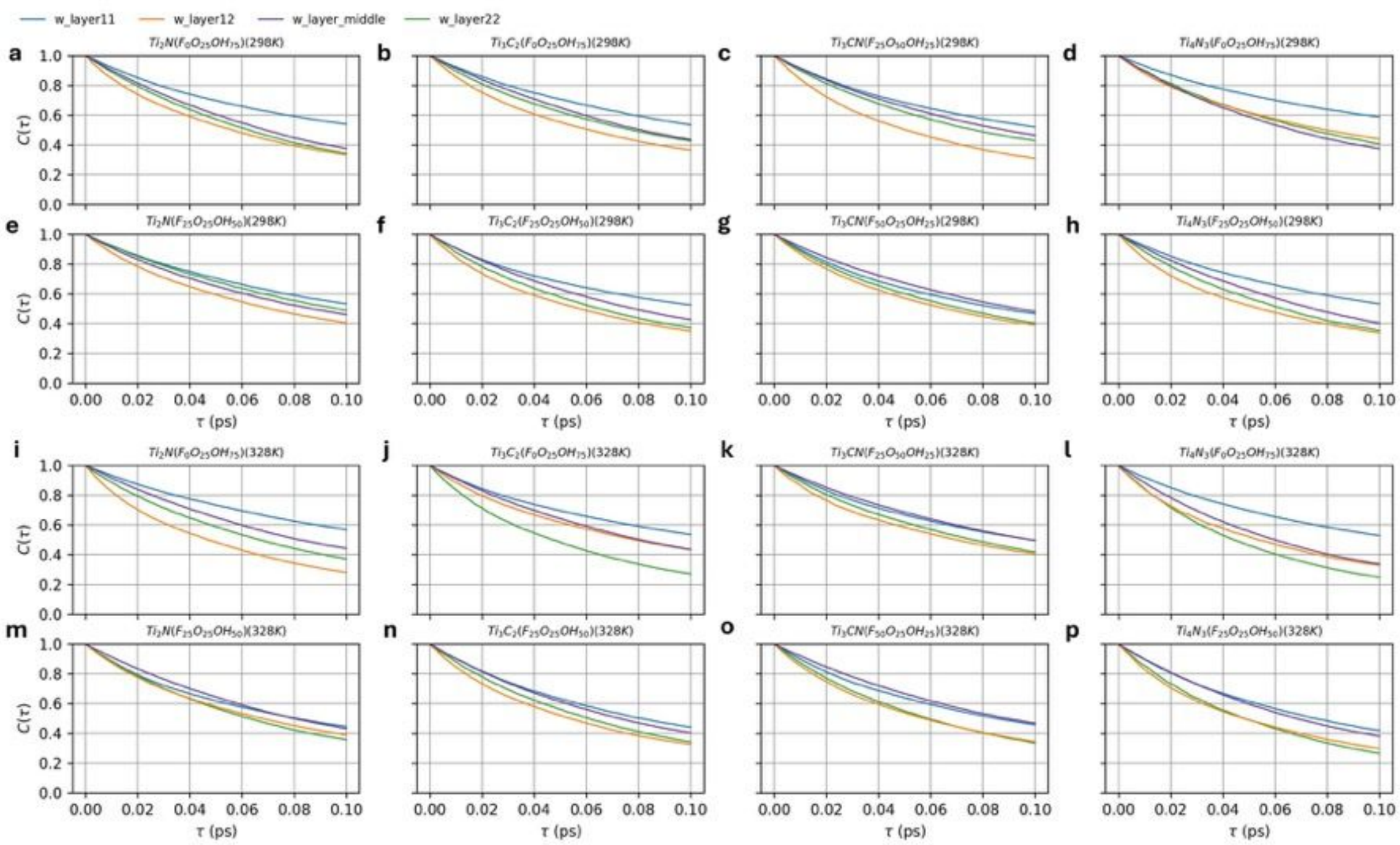

Figure S31. Intermittent time autocorrelation function, $C_I(\tau)$ =10 fs, for hydrogen bonds in different water regions near various functional MXene surfaces at 298 K (upper subplots), 328 K (lower subplots). The functions are calculated for medium-strong to strong HBs (2.9Å cutoff) formed exclusively within the first water layer (w_layer11), between the first and second water layers excluding within layers themselves (w_layer12), exclusively within the second water layer (w_layer22), and among all water molecules excluding the first two layers (w_layer_middle). The layer representation legends are shown as **Figure 7i** illustration. Overall the trend and the lifetimes order are very similar to the $C_t(\tau)$ = 1 fs case. Increasing the temperature from 298K to 328K reveals distinct effects on the stability of especially second water layers, which are dependent on the MXene's composition and surface termination. For both $Ti_3C_2T_x(F_0O_{25}OH_{75})$ and $Ti_4N_3T_x(F_0O_{25}OH_{75})$, the first water layer's lifetime remains largely unaffected, while the second layer's lifetime shortens, indicating it becomes more mobile and disordered at higher temperatures. This similar effect is also pronounced on the high-hydroxyl surface of $Ti_2NT_x(F_0O_{25}OH_{75})$, where thermal fluctuations cause the first water layer's lifetime to decay significantly faster than on other surfaces. Although this layer maintains stable hydrogen bonds with the MXene surface, its interaction with the increasingly mobile second layer weakens. Conversely, as the concentration of surface hydroxyl groups decreases, the rapid decay of the second layer becomes less sensitive to temperature changes, highlighting the critical role of -OH and -F on MXene surface chemistry in mediating the thermal stability of interfacial water structures.

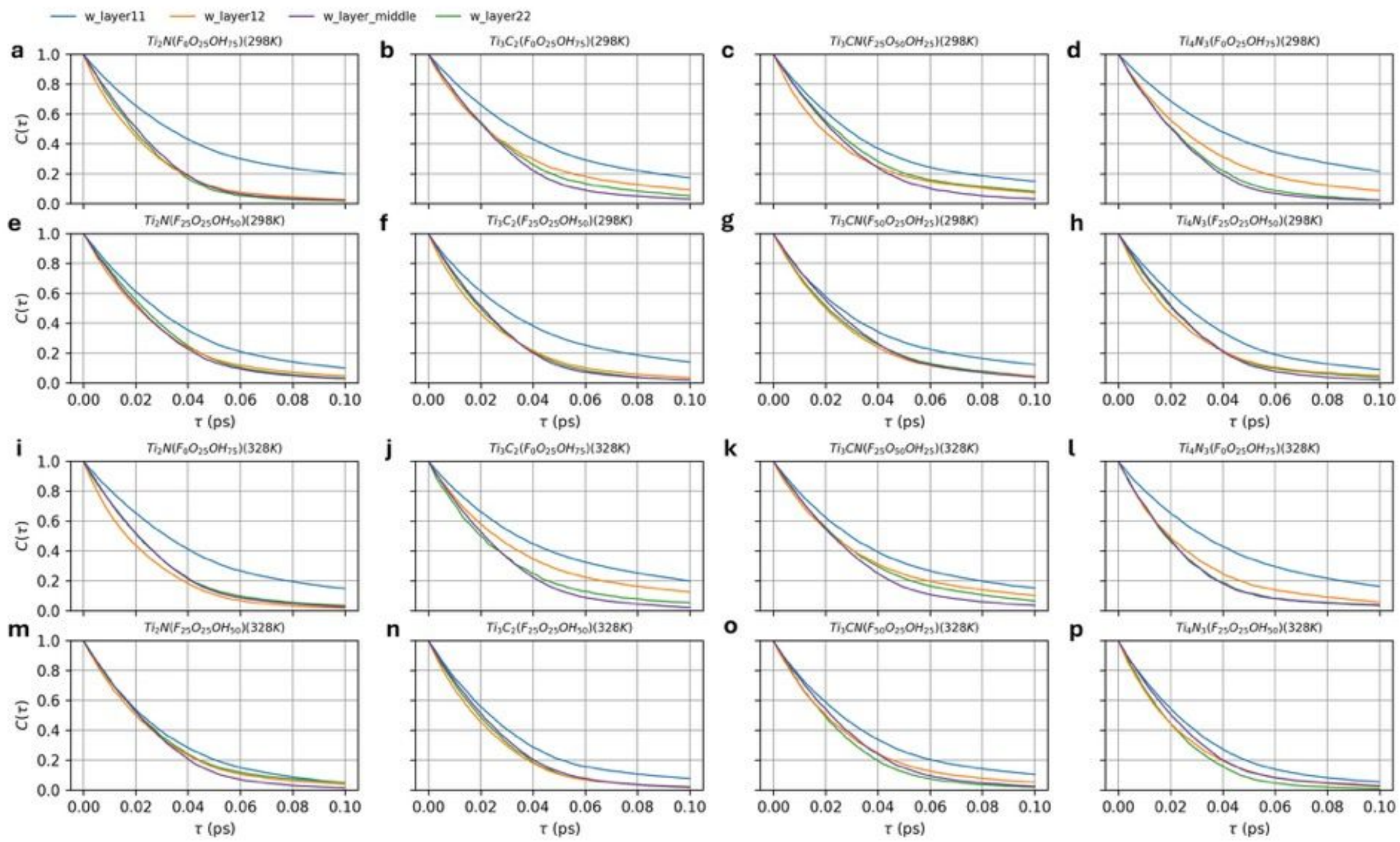

Figure S32. Continuous time autocorrelation function, $C_I(\tau)$ =1 fs, for hydrogen bonds in different water regions near various functional MXene surfaces at 298 K (upper subplots), 328 K (lower subplots). The functions are calculated for strong HBs (2.7Å cutoff) formed exclusively within the first water layer (w_layer11), between the first and second water layers excluding within layers themselves (w_layer12), exclusively within the second water layer (w_layer22), and among all water molecules excluding the first two layers (w_layer_middle). The layer representation legends are shown as **Figure 7i** illustration. A key observation is that HBs within the first water layer (w_layer11) consistently exhibit the slowest decay for all systems and temperatures. The -F surface termination significantly influences the HB dynamics between the first and second water layers (w_layer12). Specifically, in the absence of -F termination, the $Ti_3C_2T_x$ and $Ti_4N_3T_x$ systems show more stable and slower-decaying HBs in this region at both temperatures. This effect is less pronounced for the Ti_2NT_x and Ti_3CNT_x systems. Overall, strong continuous HBs decay rapidly across all systems as higher temperatures, an effect that is particularly notable for the nitride-based MXenes, whereas the $Ti_3C_2T_x$ system shows greater thermal stability.

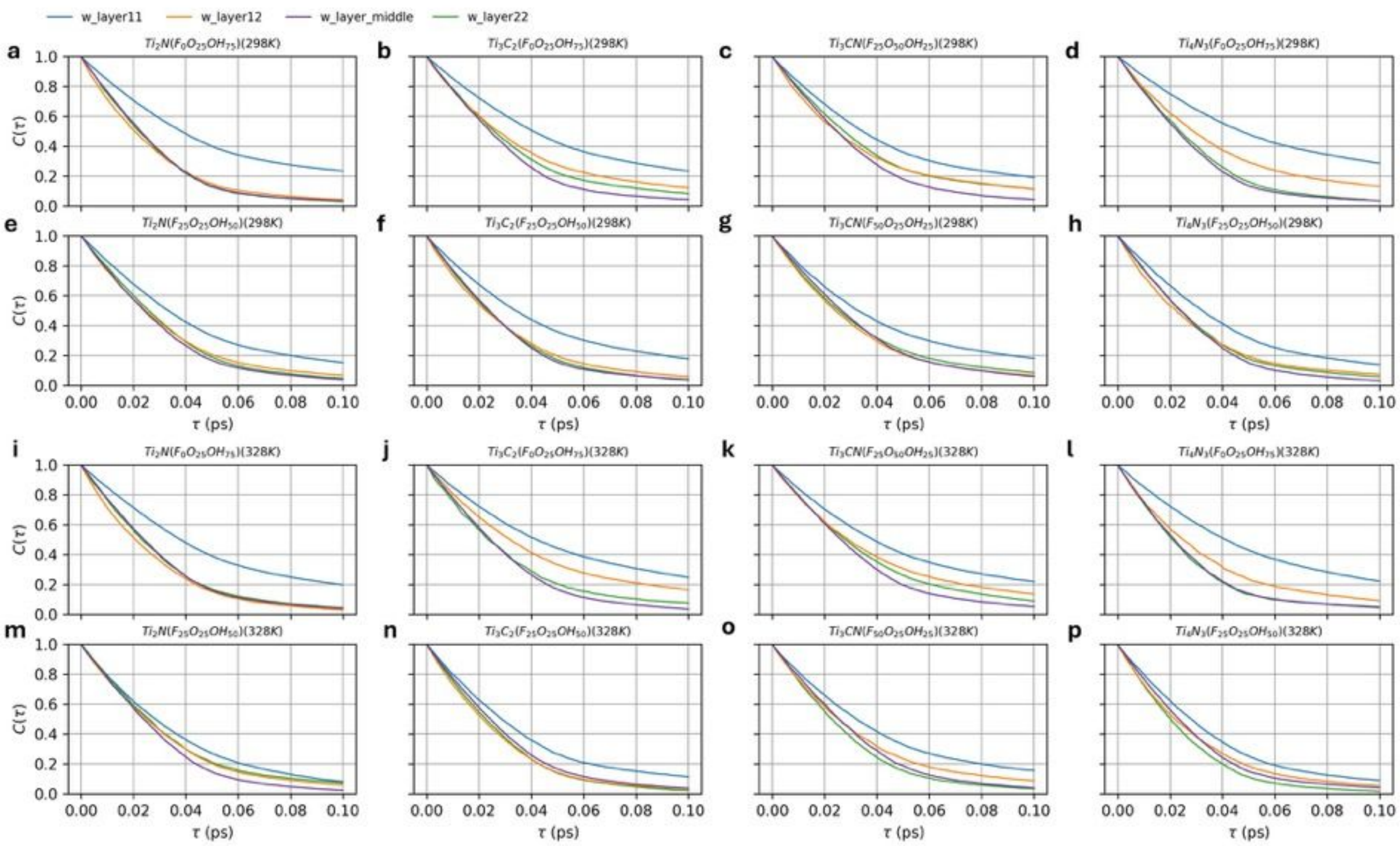

Figure S33. Intermittent time autocorrelation function, $C_I(\tau)$ =10 fs, for hydrogen bonds in different water regions near various functional MXene surfaces at 298 K (upper subplots), 328 K (lower subplots). The functions are calculated for strong HBs (2.7Å cutoff) formed exclusively within the first water layer (w_layer11), between the first and second water layers excluding within layers themselves (w_layer12), exclusively within the second water layer (w_layer22), and among all water molecules excluding the first two layers (w_layer_middle). The layer representation legends are shown as Figure 4i illustration. Overall, all decay trends are slower but the decay order between layers are the still same as continuous $C_I(\tau)$ =1 fs shown in **Figure S32**.

X. Discussion 8. Water Residence Time at MXene Interfaces

As a final, holistic measure of the overall strength of the water-surface interaction, we calculated the survival probability (SP) of water molecules in the vicinity of the MXene surface (within 2.5 Å upward and downward of slabs). The SP, as shown in **Figure 8**, represents the fraction of water molecules that start in this region and remain there continuously over time. A slower decay of the SP curve corresponds to a longer residence time and stronger water retention. All the analyzed functionalized MXenes are strongly hydrophilic, retaining more than half of their initial water layer over the duration of the simulation.

The analysis yields an unexpected result: the Ti_3CNT_x systems, which have a relatively low concentration of –OH groups, exhibit the best water retention ability at both 298 K and 328 K. This finding strongly supports the hypothesis that the quality of hydrogen bonds can be more important than their quantity. A surface with a high density of weaker H-bonds (e.g., high –OH) may allow for easy hopping and thus a shorter residence time, whereas a surface with fewer, but stronger and more localized, H-bonds (as in the case of Ti_3CNT_x) can lead to a more stable, less mobile water layer with a longer residence time.

Furthermore, the effect of temperature on water retention is anomalous for some systems. While most compositions exhibit the expected behavior of decreased SP at higher temperatures from 298K to 328K, $Ti_3C_2T_x(F_0O_{25}OH_{75})$ and $Ti_2NT_x(F_{25}O_{25}OH_{50})$ show a counter-intuitive increase in water survival probability upon heating from 298 K to 328 K. This paradoxical behavior suggests that the potential energy landscape for water on these specific surfaces could be relative "rugged." At the lower temperature, the system may be kinetically trapped in a glassy, disordered state with numerous local energy minima as fragile liquid.[13] The addition of thermal energy allows the water molecules to overcome small activation barriers and rearrange into a more globally stable, "locked-in" configuration that binds them more effectively to the surface. It could relate to the temperature-dependent balance fragile-to-strong transition of two strong liquid states including fast disordered water and slow disordered water state. This implies that for certain applications, operating at a moderately elevated temperature could unexpectedly enhance the stability and hydrophilicity of the MXene interface. The role of fluorine is also shown to be highly complex and non-monotonic, with its effect on water retention being dependent on the specific MXene substrate and the

operating temperature, highlighting a deeply intricate interplay between surface chemistry and thermal energy in dictating the overall water affinity of these materials.

When considering the effect of temperature, the most common expectation is that as temperature increases, water molecules should gain more thermal energy and escape the surface more easily, leading to a lower survival probability. This conventional behavior is observed for several MXene types, specifically $Ti_2NT_x(F_0O_{25}OH_{75})$, $Ti_3C_2T_x(F_{25}O_{25}OH_{50})$, $Ti_3CNT_x(F_{25}O_{50}OH_{25})$, and $Ti_4N_3T_x(F_{25}O_{25}OH_{50})$. For these materials, warming the system reduces its ability to hold onto nearby water. In a striking contrast, the opposite effect is seen for $Ti_3C_2T_x(F_0O_{25}OH_{75})$ and $Ti_2NT_x(F_{25}O_{25}OH_{50})$. For these two compositions, an increase in temperature actually causes the water survival probability to increase. This suggests that for these specific surfaces, thermal energy may trigger subtle rearrangements in the water's hydrogen-bond network, creating more stable hydrogen bonding conformations with MXene surface's OH groups that hold water more effectively.

The chemical nature of the MXene surface, particularly the concentration of fluorine groups, also has a profound and variable impact on water retention. The role that fluorine plays is highly dependent on both the specific MXene and the operating temperature. At room temperature, 298 K, increasing the amount of fluorine on the surface of $Ti_3C_2T_x(F_0O_{25}OH_{75})$ and $Ti_4N_3T_x(F_0O_{25}OH_{75})$ leads to an increase in water survival probability, with the effect being especially significant for $Ti_3C_2T_x$. However, for $Ti_2NT_x(F_0O_{25}OH_{75})$ and $Ti_3CNT_x(F_{25}O_{50}OH_{25})$, more fluorine results in a decrease in water retention. This shows that at room temperature, fluorine can either enhance or disrupt the surface's ability to bind with water, depending on the underlying MXene structure.

This complexity is further highlighted when the temperature is raised to 328 K. At this elevated temperature, many of the trends observed at room temperature shift or even reverse. For instance, the behavior of $Ti_2NT_x(F_0O_{25}OH_{75})$ flips completely; increasing its fluorine content now increases water survival probability instead of decreasing it. Likewise, $Ti_4N_3T_x(F_0O_{25}OH_{75})$ also reverses its trend, showing a decrease in water retention of first water layer with more fluorine. Meanwhile, the effect on $Ti_3C_2T_x(F_0O_{25}OH_{75})$ becomes negligible. These dramatic changes demonstrate that the function of a surface group like fluorine is not fixed and highlights a dynamic interplay between surface chemistry and thermal energy.

XI. References

(1) Kresse, G.; Furthmüller, J. Efficient iterative schemes for ab initio total-energy calculations using a plane-wave basis set. *Physical review B* **1996**, *54* (16), 11169.

(2) Perdew, J. P.; Burke, K.; Ernzerhof, M. Generalized Gradient Approximation Made Simple. *Physical Review Letters* **1996**, *77* (18), 3865-3868.

(3) Kresse, G.; Joubert, D. From ultrasoft pseudopotentials to the projector augmented-wave method. *Physical Review B* **1999**, *59* (3), 1758-1775.

(4) Lai, H.-E.; Yoo, R. M.; Djire, A.; Balbuena, P. B. Investigation of the Vibrational Properties of 2D Titanium Nitride MXene Using DFT. *The Journal of Physical Chemistry C* **2024**.

(5) Yesudoss, D. K.; Lai, H.-E.; Johnson, D.; Lee, M.; Reinhart, B.; Balbuena, P. B.; Djire, A. Lattice-Nitrogen-Mediated Chemistry Suppresses Hydrogen Evolution for Record Faradaic Efficiency in Ammonia Synthesis. *Journal of the American Chemical Society* **2025**.

(6) Yoo, R. M.; Ngozichukwu, B.; Yesudoss, D. K.; Lai, H.-E.; Arole, K.; Green, M. J.; Balbuena, P. B.; Djire, A. Vibrational Property Tuning of MXenes Revealed by Sublattice N Reactivity in Polar and Nonpolar Solvents. *Journal of the American Chemical Society* **2025**, *147* (12), 10104-10117.

(7) Monkhorst, H. J.; Pack, J. D. Special points for Brillouin-zone integrations. *Physical review B* **1976**, *13* (12), 5188.

(8) Grimme, S.; Ehrlich, S.; Goerigk, L. Effect of the damping function in dispersion corrected density functional theory. *Journal of computational chemistry* **2011**, *32* (7), 1456-1465.

(9) Meunier, M.; Robertson, S. Materials studio 20th anniversary. *Taylor & Francis:* **2021,** *47*, 537-539.

(10) Gowers, R. J.; Linke, M.; Barnoud, J.; Reddy, T. J. E.; Melo, M. N.; Seyler, S. L.; Domanski, J.; Dotson, D. L.; Buchoux, S.; Kenney, I. M. *MDAnalysis: a Python package for the rapid analysis of molecular dynamics simulations*; Los Alamos National Laboratory (LANL), Los Alamos, NM (United States), 2019.

(11) Michaud-Agrawal, N.; Denning, E. J.; Woolf, T. B.; Beckstein, O. MDAnalysis: a toolkit for the analysis of molecular dynamics simulations. *Journal of computational chemistry* **2011**, *32* (10), 2319-2327.

(12) Araya-Secchi, R.; Perez-Acle, T.; Kang, S.-g.; Huynh, T.; Bernardin, A.; Escalona, Y.; Garate, J.-A.; Martínez, A. D.; García, I. E.; Sáez, J. C. Characterization of a novel water pocket inside the human Cx26 hemichannel structure. *Biophysical journal* **2014**, *107* (3), 599-612.

(13) Shi, R.; Russo, J.; Tanaka, H. Origin of the emergent fragile-to-strong transition in supercooled water. *Proceedings of the National Academy of Sciences* **2018**, *115* (38), 9444-9449.

(14) Rivarolo, M.; Riveros-Godoy, G.; Magistri, L.; Massardo, A. F. Clean hydrogen and ammonia synthesis in Paraguay from the Itaipu 14 GW hydroelectric plant. *ChemEngineering* **2019**, *3* (4), 87.
